# Peptides from Marine-Derived Fungi: Chemistry and Biological Activities †

**DOI:** 10.3390/md21100510

**Published:** 2023-09-26

**Authors:** Salar Hafez Ghoran, Fatemeh Taktaz, Emília Sousa, Carla Fernandes, Anake Kijjoa

**Affiliations:** 1 H.E.J. Research Institute of Chemistry, International Center for Chemical and Biological Sciences, University of Karachi, Karachi 75270, Pakistan; s_hafezghoran@yahoo.com; 2 Department of Advanced Medical and Surgical Sciences, University of Campania “Luigi Vanvitelli”, 80138 Naples, Italy; f.taktaz@gmail.com; 3 Laboratório de Química Orgânica e Farmacêutica, Departamento de Ciências Químicas, Faculdade de Farmácia, Universidade do Porto and CIIMAR, Rua de Jorge Viterbo Ferreira 228, 4050-313 Porto, Portugal; esousa@ff.up.pt (E.S.); cfernandes@ff.up.pt (C.F.); 4 ICBAS—Instituto de Ciências Biomédicas Abel Salazar, Universidade do Porto and CIIMAR, Rua de Jorge Viterbo Ferreira 228, 4050-313 Porto, Portugal

**Keywords:** marine-derived peptides, linear peptides, cyclic peptides, depsipeptides, marine-derived fungi, antibacterial activity, cytotoxicity

## Abstract

Marine natural products are well-recognized as potential resources to fill the pipeline of drug leads to enter the pharmaceutical industry. In this circumstance, marine-derived fungi are one of the unique sources of bioactive secondary metabolites due to their capacity to produce diverse polyketides and peptides with unique structures and diverse biological activities. The present review covers the peptides from marine-derived fungi reported from the literature published from January 1991 to June 2023, and various scientific databases, including Elsevier, ACS publications, Taylor and Francis, Wiley Online Library, MDPI, Springer, Thieme, Bentham, ProQuest, and the Marine Pharmacology website, are used for a literature search. This review focuses on chemical characteristics, sources, and biological and pharmacological activities of 366 marine fungal peptides belonging to various classes, such as linear, cyclic, and depsipeptides. Among 30 marine-derived fungal genera, isolated from marine macro-organisms such as marine algae, sponges, coral, and mangrove plants, as well as deep sea sediments, species of *Aspergillus* were found to produce the highest number of peptides (174 peptides), followed by *Penicillium* (23 peptides), *Acremonium* (22 peptides), *Eurotium* (18 peptides), *Trichoderma* (18 peptides), *Simplicillium* (17 peptides), and *Beauveria* (12 peptides). The cytotoxic activity against a broad spectrum of human cancer cell lines was the predominant biological activity of the reported marine peptides (32%), whereas antibacterial, antifungal, antiviral, anti-inflammatory, and various enzyme inhibition activities ranged from 7% to 20%. In the first part of this review, the chemistry of marine peptides is discussed and followed by their biological activity.

## 1. Introduction

Fungi are ubiquitous micro-organisms living symbiotically or endophytically with almost all viable resources. Marine fungal strains exist in various habitats and play a significant ecological role due to their capacity to produce a great variety of specialized metabolites such as alkaloids, polyketides, terpenoids, and peptides [1]. Peptides are a class of promising secondary metabolites with relevant pharmacological/biological activities due to their low toxicity and good affinity to a variety of important targets, such as G-protein-coupled receptors (GPCRs) or/and ion channels. These bioactive peptides consist of a series of well-ordered amino acids with approximate molecular weights of 500–5000 Da. Most significantly, bioactive marine-derived peptides exhibit a myriad of biological activities *viz.* antioxidant, antibacterial, antiviral, antifungal, antidiabetic, antiproliferative, anti-inflammatory, anticancer, anticoagulant, anti-hypertensive, antiobesity, anti-Alzheimer, and calcium-binding activities [2,3,4,5]. Due to their interesting bioactivities and specificity, peptides are considered to have a high potential for design, and even for modification, in order to obtain peptide-based drug candidates for clinical trial purposes. However, besides the expensive process of peptide synthesis, the main drawback of synthetic peptides is their poor physico-chemical property and enzymatic stability, resulting in less bioavailability and short circulating plasma half-life that limit their success in the body [6,7]. In terms of the production of marine peptides, there are three distinct categories including (i) naturally occurring peptides isolated directly from marine resources; (ii) naturally occurring peptides derived from the fermentation of marine-derived micro-organisms; and (iii) peptides derived from the hydrolysis of proteins obtained from marine organisms using various digestive enzymes, i.e., α-chymotrypsin, pepsin, papain, trypsin, and other proteases [8]. However, compared to naturally occurring human peptides, the structures of marine-derived peptides are different in either the backbone or side chains. Associated with unique conditions of the marine environment, marine-derived peptides are valuable candidates for drug design since they are mostly enzymatically and thermally stable [9]. The biosynthesis of marine-derived peptides and terrestrial peptides is a complex and varied process that can involve multiple enzyme systems, depending on the specific organisms and peptides being produced. For instance, some marine organisms produce peptides through nonribosomal peptide synthetases (NRPS) or nonribosomal peptide synthetase/polyketide synthase hybrids (NRPS/PKS), which are large, multi-domain enzymes that catalyze the assembly of amino acids into peptides. Similarly, some terrestrial organisms, such as fungi, produce peptides through NRPS. However, there are also examples of marine- and terrestrial-derived peptides that are produced by simpler biosynthetic pathways, such as ribosomal synthesis, which involve a direct translation of mRNA into a peptide chain by ribosomes, and, consequently, undergo post-translational modification [10]. Since ribosomally synthesized and post-translationally modified peptides (RiPPs) include only 20 unmodified proteinogenic amino acids, whereas nonribosomal peptides (NRPs) contain more than 100 non-coded non-proteinogenic amino acids, the chemical diversity of NRPs is higher than that of RiPPs. However, the RiPPs can better bind to the targets due to their restricted conformational flexibility originated during post-translational/co-translational modifications, thus making them appropriate for pharmaceutical applications [11]. Peptide-producing eukaryotic microbes generally reside in sponges, algae, dinoflagellates, and diatoms. Marine-derived fungi are among the micro-organisms of great interest and receive much attention because of their capacity to generate a great diversity of secondary metabolites with interesting biological and pharmacological properties [12,13].

To the best of our knowledge, there were several reviews covering only particular peptides from some specific fungal species [5,9,14,15,16]. Therefore, we hereby present a comprehensive review aiming to provide the information on structural diversity and biological/pharmacological activities of marine-derived peptides that have been reported from all the peptide-producing marine fungal strains from January 1991 to June 2023. Moreover, the stereochemistry of amino acids and non-amino acid moieties was briefly referred to in this review since this subject was thoroughly discussed in a recent review by Fernandes et al. [17]. The databases used to search for the keywords were SciFinder, Pubmed, Web of Science, Scopus, the Marine Pharmacology website, and Google Scholar.

## 2. The Importance of Marine-Derived Peptides in Drug Discovery

**Figure 1:**
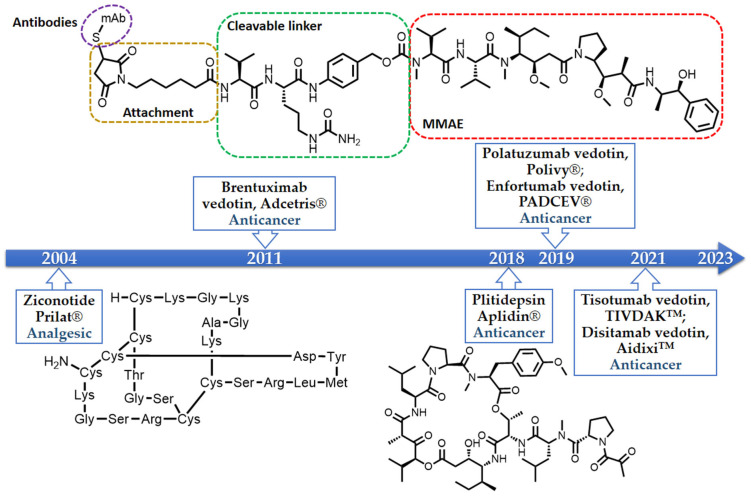
FDA-approved marine peptide-based drugs [24].

A large number of in vitro and in vivo investigations demonstrated the safety and efficacy of peptides. Naturally occurring and chemically modified marine peptides have demonstrated their potential in a myriad of therapeutic areas, acting as hormones, growth factors, neurotransmitters, ion channel ligands, antihypertensive, anti-inflammatory, and anti-infective agents, and continue to fuel the drug pipeline [4]. The best examples of the Food and Drug Administration (FDA)-approved marine-derived peptides, which are on the market, are ziconotide (Prilat^®^), brentuximab vedotin (Adcetris^®^), polatuzumab vedotin (Polivy^®^), enfortumab vedotin (PADCEV^TM^), disitamab vedotin (Aidixi^TM^), tisotumab vedotin (TIVDAK^TM^), and plitidepsin (Aplidin^®^) (Figure 1) [18,19,20,21,22,23,24,25]. Ziconotide, a synthetic version of ω-conotoxin MVIIA, the cyclic peptide derived from the fish-eating marine cone snail *Conus magus*, is a *N*-type calcium channel blocker and is used as an analgesic for chronic pain [18]. Brentuximab vedotin, an antibody-drug conjugate medication used for the treatment of relapsed or refractory Hodgkin’s lymphoma (HL) and systemic anaplastic large cell lymphoma (ALCL), consists of monomethyl auristatin E (MMAE), which is a synthetic analogue of a marine-derived peptide, dolastatin 10, conjugated with a chimeric monoclonal antibody, cAC10, that targets CD30 protein which is expressed abundantly in cancer cells [19]. On the other hand, polatuzumab vedotin, which was approved by the FDA in 2019 for the treatment of non-Hodgkin’s lymphoma, chronic lymphovytic leukemia, and diffuse large B-cell lymphoma, consists of MMAE conjugated with a monoclonal antibody targeting the CD79b protein [20].

Enfortumab vedotin is an antibody and microtubule inhibitor conjugate designed to target nectin-4, a cell adhesion molecule found at high levels in urothelial cancer. This drug is directed at cancer cells, particularly in locally advanced or metastatic urothelial cancer cases where prior treatment involved a programmed death receptor-1 (PD-1) or programmed death ligand-1 (PD-L1) inhibitor and platinum-containing chemotherapy [21]. Disitamab vedotin is another antibody-drug conjugate that pairs a monoclonal antibody against human epidermal growth factor receptor 2 (HER2) with MMAE via a cleavable linker. This drug targets solid tumors like gastric cancer, HER2 overexpressing gastric carcinoma, urothelial carcinoma, and advanced breast cancer [22].

Tisotumab vedotin is an antibody-drug conjugate featuring a fully human monoclonal antibody (TF-011) directed at tissue factor, combined with MMAE to target tissue-factor-positive tumors. It is applied in the case of metastatic cervical cancer post-chemotherapy progression [23]. Plitidepsin (Aplidin^®^), a cyclic depsipeptide isolated from a Meditranean tunicate, *Aplidium albicans*, has passed the clinical trial studies as an anticancer drug for multiple melanoma, leukemia, and lymphoma [25].

Other marine peptides in the clinical trial phases include kahalalide F, elisidepsin (PM02734), tasidotin (ILX-651), glembatumumab vedotin (CDX-011), soblidotin (TZT-1027), E7974, HTI-286, XEN-2174, salinosporamide A, dalatazide (Shk-186), Vc1.1, RgIA, and RgIA4 (KCP-400) [9,26,27,28].

**Figure 2:**
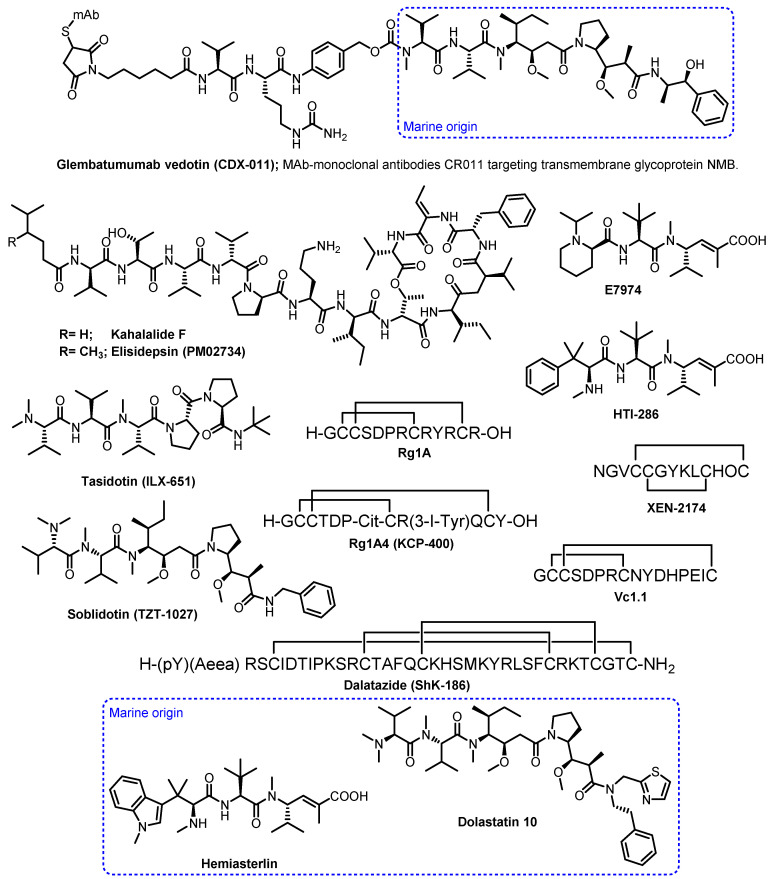
Marine-derived peptides and their derivatives which are in clinical trials.

Kahalalide F (Figure 2), a synthetic version of a cyclic depsipeptide, isolated from a mollusk *Elysia rufescens* and its green algal diet *Bryopsis* sp., was being evaluated in a phase II clinical trial in malignant melanoma patients. Although kahalalide F was a well-tolerated and safe chemotherapy regimen, the trial was closed due to the lack of objective response in patients with malignant melanoma [29].

Elisidepsin (PM02734; Figure 2) is a synthetic analogue of kahalalide F, which has passed a phase I clinical study against malignant-solid tumors, and is in phase II clinical studies [30].

Tasidotin (ILX-651; Figure 2), an orally active synthetic microtubule-targeted derivative of dolastatin-15, is currently undergoing clinical evaluation for cancer treatment [31]. Glembatumumab vedotin (CDX-011) and soblidotin (TZT-1027), another dolastatin 10 analogues (Figure 2), are in phase I/II clinical trials for the treatment of breast cancer and soft tissue sarcoma, respectively [32,33].

E7974 and HTI-286 (Figure 2) are synthetic analogues of a cytotoxic tripeptide, hemiasterlin, which is derived from a marine sponge *Hemiasterella minor*. E7974 was in a phase I study for colorectal, prostate, and larynx carcinomas, while HTI-286 is preclinically applied in cases of metastatic prostate cancer [34,35].

XEN-2174 (Figure 2) is a synthetic peptide that binds specifically to the norepinephrine transporter, thus causing an inhibition of norepinephrine uptake. XEN-2174 has progressed to a phase IIb trial; however, it showed dose-limiting toxicity in pharmacodynamics and cerebrospinal fluid pharmacokinetics assays. Thus, it is unlikely that this conotoxin can be used for the treatment of acute pain in humans [36].

Shk-186 (Figure 2) is a 37-amino-acid synthetic peptide that is a specific inhibitor of the voltage-gated Kv1.3 potassium channel. It is a derivative of ShK, which was originally isolated from the venom of the sea anemone *Stichodactyla helianthus*. Shk-186 had a 100-fold improvement in selectivity for KV1.3 over the voltage-gated K^+^, KV1.1, KV1.4, and KV1.6, and was found to ameliorate autoimmune diseases such as multiple sclerosis and rheumatoid arthritis in human models. Interestingly, ShK-186 was found to have a long half-life at the site of injection, which produced sustained high pM levels in plasma, thus minimizing the need to improve its pharmacokinetic properties. An investigational new drug (IND) was filed by Kineta and approved by the FDA in 2012. ShK-186 has been allocated the generic name dalazatide, and completed phase 1a and 1b trials in 2016. The results of the phase Ib trial for psoriasis were reported recently, and showed that dalazatide was well-tolerated, without serious adverse events, and reduced psoriatic skin lesions. It is positioned to begin phase IIa trials [26].

Conotoxin Vc1.1 (Figure 2) is a modified synthetic form of the 16-amino-acid α-conotoxin Vc1a from the venom of *Conus victoriae*, which exhibited exceptional activity in animal models of neuropathic pain, causing long-lasting analgesia for at least 24 h following a single subcutaneous dose. Metabolic Pharmaceuticals started to develop Vc1.1 for the treatment of neuropathic pain and had progressed through double-blinded phase 1 clinical trials. However, a phase IIa clinical trial in patients with sciatic neuropathic pain was abandoned because Vc1.1 was significantly less potent on the human form of its presumed target, the α9α10 nicotinic acetylcholine receptor (nAChR), than had earlier been observed for the rat isoform. Therefore, further commercial development was halted [27].

RgIA (Figure 2) is a 13-residue two intramolecular disulfide peptide of the α-conotoxin class, originally isolated from a sea snail, *Conus regius*. Although a-conotoxin antagonists of α9α10 nAChRs have been proposed as potential analgesics for the treatment of neuropathic pain, they are less potent on human than rodent nAChRs, thus limiting their translational application. To overcome RgIA limitations, Kineta Inc., in collaboration with researchers from the University of Utah at Salt Lake City, has conducted SAR studies on RgIA which led to the development of RgIA4 (KCP-400; Figure 2), a peptide that exhibits high potency for both human and rodent α9α10 nAChRs, and was at least 1000-fold more selective for α9α10 nAChRs over all other molecular targets tested, including opioid and GABA_B_ receptors [26]. Besides conducting safety and efficiency studies for KCP-400, Kineta Inc. also developed a non-opioid KCP-506 and has recently completed a single ascending dose (SAD) clinical study. KCP506 may potentially be an effective treatment for many types of chronic neuropathic pain including radiculopathy, chemotherapy-induced peripheral neuropathy, and diabetic neuropathy. KCP506 offers the potential for a disease-modifying therapy that may slow or halt the progression of chronic pain [28].

## 3. Peptides from Marine-Derived Fungi

A comprehensive literature survey of peptides covering the period of January 1991–June 2023 was undertaken. To facilitate the discussion of the reported peptides from marine-derived fungi, we classify them as linear peptides, cyclic peptides, cyclic depsipeptides, and, in some cases, more complicated structures [37].

### 3.1. Linear Peptides

Linear peptides are peptides whose amino acids are linked linearly in sequence. They have one free amino end and one free carboxyl end.

#### 3.1.1. Linear Dipeptides

**Figure 3:**
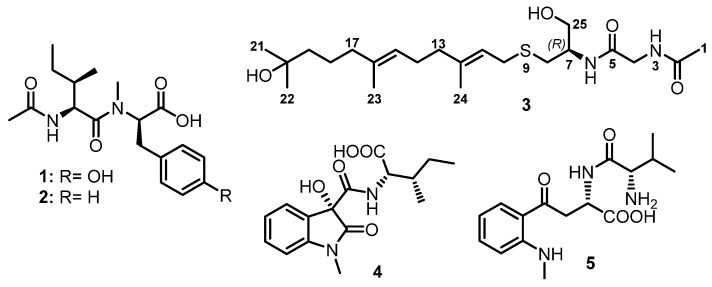
Structures of **1**–**5**.

Liang et al. reported two acylated linear dipeptides, simplicilliumtides G (**1**) and H (**2**) (Figure 3), from the culture broth extract of a deep-sea-associated fungus, *Simplicillium obclavatum* EIODSF 020, collected in the Indian Ocean. Extensive 1D and 2D NMR spectral analysis revealed the presence of an acetyl group, Ile, and *N*-Me-Tyr residues in **1**, while **2** contains the *N*-Me-Phe residue instead of the *N*-Me-Tyr residue. The absolute configurations of the Ile, *N*-Me-Tyr, and *N*-Me-Phe residues in **1** and **2** were determined by Marfey’s method as L-Ile, *N*-Me-D-Tyr, and *N*-Me-D-Phe, respectively [38].

Coniosulfide E (**3**) (Figure 3), a rare linear dipeptide containing an unusual cysteinol moiety, was isolated from the ethyl acetate (EtOAc) culture extract of a deep-sea shrimp-derived fungus, *Aspergillus unguis* IV17-109. In order to determine the absolute configuration at C-7, the authors synthesized **3** using farnesyl chloride with L- and D-Cys, and compared the optical rotations of the two obtained products with the isolated compound. Since the optical rotation of **3** (${\left[\alpha \right]}_{D}^{20}$ −100; *c* 0.3, MeOH) was similar to that of the L-Cys-containing product (${\left[\alpha \right]}_{D}^{20}$ −110; *c* 0.3, MeOH), the absolute configuration at C-7 in **3** was deduced as 7*R*. The *E*-configuration of Δ^11^ and Δ^15^ was assigned by NOESY correlations [39].

Chemical investigation of the culture extract of a marine-derived fungus, *Penicillium* sp. SCSIO 41512, afforded two undescribed linear dipeptides, penicamides A (**4**) and B (**5**) (Figure 3). Their structures were established by a combination of 1D and 2D NMR spectral and high-resolution electrospray ionization mass spectrometry (HRESI-MS) analyses. The absolute configurations of the stereogenic carbons were established by Marfey’s method and quantum chemical calculations [40].

#### 3.1.2. Linear Tripeptides

**Figure 4:**
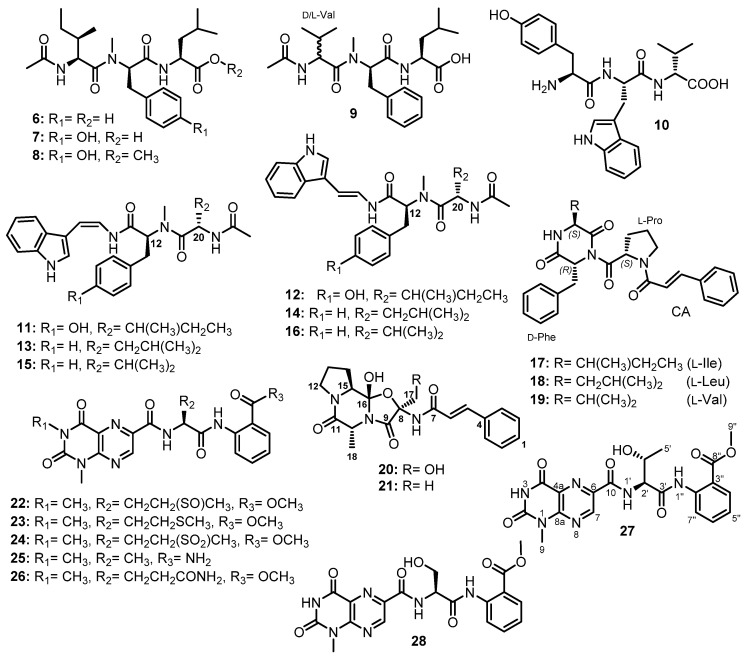
Structures of **6**–**28**.

The fermentation broth extract of *S. obclavatum* EIODSF 020 also furnished the linear acylated tripeptides, simplicilliumtides C-F (**6**–**9**) (Figure 4). HMBC correlations and Marfey’s analysis were used to identify their amino acid residues, amino acid sequences, and their absolute configurations, respectively, as Ac-L-*allo*-Ile-*N*-Me-D-Phe-L-Leu for **6**; Ac-L-*allo*-Ile-*N*-Me-D-Tyr-L-Leu for **7**; Ac-L-*allo*-Ile-*N*-Me-D-Tyr-L-Leu for **8** (replacing the COOH group of Leu with COOMe); and Ac-D/L-Val-*N*-Me-D-Phe-L-Leu for **9**, which contains a mixture of D- and L-Val [38].

Another linear tripeptide, aspergillipeptide E (**10**) (Figure 4), was isolated from the culture broth extract of a marine-derived fungus, *Aspergillus* sp. SCSIO 41501, obtained from a gorgonian, *Melitodes squamata*, which was collected from the South China Sea. Then, 1D and 2D NMR data and Marfey’s method were used to establish the structure of **10** as D-Tyr-L-Trp-D-Val [41].

Two isomeric linear tripeptides, aspergillamides C (**11**) and D (**12**), together with the previously reported aspergillamides A (**13**) and B (**14**), *cis*-L-phenylalaninamide (**15**), and *trans*-L-phenylalaninamide (**16**) (Figure 4), were obtained from the mycelium extract of *A. terreus* SCSIO41008, isolated from a marine sponge, *Callyspongia* sp., which was collected from the seaside in Guangdong Province, China. The absolute configurations at C-12 and C-20 in **11** and **12** were established as 12*S*,20*S*, which are the same as those of C-12 and C-20 of **13**–**16**, by a comparison of the calculated and experimental electronic circular dichroism (ECD) spectra [42].

A chemical investigation of the EtOAc extract of the mycelium of a marine-derived fungus, *A. terreus* LM.5.2, isolated from the leaves of a mangrove tree, *Kandelia candel*, which were collected from coast of Khanh Hoa province, South China Sea, Vietnam, resulted in the isolation of three unreported tripeptides containing a cinnamic acid (CA) moiety, asterripeptides A-C (**17**–**19**) (Figure 4). The structures of the amino acid residues of **17**–**19** were elucidated by 1D and 2D NMR spectral analysis and their sequences were determined by HMBC and ROESY correlations as Phe-Ile-Pro, Phe- Leu-Pro, and Phe-Val-Pro, respectively The absolute configurations of the amino acid residues were established by Marfey’s method and chiral HPLC as D-Phe-L-Ile-L-Pro, D-Phe-L-Leu-L-Pro, and D-Phe-L-Val-L-Pro [43].

Two unreported tripeptides with an unusual 5/6/5 heterocyclic scaffold bearing a *N*-*trans*-cinnamoyl moiety, talaropeptins A (**20**) and B (**21**) (Figure 4), were isolated from the EtOAc culture extract of a marine-derived fungus, *Talaromyces purpureogenus* CX11, isolated from the seaweed *Grateloupia filicina* C. Ag (Wulf.), which was collected from Zhoushan, Zhejiang province, China. The presence of a *trans-*cinnamoyl moiety (cin), Pro, Ala, and α-oxygenated Ser (α-*O*-Ser) in **20** was evidenced by 1D and 2D NMR spectral analyses. The HMBC and ROESY correlations showed the amino acid sequence as cin-Ser-Ala-Pro. The relative configurations of the stereogenic carbons in **20** were determined as 8R*,10R*,15S*,16S* by ROESY correlations while their absolute configurations were established as 8*R*,10*R*,15*S*,16*S* by Marfey’s analysis and comparison of the calculated and experimental ECD spectra [44].

On the other hand, the 1D and 2D NMR spectral analysis revealed that the structure of **21** is similar to **20** except for the presence of Ala instead of Ser. The absolute configurations of the stereogenic carbons in **21** were assumed to be the same as those of **20** due to their shared biosynthetic origin. This hypothesis was supported by a comparison of the calculated and experimental ECD spectra. The structures of **20** and **21** suggested that their biosynthesis is carried out predominantly by NRPS machinery [44].

Meyer et al. reported the isolation of penilumamide (**22**) (Figure 4), a lumazine peptide containing L-methionine sulfoxide and anthranilic acid (2-aminobenzoic acid, Abz) ester, from the fermentation broth extract of *Penicillium* sp. (strain CNL-338), isolated from a red alga, *Laurencia* sp., which was collected in the Bahamas Islands [45].

Penilumamides B–D (**23**–**25**) (Figure 4) were isolated, together with **22**, from the mycelial and culture broth extracts of *Aspergillus* sp. SX-20090B15, isolated from the inner tissue of a fresh gorgonian, *Muricella abnormaliz*, which was collected from the Xisha Islands coral reef on the South China Sea. Interestingly, **22**, **24**, and **25** were isolated from the culture in a normal potato glucose liquid medium, whereas **23** was only isolated from the L-Met supplemented culture. The authors have found that **22** and **23** were unstable when exposed to air. Moreover, when **23** was exposed to air at room temperature, **22** was detected after a few days and **24** appeared several days later, suggesting that methionine sulfoxide in **22** and methionine sulfone in **24** are formed by the oxidation of Met in **23** [46].

The EtOAc extract of the mycelium of *Aspergillus* sp. (33241), isolated from the mangrove plant, *Bruguiera sexangula* var. *rhynchopetala*, collected from the South China Sea, also furnished another lumazine-containing peptide, aspergilumamide A (**26**) (Figure 4), in addition to **22**. The stereochemistry of L-Glu in **26** was determined by Marfey’s analysis [47].

Further lumazine-containing peptides, terrelumamides A (**27**) and B (**28**) (Figure 4), were isolated from the culture broth extract of *A. terreus* FA009, which was obtained from a marine sediment collected off the shore of Jeju Island, Korea. Both **27** and **28** contain 1-methyllumazine-6-carboxylic acid and Abz methyl ester. The difference between **27** and **28** is the presence of Thr in the former and Ser in the latter. The configurations of both Thr and Ser were determined as L by the advanced Marfey’s method [48].

#### 3.1.3. Linear Tetra- and Hexapeptides

**Figure 5:**
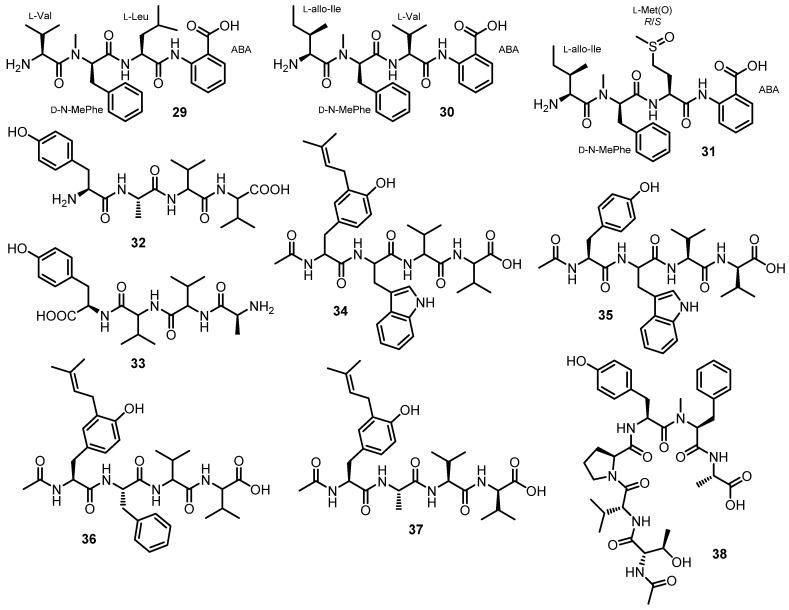
Structures of **29**–**38**.

Simplicilliumtides A (**29**) and B (**30**) (Figure 5), linear tetrapeptides containing Abz residue, were isolated from the combined mycelia and broth extracts of a deep-sea-derived fungus, *S. obclavatum* EIODSF 020, collected in the Indian Ocean. The amino acid sequences of both peptides were established by HMBC correlations, whereas Marfey’s analysis was used to determine the absolute configurations of the amino acid residues. Consequently, the structures of **29** and **30** were established as L-Val-*N*-Me-D-Phe-L-Leu-Abz, and L-*allo*-Ile-*N*-Me-D-Phe-L-Val-Abz, respectively [38].

Further chemical investigation of the same fungus led to the isolation of another linear tetrapeptide, simplicilliumtide I (**31**) (Figure 5); 1D and 2D NMR spectral analysis, in combination with low-resolution two-stage mass spectrometer equipped with electrospray ionization (LR-ESI-MS/MS), established the amino acid sequence as Ile-*N*-Me-Phe-Met sulfoxide [Met(O)], while Marfey’s method and HPLC established the configurations of the amino acids as L-*allo*-Ile, *N*-Me-D-Phe, and L-Met(O), respectively [49].

Ma et al. described the isolation of aspergillipeptides F (**32**) and G (**33**) (Figure 5) from the culture broth extract of a marine-derived fungus, *Aspergillus* sp. SCSIO 41501, isolated from a gorgonian, *Melitodes squamata*, which was collected from the South China Sea. HMBC correlations were used to establish the amino acid sequence of **32** as Tyr-Ala-Val-Val, and of **33** as Tyr-Val-Val-Ala. Since Marfey’s method revealed the presence of both D- and L-Val, it was not possible to identify which one is L and which one is D in both compounds [41].

Aspergillipeptides H–K (**34**–**37**) (Figure 5) were obtained from the extract of a solid rice culture of *Aspergillus* sp. SCSIO 41501, isolated from the gorgonian *M. squamata* Nutting, which was collected from the South China Sea. A detailed analysis of HMBC correlations established the sequence of the amino acid residues in **35**–**37** as *N*-Ac-Tyr-Trp-Val-Val, *N*-Ac-isopentenyl-Tyr-Ala-Val-Val, and *N*-acetyl-isopentenyl-Tyr-Phe-Val-Val, respectively. Since Marfey’s method was not able to completely assign the stereochemistry of the amino acids in **35**–**37**, only D-Tyr, L-Val, and L-Val (for **35**), L-Ala, L-Val, and L-Val (for **36**), and L-Phe (for **37**) were determined, while the configurations of the remaining amino acids were unassigned. Although the sequence of the amino acids in **34** was established as *N*-acetyl-isopentenyl-Tyr-Trp-Val-Val, their configurations were not assigned [50].

Chemical investigation of a semi-solid culture extract of *A. ochraceopetaliformis*, which was collected from an underwater sediment obtained off the coast of Jaeju-do Island, Korea, resulted in the isolation of FJ120DPB (**38**) (Figure 5). The structure of **38** was elucidated as *N*-Me-L-Phe-L-Ala-Ac-L-Thr-D-Val-L-Pro-L-Tyr by a combination of HMBC correlations and Mafrey’s analysis [51].

#### 3.1.4. Linear Octapeptides

**Figure 6:**
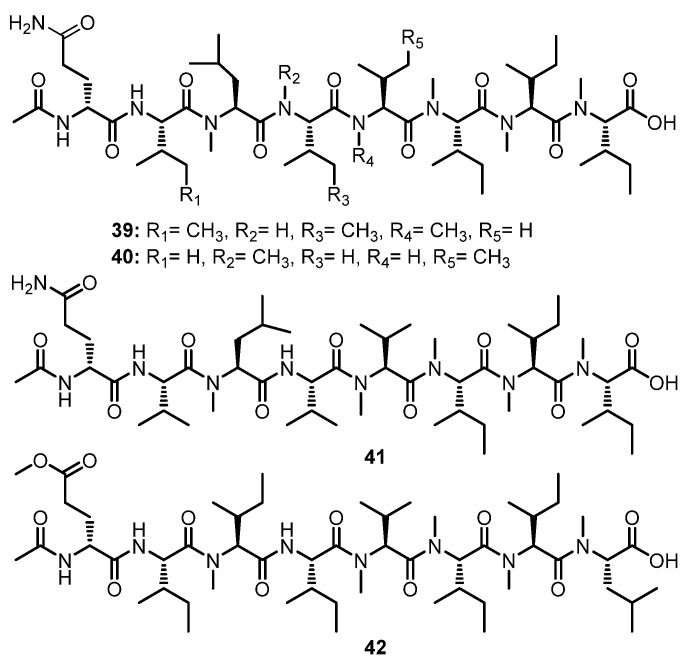
Structures of **39**–**42**.

Two *N*-methylated linear octapeptides, RHM1 (**39**) and RHM2 (**40**) (Figure 6), were isolated from the culture extract of an atypical *Acremonium* sp. (UCSC coll. no. 021172 cKZ), isolated from a marine sponge, *Teichaxinella* sp. (coll. no. 02172), which was collected at Milne Bay, Papua New Guinea. The identification of amino acid residues and their sequences of both compounds were based on the assembly of various fragments whose structures were established by a combination of 2D NMR spectra and fragments ions from the electrospray ionization mass spectrum (ESIMS). The configurations of the amino acid residues in **39** were determined by Mafrey’s method. Thus, the structure of **39** was established as Ac-(*R*)-Gln-(2*S*,3*S*)-Ile-(*S*)-*N*-Me-Leu-(2*S*,3*S*)-Ile-(*S*)-*N*-Me-Val-(2*S*,3*S*)-*N*-Me-Ile-(2*S*,3*S*)-*N*-Me-Ile-(2*S*,3*S*)-*N*-Me-Ile-OH. On the other hand, the configuration of the amino acid residues in **40** were postulated to be the same as those in **40** on the basis of their biosynthetic origin which established the structure of **40** as Ac-(*R*)-Glu-(*S*)-Val-(*S*)-*N*-Me-Leu-(*S*)-*N*-Me-Val-(2*S*,3*S*)-Ile-(2*S*,3*S*)-*N*-Me-Ile-(2*S*,3*S*)-*N*-Me-Ile-(2*S*,3*S*)-*N*-Me-Ile-OH [52].

Further investigation of the same fungus by the same research group resulted in the isolation of another two *N*-methylated linear octapeptides, RHM3 (**41**) and RHM4 (**42**) (Figure 6). The absolute configurations of the amino acids in **41** and **42** were concluded to be the same as those of **39** and **40** on the basis of their biosynthetic origin [53].

#### 3.1.5. Linear Nonapeptides

**Figure 7:**
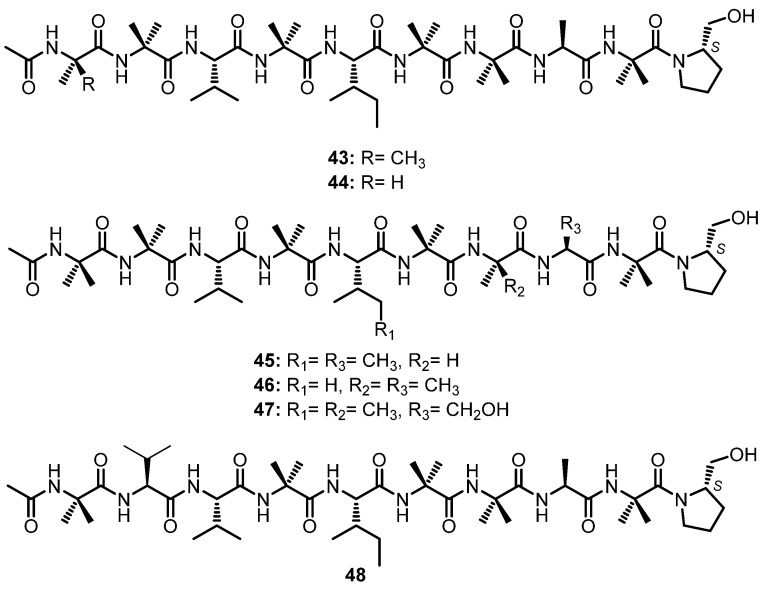
Structures of **43**–**48**.

A chemical investigation of the fermentation extract of *Trichoderma asperellum*, obtained from a sediment of the Antarctic Penguin Island, furnished six peptaibols, asperelines A–F (**43**–**48**), containing nine amino acid residues (Figure 7). Peptaibols are characterized by featuring an abundance of α-aminoisobutyric acid (Aib) residue, the *N*-acyl terminus such as an acetyl group, and the *C*-terminus which contains an amino alcohol residue [54].

Compounds **43**–**46** and **48** contain various units of Aib, Val, Ile, Ala, and the amino alcohol protinol, while **47** contains Ser instead of Ala. The sequences of the amino acids in **43**–**48** were determined using 2D NMR, especially HMBC correlations together with the fragmentation ions from the ESIMS/MS analysis, while the configuration of each amino acid is determined by comparison of the ^1^H NMR spectra of the complex formed by each amino acid, obtained by hydrolysis of the peptaibol, with a chiral reagent, Ru(D_4_-Por*)CO, with the ^1^H NMR data of the complex between the L/D-standard amino acids. The configurations of Ala, Val, Ile, and Ser were established as L, while the configuration of the stereogenic carbon of prolinol was established as *S* [54].

#### 3.1.6. Linear Undecapeptides

**Figure 8:**
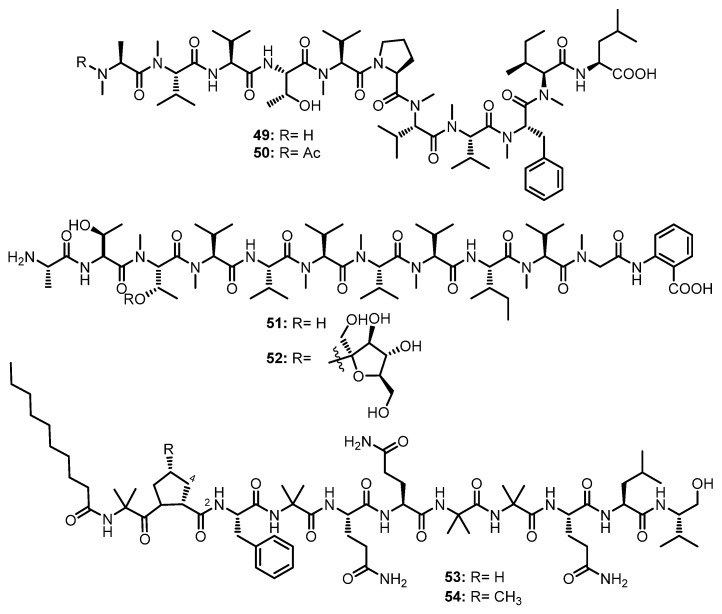
Structures of **49**–**54**.

A 24-well microbioreactor cultivation analysis (MATRIX) using eleven different media and three phases (solid agar, as well as static and shaken broth), in combination with ultra-high-performance liquid chromatography with diode-array detection (UHPLC-DAD) and the ultra-high performance liquid chromatography-quadrupole time-of-flight tandem mass spectrometry (UHPLC-QTOF-MS/MS) analyses, resulted in the isolation of two *N*-methylated linear undecapeptides, talaropeptides A (**49**) and C (**50**) (Figure 8), from the culture extract of *Talaromyces* sp. CMB-TU011, isolated from an unidentified tunicate, which was collected in New South Wales, Australia. In the 1D NMR spectra of **49** and **50**, the overlapping of resonances did not allow us to assign their amino acid residues. Although the partial amino acid sequences were determined by diagnostic HMBC and ROESY correlations, the linkage of amino acid fragments remained to be solved. A further diagnostic of the UHPLC-QTOF-MS/MS fragmentation patterns provided two consolidated partial sequences for **49** (i.e., *N*-Me-Ala^1^-*N*-Me-Val^2^-Val^3^-Thr^4^-*N*-Me-Val^5^ and *N*-Me-Val^8^-*N*-Me-Phe^9^-*N*-Me-Ile^10^-Leu^11^) and a consolidated partial sequence for **50** (i.e., *N*-Me-Val^8^-*N*-Me-Phe^9^-*N*-Me-Ile^10^-Leu^11^). Moreover, all the amino acid residues in **49** and **50** were determined as L-configured using C_3_ and C_18_ Marfey’s methods. The final assembly of the partial sequences of amino acids allowed us to reach the complete structures of **49** and **50** as *N*-Me-L-Ala^1^-*N*-Me-L-Val^2^-L-Val^3^-L-Thr^4^-*N*-Me-L-Val^5^-L-Pro^6^-*N*-Me-L-Val^7^-*N*-Me-L-Val^8^-*N*-Me-L-Phe^9^-*N*-Me-L-Ile^10^-L-Leu^11^ for **49**, and *N*-Me-*N*-Ac-L-Ala^1^-L-Val^2^-L-Val^3^-L-Thr^4^-*N*-Me-L-Val^5^-L-Pro^6^-*N*-Me-L-Val^7^-*N*-Me-L-Val^8^-*N*-Me-L-Phe^9^-*N*-Me-L-Ile^10^-L-Leu^11^ for **50** [55].

The mycelial extract of a fungal strain K063, obtained from a red alga, *Ceratodictyon spongiosum*, which was collected off Seragaki Beach in Okinawa, furnished dictyonamide A (**51**), and its glycosylated analog, dictyonamide B (**52**) (Figure 8). The amino acid residues and their sequence in **51** and the presence of the Abz moiety were determined by a high-resolution fast atom bombardment mass spectrum (HRFAB-MS), and 1D and 2D NMR spectral analysis, as well as an amino acid analysis of its hydrolysate. Marfey’s method revealed that all the amino acid residues, except for *N*-Me-Thr, have an L-configuration. The configuration of *N*-Me-Thr was resolved as L by chiral HPLC analysis. Thus, the structure of **51** was determined as L-Ala-L-Thr- *N*-Me-L-Thr-*N*-Me-L-Val-L-Val-*N*-Me-L-Val-*N*-Me-L-Val-*N*-Me-L-Val-L-Ile-*N*-Me-L-Val-*N*-Me-Gly-Abz. The sequence of the amino acid residues and their configurations of **52** were identified by the same methods as those of **51**, as well as by a comparison of the NMR spectra and HPLC retention time of its aglycone with those of **51**. The presence of D-fructose was confirmed by gas chromatography (GC) analysis using the chiral column (Chirasil-Val) of the tetramethyl silyl (TMS) derivative of the methanolysis product of **52** [56].

A chemical investigation of the EtOAc extract of the culture of an endophytic fungus, *Tolypocladium* sp., isolated from a marine microalga, *Spongomorpha arcta*, collected from the shores at Green’s Point, L’Etete, NB, Canada, furnished two undescribed lipopeptaibols, tolypocaibols A (**53**) and B (**54**) (Figure 8). Extensive 1D and 2D NMR analysis, including COSY and HMBC correlations, revealed the identity of the amino acid residues, the Aib and decanoyl (Dc) moiety, while the existence of a valinol residue was confirmed by ^1^H–^1^H TOCSY and COSY correlations. HMBC and ROESY correlations established the sequence of the amino acids in **53** as Dc-Aib^11^-Pro^10^-Phe^9^-Aib^8^-Gln^7^-Gln^6^-Aib^5^-Aib^4^-Gln^3^-Leu^2^-Valol^1^, which was supported by the MS/MS fragmentation data. By using the same methodology, it was found that the structure of **54** is very similar to that of **53** except that one Pro (Pro^10^) in **53** was replaced by a 4-methyl-proline (4-Me-Pro^10^) residue in **54**. Thus, the amino acid sequence in **54** was established as Dc-Aib^11–^4-Me-Pro^10^-Phe^9^-Aib^8^-Gln^7^-Gln^6^-Aib^5^-Aib^4^-Gln^3^-Leu^2^-Valol^1^ [57].

The absolute configurations of the amino acid and amino alcohol residues in **53** and **54** were determined by hydrolysis and the application of Marfey’s method which led to the determination of an L-configuration for all amino acids, and the stereochemistry of the 4-Me-Pro^10^ in **54** was 2*S*,4*S*. Therefore, the complete structures of **53** and **54** were determined as Dc-L-Aib^11^-L-Pro^10^-L-Phe^9^-L-Aib^8^-L-Gln^7^-L-Gln^6^-L-Aib^5^-L-Aib^4^-L-Gln^3^-L-Leu^2^-L-Valol^1^ and Dc-L-Aib^11^-(2*S*,4*S*)-4-Me-L-Pro^10^-L-Phe^9^-L-Aib^8^-L-Gln^7^-L-Gln^6^-L-Aib^5^-L-Aib^4^-L-Gln^3^-L-Leu^2^-L-Valol^1^, respectively [57].

#### 3.1.7. Linear Dodecapeptides

**Figure 9:**
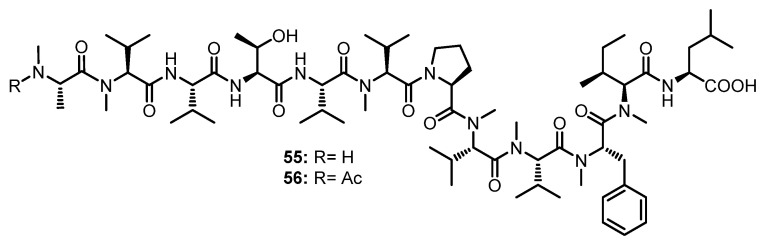
Structures of **55** and **56**.

Two *N*-methylated linear dodecapeptides, talaropeptides B (**55**) and D (**56**) (Figure 9), were also isolated, together with **49** and **50** (Figure 8), from the culture extract of *Talaromyces* sp. (CMB-TU011). The 1D and 2D NMR data revealed the presence of twelve amino acid residues in both **55** and **56**, *viz.* Thr, Pro, Val (x2), Leu, *N*-Me-Ala, *N*-Me-Val (x4), *N*-Me-Phe, and *N*-Me-Ile. Similar to **49** and **50**, the absolute configuration of all amino acids in **55** and **56** were determined as L by Marfey’s method. Diagnostic HMBC and ROESY correlations of **55** and **56** allowed us to assign the partial amino acid sequences; however, the connection of amino acids was established by diagnostic UHPLC-QTOF-MS/MS fragmentation patterns. A combination of the assembly of the fragments and HMBC correlations allowed us to elucidate the complete structures of **55** and **56** as *N*-Me-L-Ala^1^-*N*-Me-L-Val^2^-L-Val^3^-L-Thr^4^-L-Val*-*N*-Me-L-Val^5^-L-Pro^6^-*N*-Me-L-Val^7^-*N*-Me-L-Val^8^-*N*-Me-L-Phe^9^-*N*-Me-L-Ile^10^-L-Leu^11^, and *N*-Me-*N*-Ac-L-Ala^1^-*N*-Me-L-Val^2^-L-Val^3^-L-Thr^4^-L-Val*-*N*-Me-L-Val^5^-L-Pro^6^-*N*-Me-L-Val^7^-*N*-Me-L-Val^8^-*N*-Me-L-Phe^9^-*N*-Me-L-Ile^10^-L-Leu^11^, respectively [55].

#### 3.1.8. Linear Pentadecapeptides

**Figure 10:**
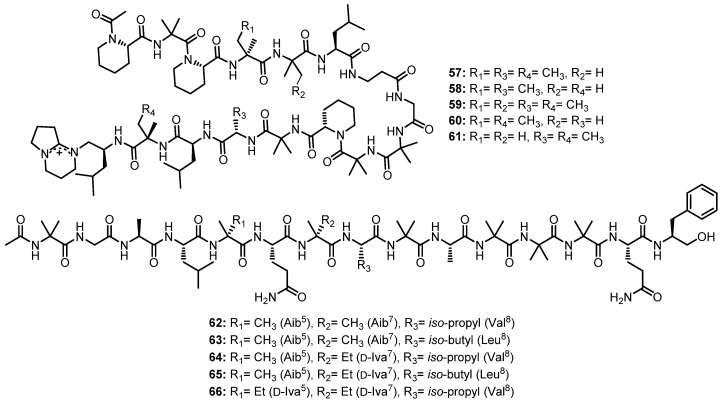
Structures of **57**–**66**.

A linear pentadecapeptide, efrapeptin G (**57**) (Figure 10), was also reported, together with the undescribed linear pentadecapeptides, efrapeptins Eα (**58**) and H (**59**) (Figure 10), and the previously reported efrapeptins E (**60**) and F (**61**) (Figure 10), from the culture extract of an atypical *Acremonium* sp. (UCSC coll. no. 021172 cKZ) [52]. The structures of **58** and **59** were established by extensive MS*^n^* fragmentations using a linear quadrupole ion trap electrospray ionization mass spectrometry (LTQ ESI-MS) technique while the absolute configurations of the amino acid residues in **58** and **59** were found to be the same as those of the previously discussed peptides of the RHM (**39**–**42**) and efrapeptin (**57**, **60** and **61**) families [53].

Van Bohemen et al., in their search for new peptaibols, described the isolation of five unreported 15-residue peptaibols, pentadecaibins I–V (**62**–**66**) (Figure 10) from the solid culture extract of a marine sediment-derived fungus, *Trichoderma* sp. MMS1255, belonging to the *T. harzianum* clade. The sequences of their amino acids were determined by the MS/MS fragmentation and extensive 1D and 2D NMR analysis as Ac-Aib^1^-Gly^2^-Ala^3^-Leu^4^-Aib^5^-Gln^6^-Aib^7^-Val^8^-Aib^9^-Ala^10^-Aib^11^-Aib^12^-Aib^13^-Gln^14^-Pheol^15^ for **62**, Ac-Aib^1^-Gly^2^-Ala^3^-Leu^4^-Aib^5^-Gln^6^-Aib^7^-Leu^8^-Aib^9^-Ala^10^-Aib^11^-Aib^12^-Aib^13^-Gln^14^-Pheol^15^ for **63**, Ac-Aib^1^-Gly^2^-Ala^3^-Leu^4^-Aib^5^-Gln^6^-Iva^7^-Val^8^-Aib^9^-Ala^10^-Aib^11^-Aib^12^-Aib^13^-Gln^14^-Pheol^15^ for **64**, Ac-Aib^1^-Gly^2^-Ala^3^-Leu^4^-Aib^5^-Gln^6^-Iva^7^-Leu^8^-Aib^9^-Ala^10^-Aib^11^-Aib^12^-Aib^13^-Gln^14^-Pheol^15^ for **65**, and Ac-Aib^1^-Gly^2^-Ala^3^-Leu^4^-Iva^5^-Gln^6^-Iva^7^-Val^8^-Aib^9^-Ala^10^-Aib^11^-Aib^12^-Aib^13^-Gln^14^-Pheol^15^ for **66** [58].

#### 3.1.9. Linear Octadecapeptides

**Figure 11:**
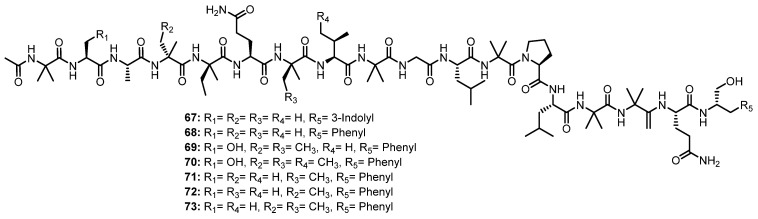
Structures of **67**–**73**.

The EtOAc extract of the solid rice culture broth of a marine-derived fungus, *Trichoderma* sp. GXIMD 01001, isolated from a marine sponge, *Haliclona* sp., which was collected in Beibu Gulf, Guangxi, China, furnished seven unreported 18-residue peptaibols, trichorzins A–G (**67**–**73**) (Figure 11) [59].

A detailed 1D and 2D NMR spectral analysis, together with high-resolution electrospray ionization mass spectrum (HRESIMS) data, confirmed the presence of 17 amino acid residues, including two Ala, six Aib, two Gln, one Gly, one Pro, two Leu, two Iva, and one Val, as well as an *N*-acetyl terminus and a Trpol (tryptophanol) at the C-terminus in **67**, while **68**–**73** contain the monosubstituted phenyl ring (Pheol) instead of indolyl (Trpol) at the C-terminus. The amino acid sequence was established by an examination of NOESY correlations from the NH proton at the acetyl terminus to the next NH and/or α-H of the adjacent amino acid residue, which ultimately gave a complete sequence of **67**–**73** as Ac-Aib^1^-Ala/Ser^2^-Ala^3^-Aib/Iva^4^-Iva^5^-Gln^6^-Aib/Iva^7^-Val/*allo*-Ile^8^-Aib^9^-Gly^10^-Leu^11^-Aib^12^-Pro^13^-Leu^14^-Aib^15^-Aib^16^-Gln^17^-Trpol/Pheol^18^ [59].

The absolute configurations of the stereogenic carbons of **68**–**73** were determined by Marfey’s method which indicated the presence of an L-configuration for Ala, Ser, Val, Leu, Pro, and Glu (resulting from the hydrolysis of Gln), as well as the characteristic nonproteinogenic peptaibol residues D-Iva, L-Trpol, and L-Pheol. However, the L-form of *allo*-Ile was observed for the Ile residue in **71**. Moreover, the ECD spectra of **67**–**73** showed a positive maximum near 200 nm and two negative maxima at 207 and 225 nm, suggesting, therefore, a right-handed helical conformation [59].

#### 3.1.10. Linear Lipopeptides

**Figure 12:**
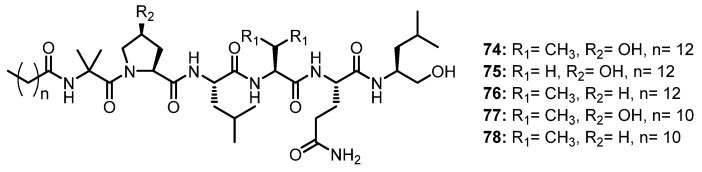
Structures of **74**–**78**.

The antiviral-activity-guided isolation of the saline fermentation extract of a marine- derived fungus, *Scytalidium* sp., led to the isolation of five undescribed lipophilic linear hexapeptides, halovirs A–E (**74**–**78**) (Figure 12), which contain an unusual Aib-hydroxyproline (OH-Pro) dipeptide fragment, while a carboxyl terminus is reduced to a primary alcohol. In order to determine the absolute stereochemistry, the acidic hydrolysis products of **74**–**78** were analyzed by GC with a flame ionization detector (FID) using a chiral column (Chirasil-Val), followed by Mosher’s method. L-Leu, L-Val, and L-Glu were identified in **74** and L-Ala, L-Leu, and L-Glu were identified in **75**, while L-Pro was found in **76**. In addition, the absolute configuration at C-3 of the 3-hydroxyproline (3-OH-Pro) residue in **74** was determined as 3*R* by Mosher’s method. Moreover, detailed 1D and 2D NMR spectral analyses, in combination with matrix-assisted laser desorption/ionization Fourier-transform-MS (MALDI-FTMS) data, confirmed that **77** and **78** derived from **74** and **76**, respectively [60].

#### 3.1.11. Other Linear Peptides

**Figure 13:**
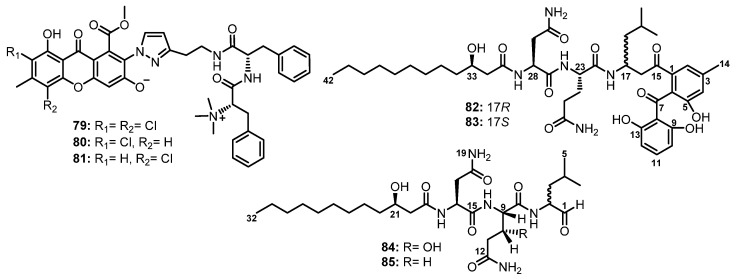
Structures of **79**–**85**.

Flavipesides A–C (**79**–**81**), unusual linear methylated dipeptides containing chlorinated xanthone and modified pyrazol residues (Figure 13), were isolated from the culture extract of *A. flavipes* 164013, obtained from a marine sponge *Dysidea* sp., which was collected off the Yongxing Island in the South China Sea. Although the structures of the subunits in **79**–**81** were clarified by 1D and 2D NMR correlations, the linkages between the substructures remained unsolved. The absolute configurations of two amino acids in **79** were determined as L, i.e., 23*S* and 32*S* by single-crystal X-ray analysis using Cu Kα radiation. For **80** and **81**, the absolute configurations of the two amino acids were found to be the same as those of **79** since their specific rotations (${\left[\alpha \right]}_{D}$ −14.7 for **80**, and −15.6 for **81)** were similar to that of **79** (${\left[\alpha \right]}_{D}$ −11.5). Moreover, the cotton effects in the experimental ECD spectra of **80** and **81** were well-matched with that of **79**. Compounds **79**–**81**, possessing a *N*,*N*,*N*-trimethyl group to form an ammonium salt of Phe, are not common for natural products [61].

Two epimeric lipopeptidyl benzophenones, asperphenins A (**82**) and B (**83**) (Figure 13), were isolated from a culture broth extract of a marine-derived fungus, *Aspergillus* sp., collected from a marine-submerged decaying wood. Detailed NMR analysis revealed that the structures of **82** and **83** comprise three different motifs, including a hydroxy fatty acid, a tripeptide, and a trihydroxybenzophenone. Interestingly, the absolute configuration of the hydroxyl-bearing carbon, C-33, was determined as *R* by Mosher’s NMR method while the absolute configurations of the stereogenic centers, C-23 and C-28, in **82** and **83** were determined as L by Marfey’s analysis [62].

The culture extract of a marine fungus, *Penicillium fellutanum* Biourge, isolated from the gastrointestine of a marine fish, *Apogon endekataenta* Bleeker, furnished two undescribed linear cytotoxic lipopeptides, fellutamides A (**84**) and B (**85**) (Figure 13), which contain 3-hydroxydodecanoic acid (HDA), β-*threo*-hydroxy gluthamine (βHGln), and 2-amino-4-methylpentanal (leucinal). Detailed HMBC correlations, FAB-MS/MS, and chiral GC and HPLC analyses determined the sequences of the amino acids and their absolute stereochemistry. Therefore, **84** was elucidated as HDA-L-Asn-L-*threo*-βHGln-Leucinal, while **85** was identified as HDA-L-Asn-L-Gln-Leucinal. The absolute configuration at C-3 in HDA was determined as *R* by the sign of its optical rotation $({\left[\alpha \right]}_{D}^{27}$ −17) [63].

### 3.2. Cyclic Peptides

Cyclic peptides containing 2 to 10 amino acid residues are biosynthesized by NRPS.

#### 3.2.1. Cyclic Dipeptides

**Figure 14:**
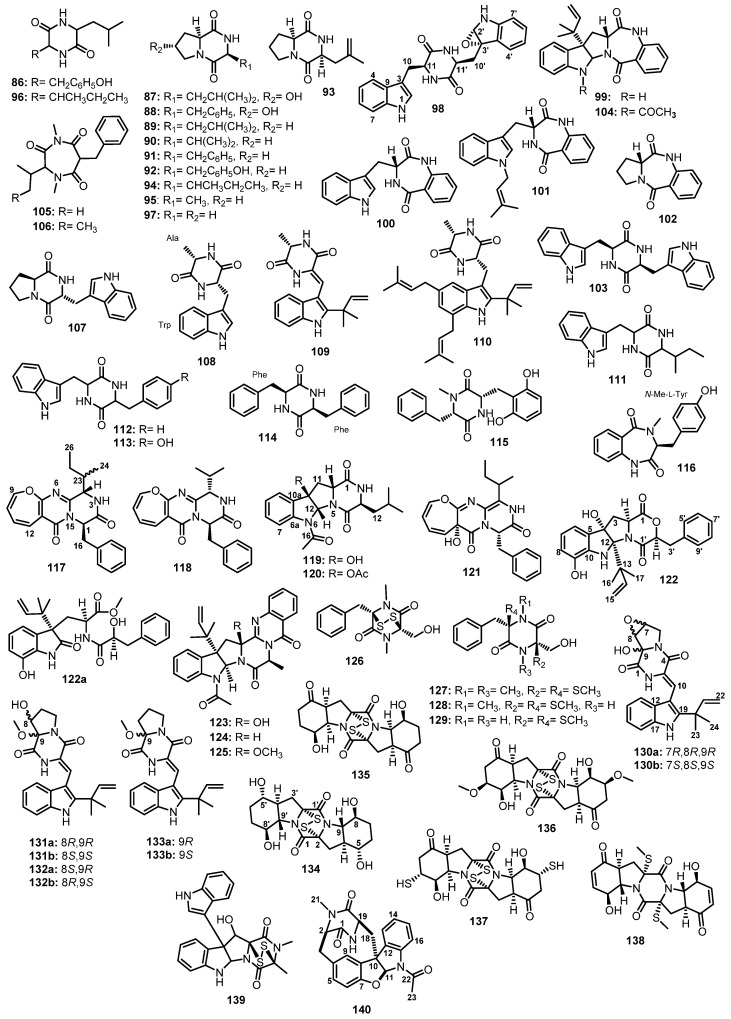
Structures of **86**–**140**.

Scopel et al., in their search for novel compounds against anti-biofilm formation, reported the isolation of a cyclic dipeptide, *cis*-*cyclo-*(Leu-Tyr) (**86**) (Figure 14), from *Penicillium* sp. F37, which was obtained from a marine sponge, *Axinella corrugata* [64].

*Cyclo*-(*trans*-4-hydroxy-L-Pro-L-Leu) (**87**), *cyclo*-(*trans*-4-hydroxy-L-Pro-L-Phe) (**88**), *cyclo*-(L-Pro-L-Leu) (**89** or 3-isobutylpyrrolopiperazine-1,4-dione), *cyclo*-(L-Pro-L-Val) (**90**), *cyclo*-(L-Pro-L-Phe) (**91**), and *cyclo*-(L-Pro-L-Tyr) (**92**) (Figure 14) were isolated from the culture extract of a marine sediment-derived fungus, *A. niger* BRF-074, collected from the Northeast Brazilian coast [65]. Compounds **89** and **92** were also isolated from the fermentation extracts of two Taiwanese sediment-derived fungi, *Fusarium* sp. RWS56-10 [66] and *P. chrysogenum* DXY-1, respectively [67].

Penicimutide (**93**), which contains an unusual amino acid, 4,5-didehydro-L-leu, and *cyclo*-(L-Ile-L-Pro) (**94**) (Figure 14) were isolated, together with **89**–**91** (Figure 14), from a neomycin-resistant mutant of a marine sediment-derived fungus, *P. purpurogenum* G59, collected at the tideland of Bohai Bay around Lujuhe in the Tanggu District of Tianjin, China [68].

A chemical investigation of a marine-derived fungus, *Ascotricha* sp. ZJ-M-5, furnished *cyclo*-(Pro-Ala) (**95**), *cyclo*-(Ile-Leu) (**96**), and *cyclo*-(Gly-Pro) (**97**) (Figure 14), in addition to **89** and **90** [69].

The EtOAc extract of a culture of *Neosartorya glabra* KUFA 0702, isolated from a marine sponge *Mycale* sp., collected at a depth of 15–20 m from the coral reef at Samaesarn Island in the Gulf of Thailand, furnished an unreported fellutanine A epoxide (**98**), in addition to aszonalenin (**99**), (3*R*)-3-(1*H*-indol-3-ylmethyl)-3,4-dihydro-1*H*-1,4-benzodiazepine-2,5-dione (**100**), takakiamide (**101**), (11a*R*)-2,3-dihydro-1*H*-pyrrolo[2,1-*c*][1,4] benzodiazepine-5,11(10*H*,11a*H*)-dione (**102**), and fellutanine A (**103**) (Figure 14). In the case of **98**, the absolute configurations of the stereogenic carbons of the diketopiperazine ring (C-11 and C-11′) were proposed to be the same as those of the co-isolated **103**, i.e., 11*S*,11′*S* due to the same biosynthetic precursor (L-Trp). The NOESY experiments and molecular dynamic simulations not only confirmed the 11*S*,11′*S* configurations of the diketopiperazine but also suggested the stereochemistry of the epoxide ring as 2′*S*,3′*S* [70].

Capon et al. isolated acetylaszonalenin (**104**) (Figure 14), a known terrestrial fungal metabolite, from the mycelial extract of an estuarine sediment-derived fungus, *A. carneus* (MST-MF156), collected in Tasmania, Australia [71]. May Zin et al. also reported the isolation of **97**, **101**, and **104** (Figure 14) from the EtOAc extract of a solid rice culture of an algicolous fungus, *N. takakii* KUFC 7898, collected from Samaesarn Island in the Gulf of Thailand [72].

The mycelium extract of a marine sponge-associated fungus, *A. terreus* SCSIO41008, isolated from the marine sponge, *Callyspongia* sp., which was collected from the seaside in Guangdong Province, China, yielded terretriones B (**105**) and C (**106**), and brevianamide F (**107**), together with **91** (Figure 14) [42].

A chemical investigation of the solid culture extract of *Eurotium chevalieri* MUT2316, isolated from the Atlantic sponge, *Grantia compressa*, led to the isolation of *cyclo*-(L-Trp-L-Ala) (**108**), together with two prenylated cyclic dipeptides, neoechinulin A (**109**) and echinulin (**110**) (Figure 14) [73]. In another study, **109** was also reported from a marine-derived fungus, *Microsporum* sp. strain MFS-YL, which was isolated from the surface of a marine red alga, *Lomentaria catenata*, collected at Guryongpo, Nam-Gu, PoHang in the Republic of Korea [74]. Smetanina et al. also reported the isolation of **110** from the mycelium extract of a marine-derived *E. repens*, isolated from a marine sponge, *Suberites domuncula*, collected near Zelenyi Island [75].

Zhang et al. reported the isolation of three cyclodipeptides, *cyclo*-(L-Trp-L-Ile) (**111**), *cyclo*-(L-Trp-L-Phe) (**112**), and *cyclo*-(L-Trp-L-Tyr) (**113**) (Figure 14), from the culture broth extract of *A. niger* EN-13, which was obtained from the inner tissue of a marine brown alga, *Colpomenia sinuosa*, collected from the Qingdao coastline, China [76].

Buttachon et al. reported the isolation of (3*S*,6*S*)-3,6-dibenzylpiperazine-2,5-dione (**114**) (Figure 14) from the culture extract of *A. candidus* KUFA0062, obtained from a marine sponge *Epipolasis* sp., which was collected from the coral reef at the Similan Island National Park, Phang-Nga province, Thailand [77].

A chemical investigation of the EtOAc extract of the fermentation of *A. ochraceopetaliformis* DSW-2, which was obtained from the sea water sample from Dongshan Island in Fujian Province of China, led to the isolation of mactanamide (**115**), in addition to **99** (Figure 14) [78].

A deep-sea sediment-derived fungus, *Aspergillus* sp. SCSIOW2, collected in the South China Sea, furnished an undescribed cyclic dipeptide, 14-hydroxy-cyclopeptine (**116**) (Figure 14). The ^1^H NMR spectrum of **116** in DMSO-*d*_6_, at room temperature, displayed two sets of proton signals. However, the two sets of signals began to merge when the temperature was raised to 50 °C and coalesced into single resonances at 85 °C, indicating the presence of two different stable conformational isomers of **116**. This hypothesis was further confirmed by ROESY correlations. An extensive 1D and 2D NMR analysis revealed the presence of *N*-Me-Tyr and Abz. The L-configuration of *N*-Me-Tyr was established by Marfey’s method [79].

The oxepin-containing pyrazinopyrimidin-7-one derivatives, protuboxepins A (**117**) and B (**118**), and the pyrroloindolodiketopiperazine derivatives, protubonines A (**119**) and B (**120**) (Figure 14), were obtained from the EtOAc extract of a culture of a marine-derived fungus, *Aspergillus* sp. SF-50044, isolated from the intertidal sediment sample collected from Dadaepo Beach, Busan, Korea. Then, 1D and 2D NMR analysis revealed that **117** contained Leu and Phe residues, while **118** consisted of Val and Phe. Marfey’s method was used to identify the configuration of D-Phe in **117**. Since the NOESY spectrum showed correlations from H-4 to H_2_-16 and H-18/H-22, these protons are cofacial. Therefore, the absolute configuration at C-1 and C-4 were determined as *R* and *S*, respectively, while the configuration at C-23 remained unassigned. For **119** and **120**, an analysis of the COSY and HMBC correlations confirmed the presence of Leu and the diketopiperazine ring. Through a combination of NOESY correlations with the result of Marfey’s analysis, L-Leu was identified. Thus, the absolute configurations of the stereogenic carbons in **119** and **120** were determined as 3*S*,5a*R*,10b*R*,11a*R* [80].

Oxepinamide E (**121**) (Figure 14), an oxepin-containing pyrimidine alkaloid, was isolated together with **117**, from the mycelial extract of a co-fermentation of the strains BM-05 and BM-05ML of *Aspergillus* sp., isolated from a marine brown alga *Sargassum* sp., which was collected off Helgoland, North Sea, Germany [81].

The EtOAc culture extract of a marine sediment-derived fungus, *Aspergillus* sp. (CMB-M081F), collected from the intertidal depth of 1 m near Shorncliffe, Queensland, Australia, yielded an undescribed shornephine A (**122**) and its methanolysis artifact of a solvolytically unstable product, *seco*-shornephine A methyl ester (**122a**), together with the previously described 15b-*β*-hydroxy-5-*N*-acetyladreemin (**123**), 5-*N*-acetyladreemin (**124**), and 15b-*β*-methoxy-5-*N*-acetyladreemin (**125**) (Figure 14). Detailed 2D NMR analysis, including COSY and HMBC correlations, established the planar structures of **122** and **122a**. Further diagnostic of the ROESY correlations revealed that H-2, H-2′, H-3α, 4-OH, H_3_-16, and H_3_-17 are on the α face, thus establishing the relative configuration of **122** [82].

The EtOAc broth extract of *Asteromyces cruciatus* 763, obtained from an unidentified decaying green alga which was collected at La Jolla shore, San Diego, USA, yielded hyalodendrin (**126**), gliovictin (**127**), ^1^*N*-norgliovictin (**128**), and bis-*N*-norgliovictin (**129**) (Figure 14), and four previously described (3*R*,6*R*)-epipolythiopiperazinedione antibiotics [83].

The culture extract of a marine sediment-derived fungus, *A. versicolor* MF180151, which was collected from the Bohai Sea, China, furnished diketopiperazine derivatives, including (±)-7,8-epoxy-brevianamide Q ((±)-**130**), (±)-8-hydroxybrevianamide R ((±)-**131**), (±)-8-epihydroxybrevianamide R ((±)-**132**), and (±)-brevianamide R ((±)-**133**) (Figure 14). The planar structures of **130**–**132** were established by 1D and 2D NMR spectral analysis. ROESY correlations between 2-NH and H-13 confirmed the presence of a *cis* double bond between C-3 and C-10 in **130**–**132**. Further ROESY correlations from OH-9 to H-7 and H-8 in **130**, and from OCH_3_-9 to H-8 in **131**, revealed their relative configurations. A comparison of the chemical shift values of C-9 in **131** and in **132** suggested that **132** was an epimer of **131**. Circular dichroism (CD) experiments of **130**–**132** did not exhibit a significant absorption. However, the values of optical rotations, ${\left[\alpha \right]}_{D}^{25}$ +3.0 (*c* 0.1, CH_3_OH) for (±)-**130**, ${\left[\alpha \right]}_{D}^{25}$+1.0 (*c* 0.1, CH_3_OH) for (±)-**131,** and ${\left[\alpha \right]}_{D}^{25}$ +2.0 (*c* 0.1, CH_3_OH) for (±)-**132**, were inherent to the racemic nature of **130**–**132** [84].

The cytotoxic-activity-guided fractionation of the culture broth extract of *Exserohilum rostratum* (Drechsler) CNK-630, obtained from a marine cyanobacterial mat which was collected off the northwest corner of Lanai Island, Hawaii, furnished four unreported *C_2_*-symmetrical diketopiperazine disulfides, rostratins A–D (**134**–**137**) (Figure 14), together with exserohilone (**138**) (Figure 14). The symmetrical nature of **134**–**137** were determined by analyses of their 1D and 2D NMR spectra in combination with their molecular formulae. A comparison of the 1D and 2D NMR data of **134**–**137** with those of **138** revealed that **134**–**137** are formed by the cyclic dimerization of highly oxidized Phe residues. The absolute configurations of the stereogenic carbons in **134**–**137** were determined by a combination of NOESY correlations and regioselective acylation by Mosher’s reagents in the NMR tube at reduced temperatures. As a result, the absolute structures of **134**–**137** were elucidated as 2(2′)*R*,4(4′)*S*,5(5′)*S*,8(8′)*S*,9(9′)*S*-**134**, 2(2′)*R*,4(4′)*S*,8(8′)*S*,9(9′)*R*-**135**, 2(2′)*R*,4(4′)*S*,7(7′)*S*,8(8′)*R*,9(9′)*R*-**136**, and 2(2′)*R*,4(4′)*S*,7(7′)*R*,8(8′)*R*,9(9′)*R*-**137** [85].

Following the bioassay-guided isolation approach, gliocladine C (**139**) (Figure 14) was isolated, together with **91**, **97**, and **108**, from a fermentation broth extract of a marine sediment-derived fungus, *Penicillium* sp. WF-06 [86].

**Figure 15:**
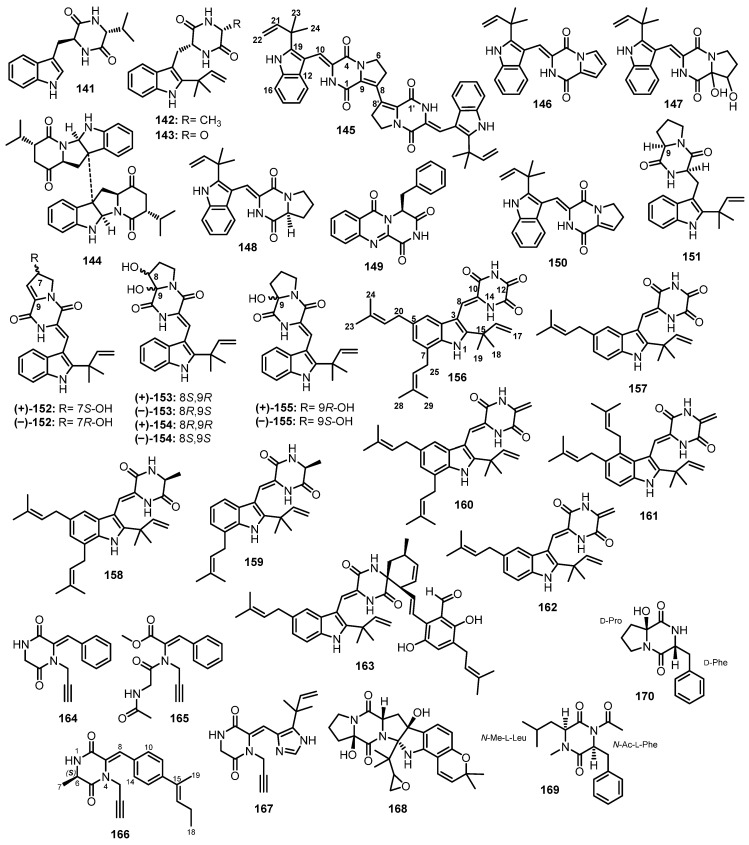
Structures of **141**–**170**.

Wu et al., in their OSMAC (*O*ne *S*train *MA*ny *C*ompounds) program, reported the isolation of a structurally unique hexacyclic dipeptide, azonazine (**140**) (Figure 14), from a Hawaiian marine sediment-associated fungus, *A. insulicola*. The structure of **140** was assembled by an analysis of the HRESI-MS, gCOSY, gHMBC, and NOE correlations. The absolute configurations of the stereogenic carbons in **140** were determined as 2*R*,10*R*,11*S*,19*R* by comparison of its calculated and experimental ECD spectra [87].

(11*R*,14*S*)-3-(1*H*-indol-3-ylmethyl)-6-isopropyl-2,5-piperazinedione (**141**), preechinulin (**142**), neoechinulin E (**143**), eurocristatine (**144**) (Figure 15), and echinulin (**110**) (Figure 14) were isolated from a mycelial extract of a mangrove endophytic fungus, *E. chevalieri* KUFA 0006, which was obtained from the healthy twig of a mangrove tree, *Rhizophora mucronata* Poir., collected at the Eastern part of Thailand [88].

A chemical investigation of the EtOAc soluble fraction of the crude extract of a marine sediment-derived fungus, *A. versicolor* (MF030), isolated from the Bohai Sea, China, furnished an unreported dimeric diketopiperazine, brevianamide S (**145**), three undescribed diketopiperazines, brevianamides T (**146**), U (**147**), and V (**148**), together with the previously reported brevianamides N (**149**) and K (**150**), and deoxybrevianamide E (**151**) (Figure 15). An analysis of the (−)-HRESI-MS, ^1^H and ^13^C NMR data of **145** revealed its dimeric and symmetrical nature. An analysis of COSY, HMBC, and ROESY correlations established the planar structure of **145**. The ^1^H and ^13^C NMR spectra of **146**–**148** resembled those of **145**; however, their (−)-HRESI-MS data revealed that they were monomers. The 1D and 2D NMR data showed that the structure of **146** is similar to the monomer of **145** but with a double bond between C-6 and C-7, while **148** contains Pro residue, i.e., with no double bond between C-8 and C-9. Marfey’s analysis revealed the presence of L-Pro in **148**. On the other hand, **147** has two hydroxyl groups at C-8 and C-9 instead of a double bond; however, the absolute configurations at C-8 and C-9 in **147** were not determined [89].

Further diketopiperazines derived from a condensation of Pro and Trp, i.e., pairs of enantiomers of brevianamides Z ((±)-**152**) and Z1 ((±)-**153**), together with their analogues *viz.* brevianamides X ((±)-**154**), R ((±)-**133**), Q ((±)-**155**), K (**150**), V ((+)-**148**), and deoxy brevianamide E ((±)-**151**) (Figure 15), were isolated from the EtOAc extract of a culture of *A. versicolor* HBU-7, which was obtained from a sea mud sample collected from the coast of Bohai, China. The planar structures of (±)-**152** and (±)-**153** were elucidated by 1D and 2D NMR spectral analysis, especially COSY and HMBC correlations. The absolute configurations of the stereogenic centers in (+)-**152** and (−)-**152** were determined as 7*S* and 7*R*, respectively, by a comparison of their calculated and experimental ECD spectra. On the other hand, the absolute configurations of the stereogenic centers in (+)-**153**, (−)-**153**, (+)-**154**, and (−)-**154** were determined using DP4+ data analysis of the unscaled shift and shielding tensor data. The absolute configurations of (+)-**153** and (−)-**153** were established as 8*S*,9*R* and 8*R*,9*S,* respectively, while the absolute configurations of (+)-**154**, and (−)-**154** were determined as 8*R*,9*R* and 8*S*,9*S*, respectively [90].

An undescribed dioxopiperazine, 12-demethyl-12-*oxo*-eurotechinulin B (**156**), was isolated, together with previously reported variecolorin J (**157**), eurotechinulin B (**158**), variecolorin G (**159**), alkaloid E-7 (**160**), cryptoechinuline G (**161**), isoechinulin B (**162**), and 7-isopentenylcryptoechinuline D (**163**) (Figure 15), from a solid-rice culture extract of a mangrove endophytic fungus, *E. rubrum* G2, which was obtained from the inner tissue of the semi-mangrove plant, *Hibiscus tiliaceus* Linn., collected from Hainan Island, China [91].

By using an MS/MS-based molecular networking approach, Mao et al. isolated three unreported *N*-ethynyl diketopiperazine derivatives, sclerotioloid A (**164**), its *seco*-analog, sclerotioloids B (**165**), and sclerotioloids C (**166**), together with two previously described ones, gartryprostatin C (**167**) and speramide C (**168**) (Figure 15), from the EtOAc fermentation extract of *A. sclerotiorum* ST0501, isolated from the inner tissue of an unidentified marine sponge which was collected from the South China Sea, Guangdong, China. The structures of **164**–**166** were elucidated by the interpretation of 1D and 2D spectra. In the case of **165**, its structure and the configuration of the double bond were confirmed by X-ray analysis. The absolute configuration of the sterogenic carbon, C-6, in **166** was established as 6*S* by comparison of its calculated and experimental ECD spectra [92].

The cytotoxic fraction of the EtOAc fermentation broth extract of *Aspergillus* sp. DY001, isolated from the internal tissue of a tunicate, *Didemnum* sp., collected off Jizan, at the Saudi Red Sea coast, furnished two undescribed cyclic dipeptides, asperopiperazines A (**169**) and B (**170**) (Figure 15). Detailed 1D and 2D NMR spectral analysis revealed the presence of disubstituted diketopiperazine which is derived from *N*-Me-Leu and *N*-Ac-Phe in **169**. The absolute configurations of Leu and Phe were established as L by Marfey’s analysis. Therefore, the complete structure of **169** was elucidated as *cyclo*-(*N-*Me-L-Leu-N-Ac-L-Phe. On the other hand, the 1D and 2D NMR spectral analysis showed that **170** is also a trisubstituted diketopiperazine. An analysis of the structure of **170** revealed that it consists of 2-hydroxy Pro (2-OH-Pro) and Phe. The absolute configuration of Phe was determined as D-Phe by Marfey’s method while the absolute configuration of C-6 (of the Pro residue) was established as D-Pro by a comparison of the ^13^C NMR chemical shift of C-6 with the corresponding carbon of the previously described *cyclo*-(6-OH-D-Pro-L-Phe) and *cyclo*-(6-OH-L-Pro-L-Phe). Therefore, the structure of **170** was established as *cyclo*-(6-OH-D-Pro-L-Phe) [93].

**Figure 16:**
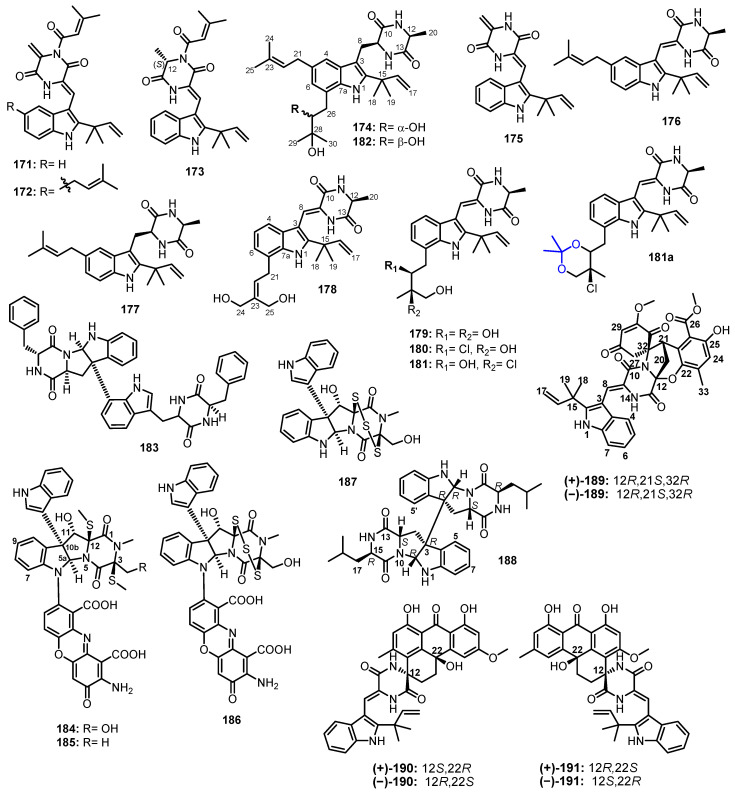
Structures of **171**–**191**.

A hemical investigation of the methanol extract of a slid rice culture of *Aspergillus* sp. FS445, isolated from a deep-sea sediment which was collected from the Indian Ocean, furnished four unreported cyclic dipeptides, aspechinulins A–D (**171**–**174**), together with eight known cyclic dipeptides, including neoechinulin B (**175**), isoechinulin A (**176**), tardioxopiperazine A (**177**) (Figure 16), **109**, **110** (Figure 14), and **160**–**162** (Figure 15). Detailed analysis of 1D and 2D NMR spectra of **171**–**174** showed that they are prenylated indole diketopiperazine derivatives comprising of Trp and Ala. The *cis* configuration of the double bond between C-8 and C-9 in **171**–**173** was established based on the NOESY correlation between H-4 and NH-14. The absolute configuration of C-12 in **173** was established as 12*S*, based on the negative value of its optical rotation and a similarity of its ECD spectrum and the ECD spectrum of rubrumazines A/B (negative Cotton effect at 205 nm and positive Cotton effects at 230 nm) [94]. This was confirmed by a comparison of the calculated and experimental ECD spectra of **173** [95].

For **174**, which showed a negative value of rotation similar to that of the previously described rubrumazine C, its H-9 and H-12 were deduced to be co-facial. Moreover, since the experimental ECD spectrum of **174** displayed a negative Cotton effect at 230 nm, the configuration at C-9 was suggested to be 9*S*, which was the same as that of rubrumazine C [93]. Thus, the absolute configurations of C-9 and C-12 in **174** were assigned to be 9*S*,12*S*. On the other hand, the absolute configuration of C-27 could not be assigned either by Mosher’s method or ^13^C calculations. Moreover, the calculated ECD spectrum exhibited mirrored Cotton effects which made the authors speculated that a hydrogen bond between the hydroxyl group at C-27 and NH-1, which formed a seven-membered ring, could influence the Cotton effect in the ECD spectrum. By a comparison of the calculated and experimental ECD spectra of the analogs of **174**, prepared by replacing the hydroxyl group at C-27 by the methyl group, the authors were able to establish the absolute configuration of C-27 as 27*R* [95].

The EtOAc extract of a solid rice culture of *A. chevalieri* CS-122, isolated from a deep-sea cold seep sediment collected in the northeast of the South China Sea, furnished five unreported cyclic dipeptides, 24,25-dihydroxyvariecolorin G (**178**), 25-hydroxyrubrumazine B (**179**), 22-chloro-25-hydroxyrubrumazine B (**180**), 25-hydroxyvariecolorin F (**181**), and 27-*epi*-aspechinulin D (**182**), together with the known analogue, neoechinulin B (**175**) (Figure 16). Detailed analysis of 1D and 2D NMR spectral and (+)-HRESI-MS data revealed that **178**–**182** are also prenylated indole diketopiperazine derivatives, comprising prenylated Trp and Ala. The *Z* geometry of the double bond at C-8 was established based on the downfield chemical shift of H-8 caused by the deshielding effect of the carbonyl group. The absolute configuration of Ala was determined as L by chiral HPLC of the hydrolysis products of **178**–**182** [96].

The relative configurations of C-22 and C-23 in **179**–**182** were established by a comparison of the experimental ^1^H and ^13^C chemical shift values with those obtained from GIAO (gauge-including atomic orbitals) NMR calculations at the mPW1PW91/6-31+G(d,p) level with DP4+ probability analyses. The results showed that the ^1^H and ^13^C chemical shift values of **178** and **182** matched well with the 12S*,22S*,23R* configurations. Moreover, the relative configurations obtained by an analysis of the ^13^C chemical shift values and NOESY correlations of the key protons of the acetonide of **182** were in agreement with those obtained from DP4+ probability analysis. Thus, the absolute configurations of C-22 and C-23 were tentatively assigned as 22*S*,23*R* by correlating with the absolute configuration of C-12 which was unambiguously established as 12*S* [96].

A chemical investigation of the CH_2_Cl_2_ fraction of the EtOAc extract of a culture of *A. niger*, isolated from a marine sponge *Hyrtios proteus*, which was collected in the Dry Tortugas National Park, Florida, yielded a previously reported dimeric cyclo dipeptide, asperazine (**183**) (Figure 16) [97].

The in vitro screen for the indoleamine 2,3-dioxygenase (IDO) inhibition of the crude extract of the solid culture of a marine sediment-derived fungus, *Plectosphaerella cucumerina*, collected at −100 m depth in Barkley Sound, British Columbia, led to the isolation of the undescribed plectosphaeroic acids A–C (**184**–**186**), together with the previously reported T988 A (**187**) (Figure 16). The planar structures of **184**–**186** were established by extensive analysis of 1D and 2D NMR and (−)-HRESI-MS data. Compounds **184** and **185** consist of a bis-(methylthio)-diketopiperazine fused with 3-methyl-2,3-dihydro-1*H*-indole, while **186** contains a trisulfide bridge across the diketopiperazine ring. Biogenetically, the indolylmethyldiketopiperazine core in **184** and **186** is formed by the condensation of Trp and Ser, while that of **185** is formed from Trp and Ala. Compounds **184**–**186** also featured an indole and a phenoxazinone substituents. The absolute configurations of the stereogenic carbons in **184**–**187** were determined as 3*S*,5a*R*,10b*R*,11*S*,12*S* by a comparison of their CD spectra with the literature values for structurally related leptosins [98].

The EtOAc extract of a solid rice culture of *A. violaceofuscus,* which was isolated from the inner part of a marine sponge *Reniochalina* sp., collected from the Xisha Islands in the South China Sea, furnished an undescribed indolylmethyl diketopiperazine dimer (**188**) (Figure 16). The relative configuration of **188** was assigned by diagnostic NOESY correlations. The absolute configuration of the stereocenters in **188** were determined as 2*R*,3*R*,11*S*,15*R* by Marfey’s method [99].

Three pairs of racemic spirocyclic diketopiperazine enantiomers containing anthrone moiety, variecolortins A–C (**189**–**191**) (Figure 16), were isolated from a marine sediment-derived fungus, *Eurotium* sp. SCSIO F452, obtained from the South China Sea. While **189** possesses a highly functionalized *seco*-anthronopyranoid carbon skeleton featuring a 2-oxa-7-aza-bicyclo[3.2.1]octane moiety, **190** and **191** contain a 6/6/6/6 tetracyclic cyclohexene–anthrone moiety. The planar structures of **189**–**191** were established by extensive 1D and 2D NMR spectral analysis. In the case of **189**, the X-ray analysis not only established the relative stereochemistry as 12R*,21S*,32R*, but also indicated its racemic nature from the P21/n space group, which was supported by the lack of optical activity. The separation of a racemic **189** by chiral HPLC led to the obtention of pure (+)-**189** and (−)-**189**. The stereostructures of both enantiomers were determined by a comparison of their calculated and experimental ECD spectra, establishing (12*R*,21*S*,32*R*)-**189** for (+)-**189**. On the other hand, the racemic nature of **190** and **191** was deduced from their baseline ECD curves, as well as barely measurable optical rotations. The separation of racemic **190** and **191** by chiral HPLC resulted in the obtention of pure (−)-**190** and (+)-**190**, as well as pure (−)-**191** and (+)-**191**. A comparison of the calculated and experimental ECD spectra established the absolute structure of (+)-**190** as 12*S*,22*R*-**190** and of (−)-**191** as 12*S*,22*R*-**191** [100].

#### 3.2.2. Cyclic Tripeptides

**Figure 17:**
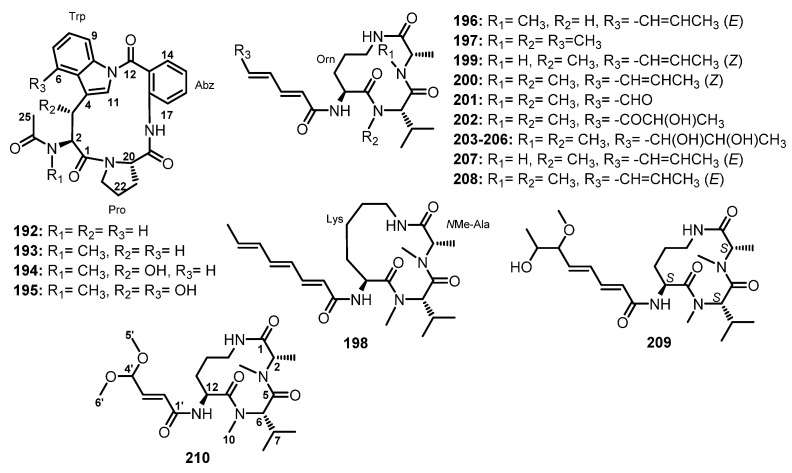
Structures of **192**–**210**.

Chemical investigation of the extract of a co-fermentation of the strains BM-05 and BM-05ML of *Aspergillus* sp., an endophytic fungus isolated from a marine brown alga *Sargassum* sp., also furnished a cyclic tripeptide, psychrophilin E (**192**) (Figure 17). Detailed analysis of 1D and 2D NMR spectra and HRESI-MS established the structure of **192** as *cyclo*-(*N*-Ac-Trp-Pro-Abz). A chiral-phased gas chromatographic (GC) analysis of methyl *N*-(trifluoroacetyl)-Pro derivative, obtained from the hydrolysis of **192**, led to the identification of L-Pro, thus establishing the absolute configuration of C-20 as 20*S*. However, the absolute configuration at C-2 could not be determined due to the decomposition of *N*-Ac-Trp during acid hydrolysis [81].

At the same time, Peng et al. isolated cyclic tripeptides, psychrophilins F–H (**193**–**195**) (Figure 17), in addition to **192**, from the solid rice culture extract of a marine mud-associated fungus, *A. versicolor* ZLN-60, by using the OSMAC approach. Interestingly, the authors were able to obtain a suitable crystal of **192** for X-ray analysis using Cu Kα radiation and established the absolute configurations of C-2 and C-20 as 2*S*,20*S*. For **193**, L-Pro was identified by advanced Marfey’s method, thus establishing the absolute configuration of C-20 as 20*S*. The configuration of C-2 in **193** is the same as that of **192** since they have similar CD curves. The absolute configurations of C-2, C-3, and C-20 of both **194** and **195** were established as 2*S*,3*R*,20*S* by a comparison of the calculated and experimental ECD spectra [101].

Purification of the fermentation broth extract of a halotolerant fungus, *A. sclerotiorum* PT06-1, which was isolated from the salt sediments from the Putian Sea Salt Field, Fujian, China, led to the isolation of undescribed cyclic aspochracin-type tripeptides containing an unsaturated fatty acid side chain, sclerotiotides A–K (**196**–**206**) (Figure 17), together with two previously reported tripeptides, JBIR-15 (**207**) and aspochracin (**208**) (Figure 17) [99].

The chemical transformation of **207** and **208** revealed that **199**–**206** were artifacts, probably formed during the fermentation process or subsequent isolation steps. The amino acid sequences of the compounds were established by HMBC correlations, while the absolute configurations of the amino acid residues were determined by Marfey’s method as (2*E*,4*E*,6*E*)-*cyclo*-[(*N*-Me-L-Ala)-L-Val-(*N*α-octa-2,4,6-trienoyl-L-Orn)] (**196**), (2*E*,4*E*)-*cyclo*-[(*N*-Me-L-Ala)-(*N*-Me-L-Val)-(*N*α-hexa-2,4-dienoyl-L-Orn)] (**197**), (2*E*,4*E*,6*E*)-*cyclo*-[(*N*-Me-L-Ala)-(*N*-Me-L-Val)-(*N*α-octa-2,4,6-trienoyl-L-Lys)] (**198**), (2*E*,4*E*,6*Z*)-*cyclo*-[L-Ala-(*N*-Me-L-Val)-(*N*α-octa-2,4,6-trienoyl-L-Orn)] (**199**), (2*E*,4*E*,6*Z*)-*cyclo*-[(*N*-Me-L-Ala)-(*N*-Me-L-Val)-(*N*α-octa-2,4,6-trienoyl-L-Orn)] (**200**), (2*E*,4*E*)-*cyclo*-[(*N*-Me-L-Ala)-(*N*-Me-L-Val)-(*N*α-6-oxohexa-2,4-dienoyl-L-Orn)] (**201**), and (2*E*,4*E*)-*cyclo*-[(*N*-Me-L-Ala)-(*N*-Me-L-Val)-(*N*α-7-hydroxy-6-oxoocta-2,4-dienoyl-L-Orn)] (**202**) [102].

Compounds **203**–**206** were stereoisomers. Since the NMR data of **203** and **204** were identical to those of **205** and **206**, respectively, **203** and **204**, and **205** and **206** were suggested to be enantiotopic in the fatty acid moiety. Since the ^3^*J*_(H6′,H7′)_ of **203**–**206** were small, all four compounds displayed a *gauche* conformation in the side chain. Moreover, **203**–**206** were produced by air oxidation of **208.** Therefore, the structures of **203** and **205**, and **204** and **206**, were determined as *threo*- and *erythro*-(2*E*,4*E*)-*cyclo*-[(*N*-Me-L-Ala)-(*N*-Me-L-Val)-(*N*α-6,7-dihydroxyocta-2,4-dienoyl-L-Orn)], respectively [102].

Sclerotiotide L (**209**) (Figure 17), another aspochracin-type cyclic tripeptide, was isolated from the EtOAc extract of a solid rice culture of *A. violaceofuscus*, obtained from the marine sponge *Reniochalina* sp., which was collected from the Xisha Islands in the South China Sea. The structure of **209** was established as (2′*E*,4′*E*)-*cyclo*-[(*N-*Me-L-Ala)-(*N-*Me-L-Val)-*N*α-6′-methoxy-7′-hydroxyocta-2′,4′-dienoyl-L-Orn)] by NMR spectral analysis and Mafrey’s method [99].

The EtOAc extract of a fermentation of *A. ochraceopetaliformis* DSW-2, isolated from the sea water from Dongshan Island in Fujian Province of China, yielded an undescribed cyclic tripeptide, sclerotiotide M (**210**) (Figure 17), in addition to **197** and **201** (Figure 17). An extensive analysis of 1D and 2D NMR spectra and HRMS revealed the presence of Ala and Val, both of which are *N*-methylated, in addition to Orn. The interpretation of COSY and HMBC correlations allowed us to establish the sequence of amino acids as *cyclo*-(*N*-Me-Ala-*N*-Me-Val-Orn). The relative configuration of the stereocenters in **210** were established as 2S*,6S*,12S* by the calculated ^13^C NMR chemical shifts coupled with the DP4+ statistical method. Moreover, the optical rotation of **210** showed the same sign as those of the previously reported **196**, **197**, **199**–**201**, suggesting that they share the same absolute configuration. Thus, the structure of **210** was identified as (2′*E*)-*cyclo*-[(*N-*Me-L-Ala)-(*N-*Me-L-Val)-(*N*_α_-4′,4′-dimethoxy-2′-butenoyl-L-Orn)] [78].

#### 3.2.3. Cyclic Tetrapeptides

**Figure 18:**
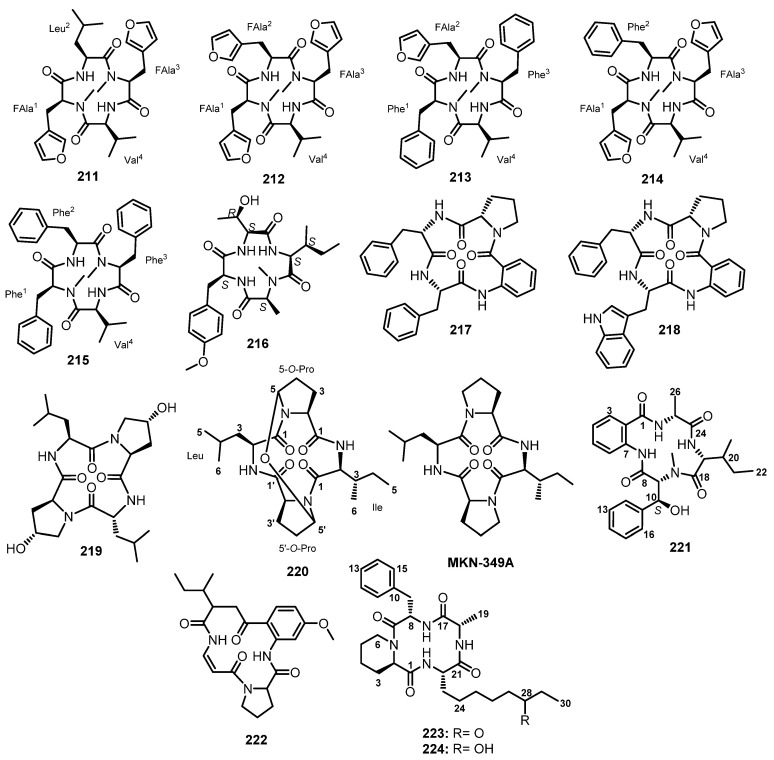
Structures of **211**–**224**.

Endolides A (**211**) and B (**212**) (Figure 18), two *N*-methylated peptides containing a rare amino acid, 3-(3-furyl)-Ala (33FAla), were isolated from the culture extract of the fungus *Stachylidium* sp., obtained from a marine sponge, *Callyspongia* sp. sf. *C. flammea,* which was collected in Bare Island, New South Wales, Australia. Extensive analysis of COSY and HMBC correlations allowed us to determine the amino acid subunits, as well as their sequence in **211** as *cyclo*-[*N*-Me-33FAla-Leu-*N*-Me-33FAla-Val], and in **212** as *cyclo*-[*N*-Me-33FAla-*N*-Me-33FAla-*N*-Me-33FAla-Val]. The configurations of the amino acid residues in **211** and **212** were established as L by advanced Marfey’s method, except for 33FAla since it underwent a degradation during a process of acidic hydrolysis. However, the authors were able to obtain a suitable crystal of **211** for X-ray crystallographic analysis showing that all four α protons have the same relative configuration. Thus, a combination of the results of Marfey’s and X-ray analyses led to the conclusion that the absolute configuration of the two *N*-Me-33FAla residues in **211** also has to be L. However, the authors assumed that the configuration of *N*-Me-33FAla in **212** was also L on the basis of biogenetic considerations [103].

In order to investigate the metabolic origin of the 33FAla moiety in the *Stachylidium* sp. peptides by using isotope-labeled precursors, the major metabolite **211** was targeted. Because the peptides are not produced in liquid media, solid biomalt sea-salt-containing agar media (BMS) were used for the investigation. Various ^13^C-labeled biosynthetic precursors such as L-[1-^13^C]-Phe, fully labeled [U-^13^C] glycerol, D-[1-^13^C] glucose, [1-^13^C] sodium acetate, and L-[Me-^13^C] Met were added to the solid BMS medium culture of the marine-derived fungus *Stachylidium* sp. 293 K04, which was isolated from the sponge *Callyspongia* sp. cf. *C. flammea*, collected from the coral reef in Bare Island, New South Wales, Australia. The results revealed that the exogenous Phe was not incorporated in the *N*-Me-33FAla residue. On the other hand, the biosynthesis of the *N*-Me-33FAla moiety involves both phosphoenolpyruvate and erythrose-4-phosphate as precursor molecules and the shikimate pathway is the biosynthetic route for the 33FAla moiety in **211**, with Met as a provider of the *N*-Me group. Moreover, during the course of this study, two structurally unprecedented 33FAla-containing cyclic *N*-methylated peptide analogues, endolides C (**213**) and E (**214**) (Figure 18), and the previously described hirsutide (**215**) (Figure 18) were also isolated. The HMBC correlations established the structure of **213** as *cyclo*-(*N*-Me-Phe-Val-Phe-33FAla), and of **214** as *cyclo*-(*N*-Me-33FAla-Val-*N*-Me-33FAla-Phe). The absolute configurations of Val and Phe were established as L by the advanced Marfey’s method while the L-configuration of 33FAla was based on biogenetic considerations as well as from the results obtained from the X-ray analysis of **211** [104].

The EtOAc extract of a culure of *A. violaceofuscus*, isolated from the marine sponge *Reniochalina* sp., which was collected from the Xisha Islands in the South China Sea, yielded an unreported cyclic tetrapeptide, violaceotide A (**216**) (Figure 18). The amino acid sequence of **216** was determined as *cyclo*-(Thr-*O*-Me-Tyr-*N*-Me-Ala-Ile) by detailed HMBC, NOESY, and mass fragmentation analyses using a quadrupole-time-of-flight tandem mass spectrometer (Q-TOF-MS/MS). Marfey’s method was used to established the L-configuration of all amino acids in **216** [99].

The EtOAc culture extract of a marine-derived fungus, *N. glabra* KUFA 0702, isolated from a marine sponge *Mycale* sp., collected at a depth of 15–20 m from the coral reef at Samaesarn Island in the Gulf of Thailand, furnished two undescribed cyclic tetrapeptides, sartoryglabramides A (**217**) and B (**218**) (Figure 18). Extensive analysis of COSY and HMBC correlations were used to identify their amino acid residues and their sequence of **217** as *cyclo*-(Abz-Phe-Phe-Pro), and of **218** as *cyclo*-(Abz-Trp-Phe-Pro). The absolute configurations of the amino acids in both compounds were determined by X-ray analysis and confirmed by chiral HPLC analyses of their acidic hydrolysate, thus establishing complete structures of **217** and **218** as *cyclo*-(Abz-L-Phe-L-Phe-L-Pro) and *cyclo*-(Abz-L-Trp-L-Phe-L-Pro), respectively [70].

Li et al. reported a new cyclic tetrapeptide, *cyclo*-(L-Leu-*trans*-4-OH-L-Pro-D-Leu-*trans*-4-OH-L-Pro) (**219**) (Figure 18), from the co-culture broth extract of *Phomopsis* sp. K38 and *Altenaria* sp. E55, isolated from mangrove which was collected from Leizhou Peninsula, Guangdong Province, China. The amino acid residues and their sequence were determined by 1D and 2D NMR spectral analysis while Marfey’s method was used to determine the absolute configuration of the amino acids [105].

Mycelial and broth extracts of a mangrove endophytic fungus, *Penicilluim* sp. GD6, isolated from the stem bark of a mangrove tree, *Bruguiera gymnorrhiza*, which was collected in Zhanjiang, China, yielded an undescribed cyclic tetrapeptide 5,5ʹ-epoxy-MKN-349A (**220**) (Figure 18). The amino acid residues, i.e., two Pro, one Leu, and one Ile, were identified by extensive analysis of 1D and 2D NMR spectra. The amino acid sequence of **220** was achieved by HMBC correlations. The structure of **220** was found to resemble that of MKN-349A, a cyclic tetrapeptide previously reported from a marine-derived bacterium, *Nocardiopsis* sp. [106]. Due to a negligible amount of **220**, the absolute configuration of its amino acid residues could not be determined by Marfey’s method. However, the authors proposed the L-configuration for all the amino acid residues on the basis of biogenic considerations [107].

The EtOAc extract of the mycelium of *A. terreus* SCSGAF0162, which was isolated from the tissue of a gorgonian, *Echinogorgia aurantiaca*, collected from Sanya, Hainan Province, China, furnished asperterrestide A (**221**) (Figure 18). Detailed analysis of 1D and 2D spectral data and the loss of fragments from the ESIMS of **221** led to the identification of Abz and other amino acid units, including 3-OH-*N*-Me-Phe, Ile, and Ala. The amino acid sequence of **221** was deduced as *cyclo*-(3-OH-*N*-Me-Phe-Abz-Ala-Ile) from NOESY correlations between α-protons and the protons of the NH groups of neighboring residues, as well as from HMBC correlations. The absolute configurations of Ala and Ile residues were determined as D by Marfey’s method, while the absolute configuration at C-10 was unambiguously assigned as *S* by a modified Mosher’s method in the NMR tube. Consequently, the structure of **221** was elucidated as *cyclo*-(Abz-D-Ala-D-Ile-3(*S*)-OH-*N*-Me-Phe) [108].

The EtOAc extract of a culture broth of *A. flavipes*, obtained from the gut of a marine isopod, *Ligia oceanica*, which was collected in Zhoushan, Zhejiang province of China, yielded a cyclic tetrapeptide **222** (Figure 18) whose structure consists of Pro, Ile, and two unusual amino acids, 5-methoxyanthranillic acid (5-OMe-Abz), and aminoacrylic acid. The identification of the amino acid residues was based on the interpretation of 1D and 2D NMR spectr, a while the amino acid sequence, *cyclo*-(5-OMe-Abz-Pro-Ile-aminoacrylic acid), was deduced from HMBC correlations and the major ESI-MS^n^ fragment ion peaks. However, the configurations of Pro and Ile were not determined [109].

In a search for new metabolites that inhibit cancer cell proliferation from cultured marine fungi, Gu et al. reported the isolation of two undescribed cyclic tetrapeptides microsporins A (**223**) and B (**224**) (Figure 18), 12-membered ring cyclic peptides containing all α amino acids, from the culture extracts of the *Microsporum* cf. *gypseum* strain CNL-629, obtained from a marine bryozoan *Bugula* sp., which was collected in the U.S. Virgin Islands. Then, 1D and 2D NMR spectral analysis, including 1D-TOCSY and a combination of 1D-TOCSY with HMBC, revealed the presence of one Ala, one Phe, and one pipercolic acid (Pip) residues, and an unusual amino acid, 2-amino-8-oxo-decanoic acid (Aoda) in **223**. The sequence of the four amino acids was established as *cyclo*-(Pip-Phe-Ala-Aoda) by HMBC correlations from the carbonyl carbon of each amino acid with the corresponding NH proton of the adjacent amino acid. By the same way, one Ala, one Phe, one Pip residues, and 2-amino-8-hydroxydecanoic acid, a hydroxyl derivative of Aoda were identified in **224**. The configuration of Ala, Phe, and Pip were established as D-Pip, L-Phe, and D-Ala for **224**. The *S* configuration of the stereogenic carbon of Aoda (C-22) in **223** was established based on NOE correlations between the α-H of Ala and the α-H of Aoda with Ala NH. However, the absolute configuration at the C-28 (the hydroxy-bearing carbon of the 2-amino-8-hydroxydecanoic acid residue) of **224** was not determined due to an insufficient amount of the compound to perform Mosher’s analysis [110].

#### 3.2.4. Cyclic Pentapeptides

**Figure 19:**
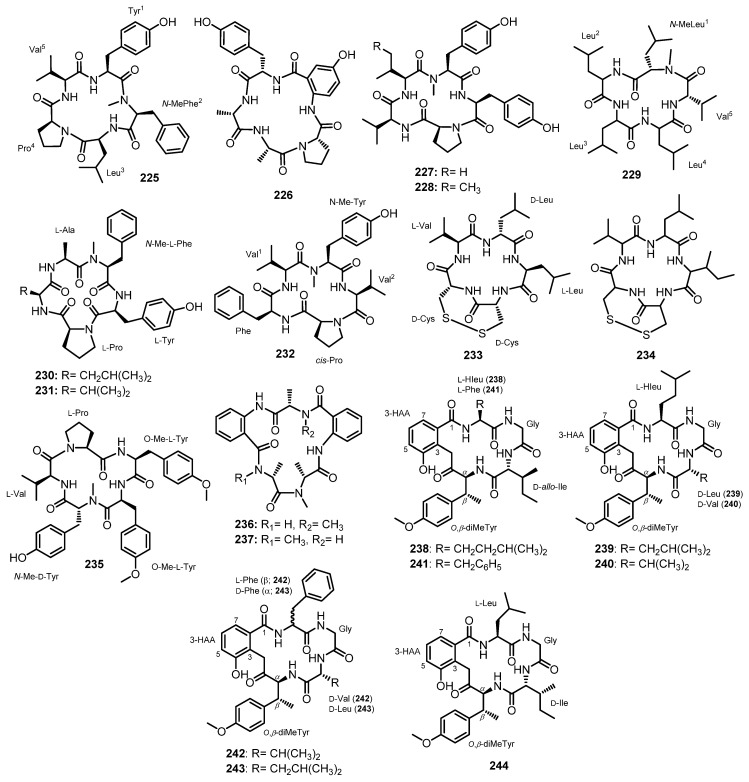
Structures of **225**–**244**.

The solid-phase culture extract of *Aspergillus* sp. MEXU27854, isolated from a sample of sand collected from the intertidal zone of Caleta Bay in Acapulco, Guerreo, Mexico, furnished a new *N*-methyl cyclic pentapeptide, caletasin (**225**) (Figure 19). A combination of 1D and 2D NMR spectra, especially the TOCSY spectrum and MS/MS analysis, established the preliminary structure of **225** as *cyclo*-(Tyr-*N*-Me-Phe-Leu-Pro-Val). Marfey’s method was used to determine the absolute configuration of the amino acid residues; however, it was not possible to achieve a full separation of all the amino acids present in the mixture of the derivatized standards under any of the experimental conditions, especially between L-Leu and D-Val. A comparison of the mass profiles and mass values from HRESI-MS data for each amino acid confirmed the presence of L-Leu. Thus, the complete structure of **225** was established as *cyclo*-(L-Tyr-*N*-Me-L-Phe-L-Leu-L-Pro-L-Val) [111].

Asperpeptide A (**226**) (Figure 19), was isolated from the mycelial and broth extracts of *Aspergillus* sp. SX-20090B15, which was obtained from the inner tissue of a fresh gorgonian *Muricella abnormaliz*. Extensive analysis of HMBC and COSY correlations revealed the presence of one Tyr, two Ala, one Pro, and one 5-OH-Abz. The amino acid sequence was established as *cyclo*-(Pro-Ala-Ala-Tyr-5-OH-Abz) by HMBC correlations and the interpretation of fragment ions in the ESIMS/MS spectrum. The configurations of all the amino acid residues were determined as L by Marfey’s method. Thus, the complete structure of **226** was established as *cyclo*-(L-Pro-L-Ala-L-Ala-L-Tyr-5-OH-Abz) [46].

Fremlin et al. described the isolation of cotteslosins A (**227**) and B (**228**) (Figure 19) from the fermentation extract of *A. versicolor* (MST-MF495), which was obtained from a beach sand sample collected at a low tide from Cottesloe, Western Australia. The (+)-HRESI-MS and NMR spectral analysis of **227** revealed the presence of five amino acid residues. By using C_3_ Marfey’s analysis, the amino acids were identified as L-Tyr, *N*-Me-L-Tyr, L-Pro, L-Val, and L-Val. HMBC correlations were used to establish the sequence L-Tyr-*N*-Me-L-Tyr-L-Val-L-Val-L-Pro, and required cyclization from the L-Pro nitrogen to the L-Tyr carboxyl. By a comparison of the NMR spectra and (+)-HRESI-MS of **228** with those of **227**, and with the support of C_3_ Marfey’s analysis, the sequence of **228** was established as L-Tyr-*N*-Me-L-Tyr-L-*allo*-Ile-L-Val-L-Pro. Thus, **228** is the L-Val^1^ to L-*allo*-Ile variant of **227** [112].

Lajollamide A (**229**) (Figure 19) was isolated from the culture broth extract of a marine-derived fungus, *Asteromyces cruciatus* 763, which was obtained from an unidentified decaying green alga, collected at La Jolla Shores, San Diego, USA. Then, a 1D and 2D NMR interpretation of **229** identified the individual amino acids as one Val, three Leu, and one *N*-Me-Leu residues. HMBC correlations from NH and NCH_3_ signals to the carbonyl groups of the adjacent amino acids were used to determine the amino acid sequence as *N*-Me-Leu-Leu-Leu-Leu-Val. The cyclic nature of **229** was confirmed by the degrees of unsaturation and HMBC correlations from the NCH_3_ signal of the *N*-Me-Leu residue to the carbonyl of Val. Chiral HPLC analysis of the acid hydrolysate of **229** resulted in the identification of the configurations of the amino acid residues as *N*-Me-L-Leu, two L-Leu, one D-Leu, and L-Val. However, the position of D-Leu could not be identified by the NMR data. A comparison of the NMR data of the three synthetic diastereomers of **229** with those of naturally occurring **229** to localize the position of D-Leu led to the conclusion that the D-Leu residue present in the hydrolysate was a result of the epimerization of the original L-Leu during the process of hydrolysis. Thus, the structure of **229** was established as *cyclo*-(*N*-Me-L-Leu-L-Leu-L-Leu-L-Leu-L-Val) [83].

JG002CPA (**230**) and JG002CPB (**231**) (Figure 19) were also isolated from the solid rice culture extract of a marine-derived fungus, *A. allahabadii* (strain number JG002), which was obtained from the underwater sediment collected off the coast of Jaeju-do Island, Korea. The 1D and 2D NMR spectral analysis, especially COSY and HMBC correlations, led to the identification of the amino acid residues and their sequences which were confirmed by high-resolution LC-MS/MS analysis, while the L-configuration of the amino acids was determined by the advanced Marfey’s method. Consequently, the complete structures of **230** and **231** were established as *cyclo*-(*N*-Me-L-Phe-L-Tyr-L-Pro-L-Leu-L-Ala) and *cyclo*-(*N*-Me-L-Phe-L-Tyr-L-Pro-L-Val-L-Ala), respectively [51].

The EtOAc extract of a solid rice culture of a marine-derived fungus, *A. flocculosus* 16D-1, isolated from the inner tissue of a marine sponge *Phakellia fusca*, which was collected from Yongxing Island, China, furnished an undescribed *N*-methylated pentapeptide, asperflomide (**232**) (Figure 19). An analysis of the 1D and 2D NMR spectra revealed the pentapeptide nature of **232**. An analysis of the TOCSY spectrum revealed the presence of two Val (Val^1^ and Val^2^), one *N*-Me-Tyr, one Pro, and one Phe residues. An analysis of the COSY, HMBC, and TOCSY correlations established the amino acid sequence as Val^1^-*N*-Me-Tyr-Val^2^-Pro-Phe. The ROESY correlations were used to connect the final sequence as *cyclo*-(Val^1^-*N*-Me-Tyr-Val^2^-Pro-Phe), which was confirmed by MS/MS fragmentation. The absolute configuration of the amino acids in **232** was established by Marfey’s method. Thus, the complete structure of **232** was elucidated as *cyclo*-(L-Val^1^-*N*-Me-L-Tyr-L-Val^2^-L-Pro-L-Phe) [113].

The CH_2_Cl_2_-soluble fraction of a salt water culture of *A. niger*, isolated from a Caribbean sponge *Hyrtiosproteus* sp., furnished the previously reported cyclic pentapeptide, malformin C (**233**) (Figure 19) [97]. Later on, Uchoa et al. isolated the previously described malformin A1 (**234**) (Figure 19), together with malformin C (**233**) from a liquid culture extract of a marine sediment-derived fungus, *A. niger* BRF-074, collected from the Northeast Brazilian coast [65]. Compound **234** was also reported from the culture extract of a marine sediment-derived fungus *Aspergillus* sp. SCSIOW2 [114].

A chemical investigation of the culture broth extract of a marine-derived fungus, *Aspergillus* sp. SCSIO 41501, which was isolated from the gorgonian *Melitodes squamata,* collected from the South China Sea, furnished an undescribed aspergillipeptide D (**235**) (Figure 19). A detailed 1D and 2D NMR analysis, followed by Marfey’s analysis of the hydrolysate of **235**, established the sequence of the amino acid residues and their absolute configurations as *cyclo*-(L-Val-*N*-Me-D-Tyr-*O-*Me-L-Tyr-*O*-Me-L-Tyr-L-Pro) [41].

The EtOAc extract of a culture of *A. versicolor* strain ZLN-60, which was isolated from the mud, at a 20 m depth, of the Yellow Sea, China, afforded two undescribed isomeric cyclic pentapeptides, versicotides A (**236**) and B (**237**) (Figure 19). An analysis of COSY and HMBC correlations of **236** and **237** revealed the presence of one Ala, two Abz, and two *N*-Me-Ala residues. Marfey’s method was used to determine the absolute configurations of the amino acid residues while HMBC correlations were used to establish the amino acid sequence. Consequently, the structures of **236** and **237** were established as *cyclo-*(L-Ala-*N*-Me-L-Ala-Abz-*N*-Me-L-Ala-Abz) and *cyclo*-(*N*-Me-L-Ala-*N*-Me-L-Ala-Abz-L-Ala-Abz), respectively [115].

Ding et al. used the LC-MS/MS-based molecular networking approach to assist the isolation of seven undescribed cyclopentapeptides, pseudoviridinutans A-G (**238**–**244**) (Figure 19), which feature a rare amino acid residue, *O*,*β*-dimethyltyrosine (*O*,*β*-diMeTyr), from the solid rice culture extract of *A. pseudoviridinutans* TW58-5, isolated from a hydrothermal vent sediment collected in Kueishantao, Taiwan. The planar structures of **238**–**244** were elucidated by a detailed analysis of HR ESI-QTOF MS, and 1D and 2D NMR spectroscopic data. The 1D and 2D NMR analysis of **238** revealed the presence of three common amino acids, *viz.* Ile, Gly, and homoleucine (Hleu), as well as two rare moieties, i.e., *O*,*β*-diMeTyr and (3-OH-Abz). HMBC correlations of α-H of one amino acid to the carbonyl of a neighboring amino acid established the amino acid sequence of **238** as *cyclo-*[(*O,β*-diMeTyr)-Ile-Gly-Hleu-3-OH-Abz]. The absolute configurations of Hleu and Ile were established as L-Hleu and D-*allo-*Ile, respectively, by Marfey’s analysis. Due to the unavailability of standard *O*,*β*-diMeTyr, the absolute configurations of its stereogenic carbons (C_α_ and C_β_) could not be determined by Marfey’s method. Since **238** was obtained in a suitable crystal for X-ray analysis, the absolute configurations of C_α_ and C_β_ were determined as *S* and *R*, respectively. Thus, the complete structure of **238** is *cyclo-*[(α*S*, β*R*)(*O*,*β*-diMeTyr)-D-*allo*-Ile-Gly-L-Hleu-3-OH-Abz-] [116].

Compound **239** is similar to **238** except for the presence of Leu in **239** instead of Ile in **238**. The amino acid sequence in **239** was established by the interpretation of HMBC correlations as *cyclo* [(*O*,*β*-diMeTyr)-Leu-Gly-Hleu-3-OH-Abz], and this sequence was supported by its fragmentation pattern in MS/MS. The absolute configurations of the Hleu and Leu residues in **239** were determined as L-Hleu and D-Leu, respectively, by Marfey’s method. Since **239** displayed the same coupling constant of α-H with β-H (*J*_α,β_ = 4.1 Hz) for the *O*,*β-*diMeTyr residue, as observed in **238,** it, thus, supports the same relative configurations of C_α_ and C_β_. The absolute configurations of C_α_ and C_β_ in **239** were suggested to be the same as those of C_α_ and C_β_ in **238** based on the NMR data and biosynthetic origin [116].

By using the same methods, the amino acid residues, their stereochemistry, and sequence in **240** were established as *cyclo*-[-(α*S*, β*R*)(*O*,*β*-diMeTyr)-D-Val-Gly-L-Hleu-3-OH-Abz], while the structures of **241** was established as *cyclo-*[-(α*S*, β*R*)(*O*,*β*-diMeTyr)-D-*allo*-Ile-Gly-L-Phe-3-OH-Abz], of **242** as *cyclo-*[-(α*S*, β*R*)(*O,β*-diMeTyr)-D-Val-Gly-L-Phe-3-OH-Abz], of **243** as *cyclo-*[-(α*S*, β*R*)(*O,β*-diMeTyr)-D-Leu-Gly-D-Phe-3-OH-Abz], and of **244** as *cyclo-*[-(α*S*, β*R*)(*O*,*β*-diMeTyr)-L-Leu-Gly-D-Ile-3-OH-Abz] [116].

#### 3.2.5. Cyclic Hexapeptides

**Figure 20:**
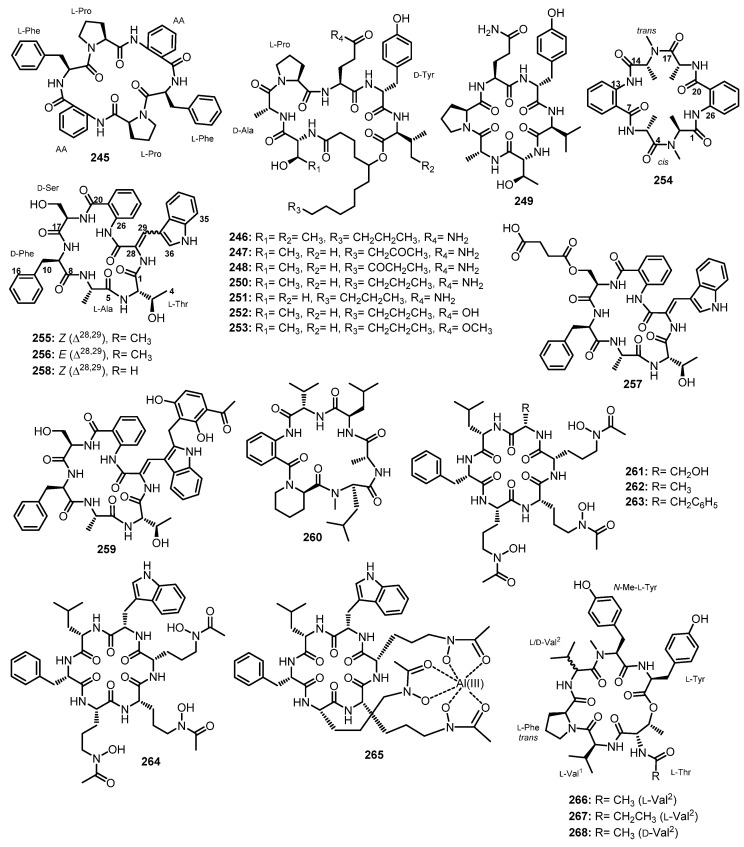
Structures of **245**–**268**.

An undescribed centrally symmetrical cyclohexapeptide, aspersymmetide A (**245**) (Figure 20), was isolated from the solid rice culture extract of a marine-derived fungus, *A. versicolor* (TA01-14), obtained from a gorgonian coral *Carijoa* sp. (GX-WZ-2010001), which was collected from the Weizhou coral reefs in the South China Sea. Detailed analysis of 1D and 2D NMR spectral data allowed us to identify the presence of one Pro, one Phe, and one Abz residues, which corresponded to half of the proposed molecular formula obtained from (+)-HRESI-MS, suggesting **245** as a symmetrical dimer. An analysis of HMBC correlations from the signals of NH proton of one amino acid to the carbonyl of the adjacent amino acid led to the deduction of their sequence as Phe-Abz-Pro. The HMBC correlation from α-H of Pro to the carbonyl of Phe connected the two half sequences to establish the whole cyclic structure of **245** as *cyclo*-(CO-Phe–Abz–Pro–Phe–Abz–Pro-N). The L-configuration of Pro and Phe was established by Marfey’s analysis. Thus, the complete structure of **245** was elucidated as *cyclo*-(L-Phe–Abz–L-Pro–L-Phe–Abz–L-Pro) [117].

Liang et al. described the isolation of four unreported cyclohexapeptides, simplicilliumtides J-M (**246**–**249**), together with the previously reported verlamelins A (**250**) and B (**251**) (Figure 20), from a fermentation broth extract of a deep-sea-derived fungus, *S. obclavatum* EIODSF 020, which was isolated from a marine sediment sample collected in the East Indian Ocean. Detailed 1D and 2D NMR analysis, especially COSY and HMBC correlations, suggested the presence of a long chain fatty acid, 5-hydroxytetradecanoic acid (5HTA), in **246**, **250**, and **251**, while **247** and **248** contained 5-hydroxy-13-ketotetradecanoic acid (5H13KTA) and 5-hydroxy-12-ketotetradecanoic acid (5H12KTA), respectively. Marfey’s and Mosher’s methods were used to establish the absolute configurations of amino acids and the hydroxy-bearing carbons on long chain fatty acids, respectively. The sequences of amino acids were achieved by an analysis of the HMBC correlations of the α-proton of one amino acid to the carbonyl carbon of the next one. Thus, the complete structures of **246**–**249** were established as *cyclo*-(L-*allo*-Ile-D-Tyr-L-Gln-L-Pro-D-Ala-D-*allo*-Thr-5(*S*)HTA), *cyclo*-(L-Val-D-Tyr-L-Gln-L-Pro-D-Ala-D-*allo*-Thr-5(*S*)H13KTA), *cyclo*-(L-Val-D-Tyr-L-Gln-L-Pro-D-Ala-D-*allo*-Thr-5(*S*)H12KTA), and *cyclo*-(L-Val-D-Tyr-L-Gln-L-Pro-D-Ala-D-*allo*-Thr), respectively [49].

Later on, the same group has described the isolation of two undescribed cyclohexapeptides, simplicilliumtides N (**252**) and O (**253**) (Figure 20), together with **246**, **250**, and **251**, from the combined broth and mycelia extracts of the same fungus. The identity and the sequence of the amino acids were established by a detailed analysis of the 1D and 2D NMR spectra, especially HMBC correlations, while the absolute configurations of the amino acid residues were determined by Marfey’s analysis. Thus, the structure of **252** was elucidated as *cyclo*-(L-Val-D-Tyr-L-Glu-L-Pro-D-Ala-D-*allo*-Thr-5HTA). The structure of **253** is similar to that of **252** except for the presence of a methyl ester of L-Glu in **253** instead of L-Glu in **252**. Thus, **253** is a methyl ester of **252**. The configuration of the 5HTA moiety in both **252** and **253** was inferred to be the same as that of 5HTA in **246**, **250**, and **251** [118].

Versicotide C (**254**) (Figure 20) was also isolated from the culture extract of a marine mud-derived fungus, *A. versicolor* ZLN-60. Detailed analysis of 1D and 2D NMR spectra, together with the molecular formula obtained from HRESI-MS, allowed us to identify six amino acid residues, i.e., two Ala, two *N*-Me-Ala, and two Abz residues. The sequence of the six amino acids was established, by observing HMBC correlations from α-H and *N*-Me protons, as well as amide NH signals to the carbonyl carbons of the connecting amino acid, as *cyclo*-(Me-Ala-Ala-Abz-Me-Ala-Ala-Abz), while Marfey’s analysis was used to identify the configuration of L-Ala and *N*-Me-L-Ala. Thus, the complete structure of **254** was elucidated as *cyclo*-(*N*-Me-L-Ala-L-Ala-Abz-*N*-Me-L-Ala-L-Ala-Abz). Interestingly, **254** displayed two sets of NMR signals of the two *N*-Me, indicating different configurations of the two corresponding amide bonds that are formed from *N*-Me-Ala. Thus, the *cis/trans* conformation was proposed for **254**, and this was confirmed by variable-temperature ^1^H NMR experiments, which showed a significant decrease in the number of ^1^H NMR resonances measured at 80 °C compared to that measured at 25 °C [101].

The fermentation extract of *A. sclerotiorum* PT06-1, isolated from the Putian Sea Salt Field, Fujian, China, and cultured in a nutrient-limited hypersaline medium containing 10% NaCl, yielded two undescribed cyclic hexapeptides, sclerotides A (**255**) and B (**256**) (Figure 20). Detailed analysis of 1D and 2D NMR spectra, in combination with the molecular formula of **255** obtained from (+)-HRESI-MS, led to the identification of six amino acid residues viz. Thr, Ala, Phe, Ser, Abz, and dehydrotryptophan (∆-Trp). The amino acid sequence of **255** was established as *cyclo*-(Thr-Ala-Phe-Ser-Abz-∆-Trp) by HMBC correlations from the carbonyl carbon of one amino acid residue to the amide protons of the neighboring residue [119].

Compound **256**, an isomer of **255**, was found to have the same amino acid residues and sequence as those in **255**. By the observation of the chemical shift values of H-29, H-38 and the amide proton of ∆-Trp, it was found that **256** is a geometric isomer of **255**, differing only in the geometry of the double bond between C-28 and C-29 of ∆-Trp, being *Z* in **255** and *E* in **256**. The absolute configurations of the amino acid residues in **255** and **256** were determined by chiral HPLC using a Crownpak CR(+) column. Thus, the structures of **255** and **256** were identified as *cyclo*-(L-Thr-L-Ala-D-Phe-D-Ser-Abz-*Z*-Δ-Trp) and *cyclo*-(L-Thr-L-Ala-D-Phe-D-Ser-Abz-*E*-Δ-Trp), respectively [119].

Compounds **255** and **256** were stable in the dark, but changed into each other in light, indicating that **255** and **256** are photointerconvertible. HPLC analysis showed that the ratio of **255** and **256** was 87:13 in the equilibrium, and both temperature and solvent had little influence on the photoequilibrium. All the evidences suggested that the photointerconversion proceeded through a radical mechanism and the *Z*-isomer of **255** is more stable. Therefore, it is most probable that **255** was really produced by *A. sclerotiorum*, while **256** was formed from the photoreaction of **255** during the fermentation or subsequent isolation steps [119].

Further three undescribed cyclic hexapeptides of the sclerotide family, sclerotides C-E (**257**–**259**) (Figure 20), were isolated together with **255** (Figure 20), from the extract of a solid rice culture of *A. sclerotiorum* SCSIO 41031, obtained from a soft coral which was collected in Beihai, Guangxi Province, China. Detailed analysis of 1D and 2D NMR spectra and HRESI-MS spectrum were used to identify the amino acid residues in these compounds, while their amino acid sequences were established by HMBC correlation of the α-H and NH signals of one amino acid to the carbonyl carbon of the connecting amino acid. Thus, the amino acid sequence of **257** was established as *cyclo*-(Thr-Ala-Phe-butanoic acid serine ester (BASE)-Abz-Δ-Trp). The geometry of the double bond between C-28 and C-29 in the Δ-Trp residue was the same as that in **255**, i.e., *Z*-geometry. X-ray analysis not only confirmed the structure of **257** but also determined the configuration of its amino acid residues. Thus, the complete structure of **257** was elucidated as *cyclo*-(L-Thr-L-Ala-D-Phe-D-BASE-Abz-*Z*-Δ-Trp) [120].

An analysis of the 1D and 2D NMR and HRESI-MS spectra of **258** revealed that it had the same carbon backbone as that of **255**, and absolute configurations of the amino acid residues were identified as L-Ser, L-Ala, D-Phe, and D-Ser by Marfey’s method. Thus, the structure of **258** was established as *cyclo*-(L-Ser-L-Ala-D-Phe-D-Ser-Abz-Z-∆-Trp). For **259**, the 1D and 2D NMR and HRESI-MS spectral analysis revealed the presence of 2-(3-acetyl-2,6-dihydroxyphenyl)-*N*-∆-acetyltryptamine (ADPAT), in addition to Thr-Ala-Phe-Ser-Abz. Key HMBC correlations and the ESIMS/MS spectrum allowed us to establish the connectivity of the six amino acid residues, while Marfey’s analysis was used to determine the absolute configuration of the amino acids. Thus, the complete structure of **259** was established as *cyclo*-(L-Thr-L-Ala-D-Phe-D-Ser-Abz-Z-ADPAT) [120].

Similanamide (**260**) (Figure 20) is a cyclohexapeptide isolated from the EtOAc extract of a solid rice culture of *A. similanensis* KUFA0013, which was obtained from the marine sponge *Rhabdermia* sp., collected from the coral reef of the Similan Islands, Phang Nga Province, Thailand. Detailed analysis of the 1D and 2D NMR and HRESI-MS spectra was used to determine the six amino acid residues and their sequence as *cyclo-*(Abz-Val-Leu-Ala-*N*-Me-Leu-Pip). Chiral HPLC analysis of the acid hydrolysate of **260** led to the identification of the absolute configurations of the amino acids. Thus, the complete structure of **260** was elucidated as *cyclo*-(Abz-L-Val-D-Leu-L-Ala-*N*-Mel-L-Leu-D-Pip) [121].

The culture extract of *Acremonium persicinum* SCSIO115, which was obtained from a marine sediment collected from the South China Sea, resulted in the isolation of four undescribed cyclic hexapeptide siderophores, acremonpeptides A–D (**261**–**264**) (Figure 20), together with the aluminium complex of **264**, Al(III)-acremonpeptide D (**265**) (Figure 18). An analysis of the 1D and 2D NMR and HRESI-MS spectra revealed that **261**–**265** all contain three units of 2-amino-5-(*N*-hydroxyacetamido)pentanoic acid (*N*^5^-OH-*N*^5^-Ac-L-Orn), which is able to chelate metal ions, besides other common amino acids. Marfey’s method was used to determine the absolute configurations of the amino acids in **261**–**264,** and their sequences were established by an analysis of HMBC correlations from the α-H and NH of one amino acid to the carbonyl carbon of the connecting amino acid. Thus, the structures of **261**–**264** were elucidated as *cyclo*-(L-Phe-L-Leu-L-Ser-L-AcN(OH)-Orn^1^-L-AcN(OH)-Orn^2^-L-AcN(OH)-Orn^3^), *cyclo*-(L-Phe-L-Leu-L-Ala-L-AcN(OH)-Orn^1^-L-AcN(OH)-Orn^2^-L-AcN(OH)-Orn^3^), *cyclo*-(L-Phe-L-Leu-L-Phe-L-AcN(OH)-Orn^1^-L-AcN(OH)-Orn^2^-L-AcN(OH)-Orn^3^), and *cyclo*-(L-Phe-L-Leu-L-Trp-L-AcN(OH)-Orn^1^-L-AcN(OH)-Orn^2^-L-AcN(OH)-Orn^3^), respectively. The amino acid sequence of **265** was found to be the same as that of **264**. However, the ^13^C NMR chemical shifts of the methyl carbons of the three acetyl groups were found to appear at lower frequencies, at *δ*_C_ 15.8, 16.3, and 16.7, when compared to those of the methyl carbons of the three acetyl groups in **264** (*δ*_C_ 20.5), which is consistent with the aluminium ion chelation by **264** to form **265** [122].

Tang et al. described the isolation of three unreported petrosamides A-C (**266**–**268**) (Figure 20) from the culture extract of *Aspergillus* sp. 151304, obtained from the inner tissue of a marine sponge *Petrosia* sp., which was collected from Yongxing Island, China. The six amino acid residues (one Pro, one Tyr, one Ac-Thr, one *N*-Me- Tyr, and two Val residues in **266**; one Tyr, one *N*-Me-Tyr, one Pro, two Val, and one propylated Thr for **267**; and two Tyr, two Val, one Pro and one Ac-Thr in **268**) were identified by a detailed analysis of the 1D and 2D NMR spectra and HRESI-MS data, while the sequences of the amino acids were established using HMBC and ROESY correlations from NH or α-H of one amino acid to the carbonyl carbon of the connecting amino acid. The absolute configurations of the amino acid residues were determined by advanced Marfey’s analysis. Thus, the structures of **266**–**268** were elucidated as *cyclo*-(L-Tyr-*N*-Me-L-Tyr-L-Val^2^-L-Pro-L-Val^1^-*N*-Ac-Thr), *cyclo*-(L-Tyr-*N*-Me-L-Tyr-L-Val^2^-L-Pro-L-Val^1^-*N*-Pr-Thr), and *cyclo*-(L-Tyr^2^-L-Tyr^1^-D-Val^2^-L-Pro-L-Val^1^-*N*-Ac-Thr), respectively. The *trans* geometry for the proline amide bond in the three compounds was evident from the Δδ_Cβ-Cγ_ values, which is 5.0 ppm in **266**, 5.1 ppm in **267**, and 4.7 ppm in **268**. The amino acid sequences were further confirmed by the corresponding ESIMS/MS fragments [123].

#### 3.2.6. Cyclic Heptapeptides

**Figure 21:**
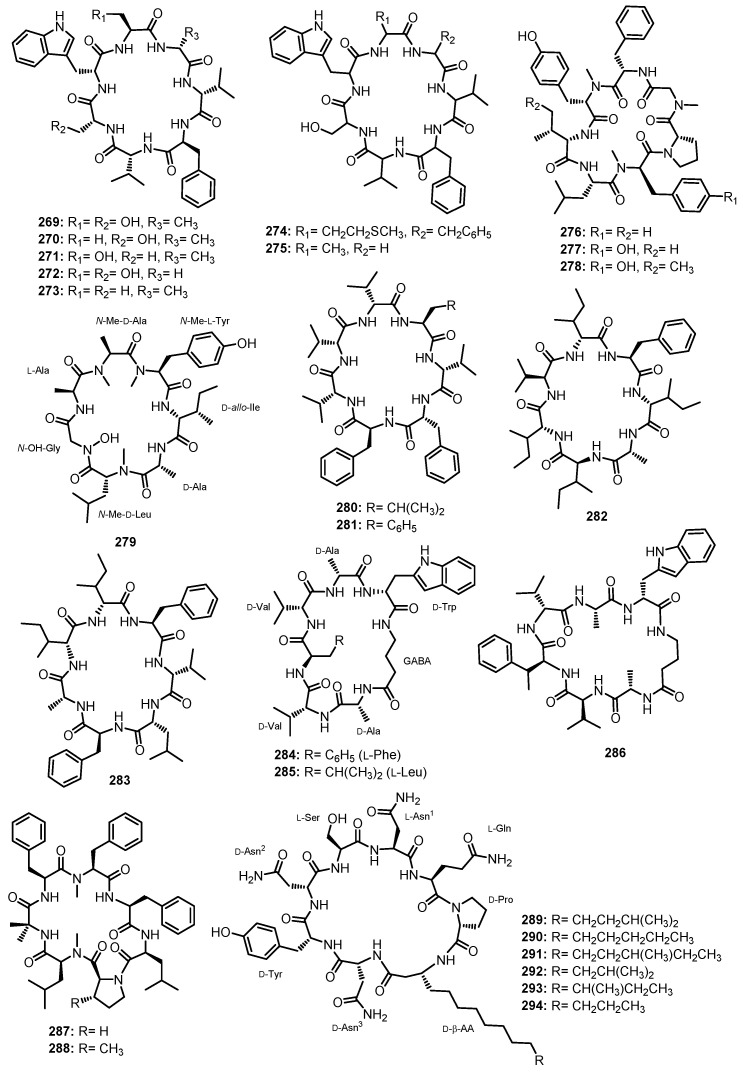
Structures of **269**–**294**.

By integrating molecular networking (untargeted HPLC-MS/MS) and ^1^H-NMR techniques, Shao’s group isolated three unreported cycloheptapeptides, asperversiamides A-C (**269**–**271**) (Figure 21), from the EtOAc extract of the coral-associated fungus *A. versicolor* (CHNSCLM-0063), collected from the South China Sea. Interpretation of 1D and 2D NMR spectra, together with the molecular formula obtained from HRESI-MS, led to the conclusion that **269** is a cyclic peptide containing one Phe, one Trp, one Ala, two Val, and two Ser residues. The sequence of the seven amino acids was deduced by HMBC correlations from the signals of NH to its respective adjacent carbonyl carbons, as *cyclo*-(Ser^2^-Trp-Ser^1^-Ala-Val^1^-Phe-Val^2^), which was confirmed by key ESI-MS/MS fragment ions. The same methods were applied to **270** and whose amino acid backbone was deduced as *cyclo*-(Ser-Trp-Ala^2^-Ala^1^-Val^1^-Phe-Val^2^) [124].

The configurations of amino acids were established by Marfey’s method which confirmed the presence of D-Ser, D-Trp, L-Ser, D-Ala, D-Val, and L-Phe in **269**, and D-Ser, D-Trp, L-Ala, D-Ala, D-Val, and L-Phe in **270**, respectively. However, the locations of D-Ser and L-Ser in **269**, and L-Ala and D-Ala in **270** could not be confirmed. The problem was solved by performing a total synthesis of **269** and **270** and their corresponding isomers. By a comparison of the ^1^H NMR data and the retention times of the synthetic and naturally occurring counterparts, the structures of **269** and **270** were confirmed as *cyclo-*(D-Ser^2^–D-Trp–L-Ser^1^–D-Ala^1^–D-Val^1^–L-Phe–D-Val^2^) and *cyclo*-(D-Ser^2^–D-Trp–L-Ala^2^–D-Ala^1^–D-Val^1^–L-Phe–D-Val^2^), respectively. The amino acid residues of **271** were the same as those of **270**, differing only in their connection sequence. HMBC correlations and Marfey’s method elucidated the structure of **271** as *cyclo-*(D-Ala^2^–D-Trp–L-Ser–D-Ala^1^–D-Val^1^–L-Phe–D-Val^2^), which was also supported by ESI-MS/MS key fragmentations [124].

In a further study, Shao’s group also used the ESI-MS/MS-based molecular networking strategy to isolate another four unreported cyclopeptides, asperheptatides–D (**272**–**275**) (Figure 21), in addition to **269**–**271**, from the solid rice culture extract of the same fungus, obtained from the marine gorgonian coral *Rumphella aggregate*, which was collected from Nansha Islands in the South China Sea. Based on ^1^H and ^13^C NMR spectral analysis, the molecular formula obtained from the HRESI-MS, HMBC correlations, and key ESI-MS/MS fragment ions, the planar structures of **272** and **273** were established as *cyclo-*(Ser^2^-Trp-Ser^1^-Gly-Val^1^-Phe-Val^2^) and *cyclo-*(Ala^2^-Trp-Ala^1^-Ala^3^-Val^1^-Phe-Val^2^), respectively. Marfey’s method confirmed the presence of L-Phe, D-Trp, D-Val, D-Ser, and L-Ser in **272**, and D-Ala, L-Ala, L-Phe, D-Trp, and D-Val in **273**. The locations of L-Ser and D-Ser in **272**, and L-Ala and D-Ala in **273** were determined based on their shared biogenesis with **269**–**271**. Thus, the complete structures of **272** and **273** were elucidated as *cyclo-*(D-Ser^2^-D-Trp-L-Ser^1^-Gly-D-Val^1^-L-Phe-D-Val^2^) and *cyclo-*(D-Ala^2^-D-Trp-L-Ala^1^-D-Ala^3^-D-Val^1^-L-Phe-D-Val^2^), respectively. Since **274** and **275** were isolated in trace amounts, their planar structures were proposed as *cyclo-*(Ser-Trp-Met-Phe-Val-Phe-Val) and *cyclo-*(Ser-Trp-Ala-Gly-Val-Phe-Val) by ESI-MS/MS fragmentation experiments [125].

The unreported cordyheptapeptides C–E (**276**–**278**) (Figure 21) were isolated from a liquid culture extract of *A. persicinum* SCSIO115, which was obtained from a marine sediment sample collected at a depth of 205 m in the South China Sea. Detailed analysis of 1D and 2D NMR spectra and HRESI-MS of **276** revealed the presence of *N*-Me-Tyr, Phe, *N*-Me-Gly, Pro, *N*-Me-Phe, Leu, and Val. The sequence of the amino acids was determined by HMBC correlations and confirmed by analyses of the ESIMS/MS data of the quasi-molecular ion. The cyclic nature of the peptides was deduced by the degrees of unsaturation. Thus, the planar structure of **276** was established as *cyclo*-(*N*-Me-Tyr-Phe-*N*-Me-Gly-Pro-*N*-Me-Phe-Leu-Val). The absolute configuration of the amino acid residues was determined by X-ray analysis using Cu Kα radiation. Thus, the complete structure of **276** was established as *cyclo*-(*N*-Me-L-Tyr-L-Phe-*N*-Me-Gly-L-Pro-*N*-Me-D-Phe-L-Leu-L-Val). This was in agreement with the results obtained from Marfey’s analysis. The planar structure of **277** was established as *cyclo*-(*N*-Me-Tyr-Phe-*N*-Me-Gly-Pro-*N*-Me-Tyr-Leu-Val). By using Marfey’s analysis to determine the absolute configuration of the amino acids, the complete structure of **277** was elucidated as *cyclo*-(*N*-Me-L-Tyr^1^-L-Phe-*N*-Me-Gly-L-Pro-*N*-Me-D-Tyr^2^-L-Leu-L-Val) [126].

Detailed analyses of the COSY, HMQC, and HMBC correlations of **278** revealed that its structure was similar to that of **277**. The ^1^H and ^13^C NMR data of **278** apparently suggested the presence of Ileu instead of Val in **278**. However, detailed analysis of the ^1^H and ^13^C chemical shifts suggested the presence of L-*allo*-Ile instead of L-Ile. Although Marfey’s analysis was able to establish the absolute configurations of the amino acid residues, it was unable to distinguish between L-Ile and L-*allo*-Ile. Fortunately, a chiral-phase HPLC analysis of the acid hydrolysate of **278** was able to clarify that the amino acid was, in fact, L-*allo*-Ile. Thus, the complete structure of **278** was identified as *cyclo*-(*N*-Me-L-Tyr^1^-L-Phe-*N*-Me-Gly-L-Pro-*N*-Me-D-Tyr^2^-L-Leu-L-*allo*-Ile) [126].

The culture extract of *Talaromyces* sp. (CMB-TU011), isolated from an unidentified marine tunicate which was collected near Tweed Heads, NSW, Australia, furnished a hydroxamic acid-containing cyclic heptapeptide, talarolide A (**279**) (Figure 21). (+)-HRESI-MS and C_3_ and C_18_ Marfey’s analyses allowed the identification of six of the proposed amino acid residues as *N*-Me-L-Tyr, D-*allo*-Ile, *N*-Me-D-Ala, *N*-Me-D-Leu, L-Ala, and D-Ala, while NMR data revealed the presence of *N*-OH Gly residue. MS-MS fragmentations confirmed the amino acid sequence deduced by 2D NMR data, except for the location of L-Ala and D-Ala which was solved by C_3_ Marfey’s method. Thus, the structure of **279** was elucidated as *cyclo*-(*N*-Me-L-Tyr-*N*-Me-D-Ala-L-Ala-*N*-OH-Gly-*N*-Me-D-Leu-D-Ala-D-*allo*-Ile) [127].

The undescribed mortiamides A-D (**280**–**283**) (Figure 21) were isolated from the solid agar culture extract of a novel marine-derived fungus *Mortierella* sp. RKAG 110, isolated from a marine sediment at low tide at a depth of 30 cm in Frobisher Bay, Nunavut, Canada. The 1D and 2D NMR spectra, especially TOCSY experiments, were used to identify the amino acids, whereas HMBC and ROESY correlations were used to establish the amino acid sequences, which were confirmed by MS-MS fragmentation. The absolute configuration of the amino acids was determined by Mafrey’s method. When the peptides contained the D/L pair of amino acid such as **280** which contained L-Phe and D-Phe, their locations were determined by a comparison of the retention times from the LC-HRMS of the product obtained by the derivatization of a dipeptide fragment generated by partial hydrolysis with *N*-(5-fluoro-2,4-dinitrophenyl-5)-L-alaninamide (FDAA) with the retention times of the products of synthesized dipeptide standard derived in the same manner. Thus, the structures of **280**–**283** were elucidated as *cyclo*-(D-Phe-L-Phe-D-Val-D-Val-D-Val-L-Leu-D-Val), *cyclo*-(D-Phe-L-Phe-D-Val-D-Val-D-Val-L-Phe-D-Val), *cyclo*-(D-Ala-L-Ile-D-Ile-D-Val-D-Ile-L-Phe-D-Ile), and *cyclo*-(D-Leu-L-Phe-D-Ala-D-Ile-D-Ile-L-Phe-D-Val), respectively [128].

Unguisins A (**284**) and B (**285**) (Figure 21), two cyclic heptapeptides containing γ-aminobutyric acid (GABA), were obtained from the mycelia extract of a marine-derived fungus, *Emericella unguis*, which was isolated from a medusa*, Stomolopus meliagris,* collected in Venezuelan waters. The amino acid residues and GABA in both compounds were identified by FABMS, and 1D and 2D NMR analysis, while the amino acid sequences were established by an analysis of NOESY correlations. Marfey’s analysis was used to determine the absolute configuration of the amino acids. Consequently, the structures of **284** and **285** were elucidated as *cyclo*-(D-Val-D-Ala-D-Try-GABA-D-Ala-D-Val-L-Phe) and *cyclo*-(D-Val-D-Ala-D-Try-GABA-D-Ala-D-Val-L-Leu), respectively [129]. Compound **284** was also isolated from the chemically induced fermentation of a seaweed-derived fungus, *A. unguis* DLEP2008001 [130], and from the EtOAc extract of the deep-sea shrimp-associated fungus, *A. unguis* IV17-109 [39].

Further undescribed cycloheptapeptide incorporating GABA, unguisin E (**286**) (Figure 21), was isolated from the fermentation extract of *Aspergillus* sp. AF119, which was obtained from the soil collected in Xiamen Beach, China. The structure of **286** was quite similar to that of **284**. The only difference is the presence of β-Me-Phe in **286** instead of Phe in **284**. The sequence and relative stereochemistry of the amino acids were established by an analysis of NOESY correlations. Thus, the structure of **286** was elucidated as *cyclo*-(Ala-Trp-GABA-Ala-Val-β-Me-Phe-Val) [131].

A liquid culture extract of a marine-derived fungus, *Scytalidium* sp. CNC-310, isolated from a surface of a marine green alga *Halimedia* sp., which was collected from a patch reef at a depth of 15 m from the northern end of Long Island, the Bahamas, yielded two undescribed Aib-containing cyclic heptapeptides, scytalidamides A (**287**) and B (**288**) (Figure 21). The structures and sequences of amino acids were elucidated by detailed 1D and 2D NMR spectral analysis. The absolute configurations of the amino acid residues were determined by the advanced Marfey’s method. The structures of **287** and **288** were then established as *cyclo*-(L-Leu-L-Phe^1^-*N*-Me-L-Phe-L-Phe^2^-Aib-*N*-Me-L-Leu-L-Pro) and *cyclo*-(L-Leu-L-Phe^1^-*N*-Me-L-Phe-L-Phe^2^-Aib-*N*-Me-L-Leu-3-Me-L-Pro), respectively. The *trans* conformation of the Pro amide bond in **287** was determined by a small (3.5 ppm) differential value of ^13^C chemical shifts of C_β_ and Cγ (∆_βγ_), while the absolute stereochemistry of (2*S*,3*S*)-3-Me-Pro in **288** was determined by the advanced Marfey’s method [132].

The combined acetone and EtOAc extracts of a solid rice culture of *Aspergillus* sp. SCSIO 41501, isolated from the marine gorgonian *M. squamata* Nutting, collected from the South China Sea, furnished three undescribed cyclic lipopeptides, maribasins C–E (**289**–**291**) (Figure 21), together with the previously reported maribasins A (**292**), B (**293**), and marihysin A (**294**) (Figure 21). The structures of **289**–**291** were tentatively established as *cyclo*-(D-Pro-L-Gln-L-Asn-L-Ser-D-Asn-D-Tyr-D-Asn-D-β-aminoisohexadecanoic acid), *cyclo*-(D-Pro-L-Gln-L-Asn-L-Ser-D-Asn-D-Tyr-D-Asn-D-β-aminohexadecanoic acid), and *cyclo*-(D-Pro-L-Gln-L-Asn-L-Ser-D-Asn-D-Tyr-D-Asn-D-β-aminoanteisoheptadecanoic acid), respectively, by a combination of a detailed analysis of 1D and 2D NMR spectra, especially HMBC and NOESY correlations, and Marfey’s analysis [50].

#### 3.2.7. Cyclic Nonapeptides

**Figure 22:**
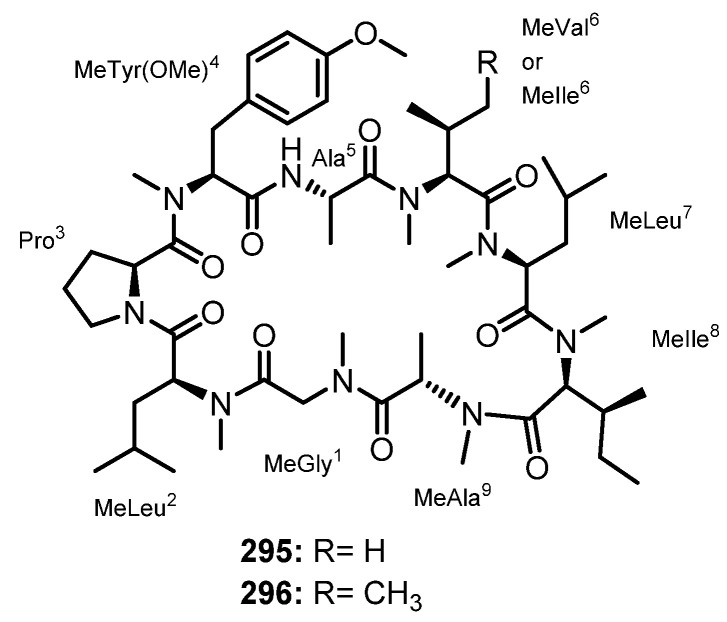
Structures of **295** and **296**.

Adachi et al. reported the isolation of two cyclic peptides containing highly *N*-methylated amino acids, clonostachysins A (**295**) and B (**296**) (Figure 22), from the EtOAc extract of a culture of a marine-derived fungus, *Clonostachys rogersoniana* (HJK9), obtained from a marine sponge *Halicondria japonica*, which was collected in Numazu, Japan. Extensive ^1^H and ^13^C NMR and 2D NMR spectral analysis, especially COSY, TOCSY, gHSQC, and gHMBC correlations, revealed the identity and the sequence of the amino acid residues in **295** and **296** as *N*-Me-Gly^1^-*N*-Me-Leu^2^-Pro^3^-*N*-Me-Tyr(OMe)^4^-Ala^5^-*N*-Me-Val^6^-*N*-Me-Leu^7^-*N*-Me-Ile^8^-*N*-Me-Ala^9^ and *N*-Me-Gly^1^-*N*-Me-Leu^2^-Pro^3^-*N*-Me-Tyr(OMe)^4^-Ala^5^-*N*-Me-Ile^6^-*N*-Me-Leu^7^-*N*-Me-Ile^8^-*N*-Me-Ala^9^, respectively. These sequences were further confirmed by LC-ESI MS/MS analyses. Marfey’s analysis revealed that all amino acids in **295** and **296** had the L-configuration. Moreover, *N*-Me-Ile was determined to be *N*-Me-L-Ile and not *N*-Me-L-*allo*-Ile, by a comparison of the HPLC retention time with those of the authentic samples [133].

#### 3.2.8. Cyclic Decapeptides

**Figure 23:**
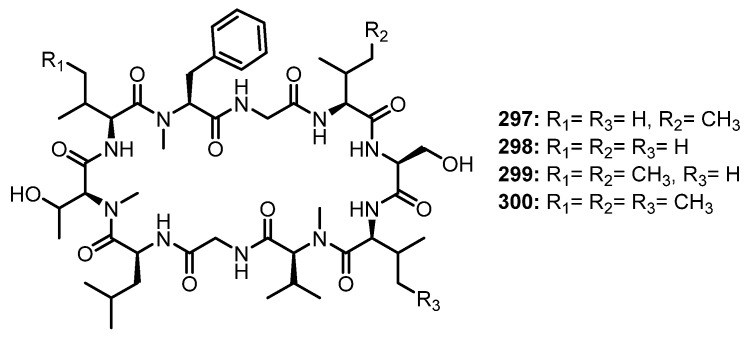
Structures of **297**–**300**.

The EtOAc extract of a culture of a marine-derived fungus, *Sesquicillium microsporum* RKAG 186, isolated from a marine sediment collected from the intertidal zone of Frobisher Bay, Nunavut, Canada, furnished four cyclic decapeptides, auyuittuqamides A–D (**297**–**300**) (Figure 23). The amino acid residues and their connectivity in these peptides were established by extensive 1D and 2D NMR spectral analysis, especially COSY, TOCSY, HSQC, HMBC, and ROESY correlations, while Marfey’s method revealed the L-configuration of all the amino acid residues in **297**–**300**, except for the *N*-Me-Thr residue whose commercial standard of *N*-Me-D-*allo*-Thr was not available. As the LC-HRMS using a reversed-phase C18 column could not separate L-Ile from L-*allo*-Ile, the configuration at the β-carbon remained unassigned. Thus, the structures of **297**–**300** were elucidated as *cyclo*-(*N*-Me-Thr-L-Val-*N*-Me-L-Phe-Gly-Ile-L-Ser-L-Val-*N*-Me-L-Val-Gly-Leu), *cyclo*-(*N*-Me-Thr-L-Val-*N*-Me-Phe-Gly-L-Val-L-Ser-L-Val-*N*-Me-L-Val-Gly-Leu), *cyclo*-(*N*-Me-Thr-Ile-*N*-Me-L-Phe-Gly-Ile-L-Ser-L-Val-*N*-Me-L-Val-Gly-Leu), and *cyclo*-(*N*-Me-Thr-Ile-*N*-Me-L-Phe-Gly-Ile-L-Ser-Ile-*N*-Me-L-Val-Gly-Leu), respectively [134].

### 3.3. Depsipeptides

Depsipetides are a group of peptides whose amide group is replaced by an ester group of the carboxylic acid of the amino acid [37].

#### 3.3.1. Linear Depsipeptides

**Figure 24:**
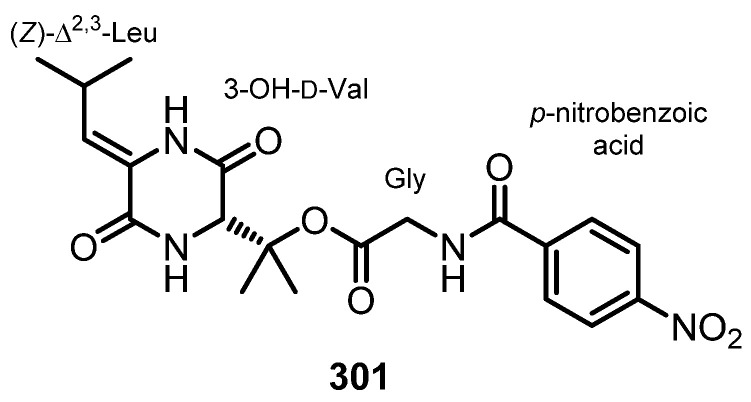
Structure of **301**.

The culture extract of a marine-derived fungus, *Aspergillus* sp. CMB-W031, isolated from an internal tissue of a mud dauber wasp *Sceliphron* sp., furnished a nitro depsitetrapeptide diketopiperazine, waspergillamide A (**301**) (Figure 24). Diagnostic ^1^H and ^13^C NMR signals, in combination with COSY and HMBC correlations, revealed the presence of Δ^2,3^-Leu, 3-OH-Val, Gly, and *p*-nitrobenzene residues. Marfey’s analysis revealed the D-configuration of the 3-OH-Val residue [135].

#### 3.3.2. Cyclic Depsipeptides

**Figure 25:**
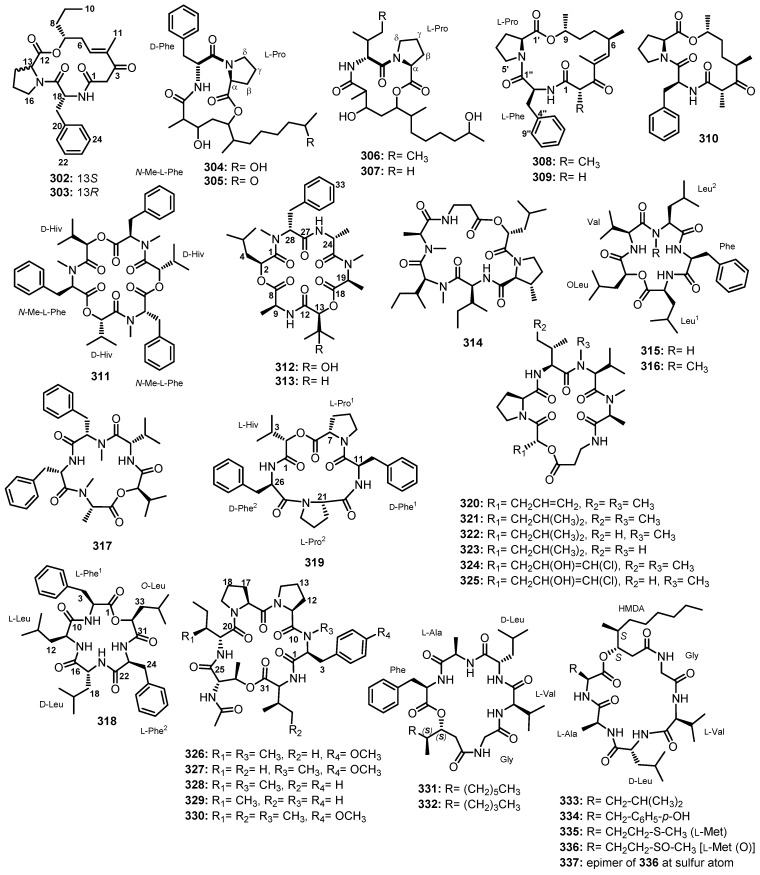
Structures of **302**–**337**.

The EtOAc extract of a culture of a marine-derived fungus, *Sarocladium kiliense* HDN11-112, obtained from the rhizosphere soil of the mangrove plant, *Avicennia marina*, furnished two depsipeptides, saroclides A (**302**) and B (**303**) (Figure 25). An analysis of the 1D and 2D NMR spectra suggested the presence of 7-hydroxy-4-methyl-3-oxo-dec-4-enoic acid (HMODA), in addition to Pro and Phe residues. Marfey’s analysis revealed the presence of D-Phe and L-Pro. The NOESY correlations between H_2_-6 and H_3_-11 established the *E* geometry of the double bond between C-4 and C-5 in HMODA. A single-crystal X-ray analysis of **302** established the absolute configurations of the stereogenic carbons as 7*R*,13*S*,18*R* and the *trans* conformations of the amid bonds between Pro and Phe [136].

An analysis of 1D and 2D NMR spectra revealed that **302** and **303** shared the same planar structure. However, Marfey’s analysis revealed the presence of D-Pro in **303** instead of L-Pro in **302**. NOESY correlations not only established that **303** has the same *E*-geometry of the double bond between C-4 and C-5 as in **302** but also deduced the same absolute configuration at C-7 as in **302**, i.e., 7*R*. Thus, **302** and **303** are a pair of epimers differing in the configuration of the Pro units, i.e., L-Pro and D-Pro, respectively. Interestingly, **302** was also isolated from the culture extract of *S. lamellicola* strain HDN13-430, which was obtained from a marine sediment collected in Antarctic Pritz Bay [136].

A chemical investigation of the mycelial extract of a solid fermentation of a marine-derived fungus, *Acremonium* sp. (MST-MF588a), isolated from an estuarine sediment sample collected from the Huon River near Franklin in Tasmania, Australia, led to the isolation of two major lipodepsipeptides, acremolides A (**304**) and B (**305**), together with two minor co-metabolites, acremolides C (**306**) and D (**307**) (Figure 25). A preliminary analysis of the ^1^H NMR (DMSO-*d_6_*) spectra of **304**–**307** revealed resonance doubling, at a ratio of 2:1, that coalesced at an elevated temperature, suggesting the presence of equilibrating isomers [137].

The NMR spectra of **304** (in DMSO-*d*_6_) exhibited not only the presence of both major and minor isomers but also the presence of Phe and Pro residues, which were confirmed as L-Pro and D-Phe by C_3_ Marfey’s analysis. A key gHMBC correlation between these residues (H_2_-5′ to C-1″) confirmed the amide linkage. Through ^13^C NMR chemical shift differences between C_β_ and C_γ_ of Pro, the equilibrating isomers of **304** were attributed to *cis* and *trans* prolinyl amide rotamers. An analysis of 1D and 2D NMR spectra revealed the presence of the L-Pro-D-Phe-NH dipeptide substructure and that the remaining structural feature of **304** was a substituted fatty acid which is attached to D-Phe by an amide bond. (+)-ESIMS analysis of a mild basic hydrolysis product of **304**, in combination a with NMR spectral analysis of the products obtained from the oxidation of **304** with pyridinium dichromate, revealed the structure of the fatty acid moiety. However, since the hydroxyl groups of the secondary alcohol of the hydroxy fatty acid portion did not react with the (*S*)-Mosher’s reagent, the absolute configurations of these stereogenic carbons could not be determined [137].

The HRESI-MS and 1D and 2D NMR analysis and C_3_ Marfey’s analysis of **305** revealed that its structure contained the same amino acid residues as **304**, i.e., L-Pro and D-Phe. The only difference is the presence of a ketone group at C-11, instead of an OH group, in the fatty acid portion. The value of ∆_βγ_ revealed the same 2:1 ratio of major *cis* versus minor *trans* rotamers as **304** [137].

The structures of **306** and **307** differ from those of **304** and **305** in that the D-Phe residue in **304** and **305** was replaced by an Ile and a Val residue, respectively. An analysis of Marfey’s 2,4-dinitrophenyl-5-L-alanine amide (DNP) derivatives obtained from **306** and **307** by C_3_ Marfey’s method unambiguously identified L-Pro and D-Ile, and L-Pro and D-Val, respectively. Moreover, in contrast to **304** and **305**, an analysis of the ^13^C NMR data of **306** and **307** confirmed a preference for major *trans* versus minor *cis* prolinyl amide bond conformers, suggesting that the replacement of the bulky D-Phe (in **304** and **305**) residue with either D-Ile (**306**) or D-Val (**307**) is responsible for an inversion of the preferred *cis/trans* balance in acremolide macrocycles [137].

The culture broth extract of the *Calcarisporium* sp. strain KF525, isolated from a water sample collected from the German Wadden Sea, yielded three undescribed cyclic depsipeptides, calcaripeptides A–C (**308**–**310**) (Figure 25). An analysis of HRESI-MS and 1D and 2D NMR data of **308** revealed the presence of a dipeptide consisting of the Pro and Phe residues, as well as a nonpeptidic portion that formed a 16-membered microcycle. The advanced Marfey’s analysis identified the configuration of Pro and Phe as L-Pro and L-Phe. Detailed analysis of the nonpeptidic portion revealed the presence of three secondary methyls, one tertiary methyl, one conjugated ketone carbonyl, and one *E*-double bond. The X-ray analysis allowed the determination of the relative configurations of **308**. Through a combination of the results of the advanced Marfey’s analysis and X-ray analysis, the absolute configurations of the stereogenic carbons in **308** were established as 2*S*,6*R*,9*R*,2′*S*,2″*S,* as well as the *cis* conformation of the Pro amide bond [138].

Compound **309**, whose NMR spectra were similar to those of **308**, was elucidated by a detailed analysis of HRESI-MS and 1D and 2D NMR data as an analog of **308**, but without the methyl group on C-2. The absolute configurations of its stereogenic carbons were not determined but were proposed to be the same as those in **308** [138].

On the other hand, the structure of **310** was very similar to that of **308** except for the lack of the *E*-double bond. Therefore, **310** consists of the L-Pro-L-Phe dipeptide and a nonpeptidic portion to form a 14-membered macrocycle. Like **309**, the absolute configurations of the stereogenic carbons in **310** were not determined but were proposed to be the same as those in **308** [138].

The EtOAc extract of a liquid culture of an endophytic fungus, *A. terreus* (no. GX7-3B), obtained from a branch of a mangrove tree, *Bruguiera gymnoihiza* (Linn.) Savigny, which was collected from the salt coastline of the South China Sea in Guangxi province, China, furnished the previously reported cyclic hexadepsipeptide, beauvericin (**311**) (Figure 25) [139]. The structure of **311** consists of three D-α-hydroxyisovaleryl and three *N*-Me-L-Phe residues in alternating sequence, i.e., *cyclo*-(D-α-hydroxyisovaleryl-*N*-Me-L-Phe)_3_ [140]. Compound **311** was also isolated from a culture extract of an endophytic fungus, *Fusarium* sp. (No.DZ27), obtained from the bark of a mangrove tree, *Kandelia cande* (L.) Druce, which was collected from the Dongzhai mangrove, Hainan, China [141].

Two undescribed cyclic depsipeptides, guangomides A (**312**) and B (**313**), together with homodestcardin (**314**), a member of the destruxin family (Figure 23), were isolated from the EtOAc extract of a liquid culture of an unidentified marine-derived fungus, strain No. 001314c, obtained from a yellow fan-shaped sponge, which was collected at the coast of Guango, Papua New Guinea. Detailed analysis of 1D and 2D NMR spectra and HRESI-MS data of **312** revealed the presence of 2-hydroxyisocaproic acid (Hic), three Ala residues, one Phe residue, and a 2,3-dihydroxyisovaleric acid (Hiv) subunit. The connectivity of the amino acid residues and two hydroxy carboxylic acids were established by HMBC correlations. The absolute configuration of *N*-Me-D-Phe was determined by Marfey’s method while the relative configurations of the stereogenic carbons were established by X-ray analysis. Through a combination of the absolute configuration of *N*-Me-D-Phe and the relative configurations from the X-ray data, the final absolute stereostructure of **312** was assigned as 2*S*,9*S*,13*S*,19*S*,24*R*,28*R* [142].

Since HRESI-MS data displayed the molecular formula of **313** with one oxygen atom less than that of **312**, and the ^1^H NMR showed diastereotopic H_3_-15 and H_3_-16 as doublets, it was suggested that Hiv in **312** was replaced by 2-hydroxyisovaleric acid, which was confirmed by 2D NMR analysis. The absolute configurations of the stereogenic carbons in **313** were proposed to be the same as those in **312** on the basis of biogenetic origin [142].

The ^1^H and ^13^C NMR spectra of **314** were very similar to that of homodestruxin B [143] and roseocardin [144]. Detailed analysis of the 2D NMR data of **314** revealed the presence of β-Me-Pro and *N*-Me-Leu residues instead of a Pro residue in homodestruxin B and *N*-Me-Val residue in roseocardin, respectively. Moreover, **314** and homodestruxin B displayed identical shifts of the chiral centers C-6, C-11, C-12, C-19, and C-20. On the other hand, the chiral centers C-26, C-27, and C-33 of **314** showed the same chemical shifts as the corresponding chiral centers of roseocardin. Moreover, the significant NOE observed between H-26 and H-28 was also consistent with these stereochemical proposal [142].

The mycelium extract of a marine-derived fungus, *Fusarium* sp., collected from the surface of a seagrass, *Halodule wrightii,* which was obtained in the inner lagoon of Little San Salvador Island, Bahamas, furnished a cyclic pentadepsipeptide, sansalvamide A (**315**) (Figure 25). Detailed interpretation of 1D and 2D NMR spectral data and HRESI-MS, revealed the presence of Val, Phe, two Leu, and leucic acid (*O*-Leu) residues. Chiral capillary GC analysis of the hydrolysate of **315** using the authentic standards for comparison established the L-configuration for the four amino acid residues in **315**. NOESY correlations between α-H of Leu^1^ and α-H of *O*-Leu suggested that both protons are on the same (α) face; therefore, the *O*-Leu residue also possesses the L-configuration [145].

The culture extract of *Fusarium* sp. CNL-619, isolated from a green alga, *Avrainvillea* sp., which was collected from a mangrove at a depth of 2 m in St. Thomas at Bovoni Cay, United States Virgin Islands, furnished *N*-methylsansalvamide (**316**) (Figure 25). The structure of **316** is closely related to **315**. An analysis of 1D-TOCSY data established the presence of Val, Leu, and Phe residues. Moreover, 1D-TOCSY, HMQC, and HMBC data further revealed the presence of one *N*-Me-Leu and one *O*-Leu residues. The amino acid sequence of the cyclic peptide was established by an analysis of HMBC correlations from the NH proton of the amino acid to the carbonyl carbon of the connecting amino acid. Marfey’s method revealed that all the amino acids in **316** have an L-configuration. The absolute configuration at C-2 in *O*-Leu was determined as 2*S* by the hydrolysis of **316,** followed by methylation to give a linear peptide with free hydroxyl group at C-2 for the preparation of Mosher’s ester [146].

An undescribed cyclic pentadepsipeptide, acremonamide (**317**) (Figure 25), was reported from a culture broth extract of a marine-derived fungus, *Acremonium* sp. CNQ-049, which was isolated from a sediment collected at a depth of 25 m from the coast of La Jolla, California. Detailed analysis of 1D and 2D NMR and HRESI-MS spectra led to the identification of *N*-Me-Phe, *N*-Me-Ala, Val, Phe, and Hiv. The stereochemistry of the four amino acids was determined as L-configured by the advanced Marfey’s method. The absolute configuration of the stereogenic carbon in Hiv was determined as *R* by GC-MS analysis, by a comparison of the retentime of the *O*-pentafluoropropionylated (−)-menthyl ester of Hiv in **317** with those of the the *O*-pentafluoropropionylated (−)-menthyl ester of the standard *S*-Hiv and *R*-Hiv [147].

An undescribed zygosporamide (**318**) (Figure 25) was isolated from the fermentation broth extract of a marine-derived fungus, *Zygosporium masonii* (CNK458), obtained from a marine cyanobacterium, collected off the Island of Maui, Hawaii. A combination of HRESI-MS data and 1D and 2D NMR spectral analysis revealed the presence of two Leu, two Phe, and one *O*-Leu residues. Marfey’s analysis indicated that all the amino acids in **318** have an L-configuration, except for one Leu residue which has a D-configuration. The absolute configuration at C-32 of *O*-Leu was determined as 32*S* by Mosher’s method of the linear peptide obtained by the hydrolysis of **318** [148].

Alternaramide (**319**) (Figure 25) was reported from the EtOAc culture extract of a marine-derived fungus, *Altenaria* sp. SF-5016, isolated from a sediment sample collected from the shoreline in the Masam Bay area, Korea. The 1D and 2D NMR spectral analysis revealed the presence of two Phe, two Pro, and one Hiv residues. Marfey’s analysis identified two D-Phe and two L-Pro residues. The absolute configuration at C-2 of Hiv was determined as 2*S* by Mosher’s method of a linear peptide with free OH group obtained from hydrolysis of **319**. Thus, the overall absolute configurations of **319** were established as 2*S*,7*S*,12*R*,21*S*,26*R* [149].

Boot et al. described the isolation of six previously reported cyclic depsipeptides belonging to the destruxin class, viz. destruxins A (**320**), B (**321**), B2 (**322**), desmethyldestruxin B (**323**), destruxin E chlorohydrin (**324**), and destruxin E2 chlorohydrin (**325**) (Figure 25) [150], from a culture extract of a marine sponge-associated fungus, *Metarrhizium* sp. (strain number 001103), obtained from a marine sponge, *Pseudoceratina purpurea* (coll. no. 00103), which was collected in Fiji [53].

Capon et al., in their search for new antiparasitic compounds, have isolated aspergillicins A–E (**326**–**330**) (Figure 25), depsipeptides that incorporated the unusual *N*-Me-L-Tyr-*O*-Me residue from the mycelia extract of *A. carneus* (MST-MF156), obtained from an estuarine sediment which was collected in Tasmania, Australia. The ^1^H and ^13^C NMR spectra of **326** indicated the presence of six amino acid residues, including two Pro, one Val, one Ile, and both *N*- and *O*-methylated Tyr. The degree of unsaturation revealed that **326** was a cyclic peptide. ESI-MS analysis of the mixture of amino acids obtained from acid hydrolysis of **326** confirmed the presence Pro, Val, Thr, Ile, and *N*-Me-Tyr. The assignment of the absolute stereochemistry of **326** was achieved by the advanced Marfey’s method. Thus, the structure of **326** comprises of L-Val, *N*-Ac-L-Thr, D-Ile, two L-Pro, and *N*-Me-L-Tyr-O-Me. In this case, the author has used the Marfey’s derivatives on a chiral HPLC column to distinguish between D-Ile and D-*allo*-Ile. Therefore, the complete structure of **326** was elucidated as *cyclo*-(*N*-Me-L-Tyr-*O*-Me-L-Pro-L-Pro-D-*allo*-Ile-*O*-Ac-L-Thr-L-Val). Moreover, the small values of the difference in ^13^C NMR shifts between C-12 and C-13, as well as between C-17 and C-18, confirmed the *trans* configuration of the amide bonds of the two Pro residues [71].

A Combination of the molecular formula, obtained by HRESI-MS, ^1^H NMR spectral analysis, ESI-MS analysis of the acid hydrolysate, and HPLC analysis of the Marfey’s derivatised acid hydrolysate confirmed that the structure of **327** differed from that of **326** by a replacement of the D-*allo*-Ile residue by a D-*nor*-Val residue. Compounds **328** and **329** were minor components and were isolated as a mixture. Despite this, an analysis of ^1^H NMR and ESIMS data proposed that **328** and **329** differed from **326** in the presence of the *N*-Me-L-Tyr-*O*-Me residue instead of the *N*-Me-L-Phe and L-Phe residues, respectively. The ^1^H NMR spectrum of the minor co-metabolite **330** was very similar to that of **326**. However, **330** showed more 14 atomic mass units than **326**. Marfey’s method with chiral HPLC analysis confirmed that Val in **326** was replaced by L-*allo*-Ile in **330** [71].

The mycelial extract of a marine-derived fungus, *Scopulariopsis brevicaulis* NCPF 2177, isolated from the inner tissue of a marine sponge, *Tethya aurantium*, which was collected in the Limski Fjord, Croatia, furnished two undescribed cyclohexadepsipeptides, scopularides A (**331**) and B (**332**) (Figure 25). Detailed analysis of HRESI-MS, 1D and 2D NMR spectra and HMBC correlations of **331** revealed the presence of Gly, Phe, Ala, Val, Leu, and 3-hydroxy-4-methyldecanoic acid (HMDA). The amino acid sequence could not be established unambiguously with NMR methods. However, with the aid of MS/MS fragment ions, the amino acid sequence was established as *cyclo*-(HMDA-Gly-Val-Leu-Ala-Phe). The configurations of the amino acid residues were established by the derivatization of the acid hydrolysis product of the peptide with *N*_α_-(2,4-dinitro-5-fluorophenyl)-L-valinamide (L-FDVA), and HPLC analysis of the derivatives. A comparison with the amino acid standards derived with L- and D-FDVA showed that the Val, Ala, and Phe residues all have the L-configuration, while Leu was D-configured. The absolute configuration of the stereogenic center C-3 of HMDA was established as 3*S* by Mosher’s method. Thus, the complete structure of **331** was elucidated as *cyclo*-[(*S*)-HMDA-Gly-L-Val-D-Leu-L-Ala-L-Phe)]. The HRMS and NMR data of **332** revealed that it had the same amino acid residues as **331**. However, it contains a 3-hydroxy-4-methyloctanoic acid (HMOA) instead of HMDA in **331** [151]. Compounds **331** and **332** were also reported from the EtOAc extract of the culture of *A. flavus*, isolated from the soft coral *Sarcophyton ehrenbergi*, collected from the Red Sea, Egypt [152].

Three undescribed cyclohexadepsipeptides, oryzamides A–C (**333**–**335**), were isolated, together with two isolation artifacts oryzamides D (**336**) and E (**337**), and **331** (Figure 25), from the mycelia extract of a marine sponge-associated fungus, *Nigrospora oryzae* PF18, which was obtained from a marine sponge, *Phakellia fusca*, collected from Yongxing Island. An analysis of HRESI-MS, 1D and 2D NMR spectral data of **333** led to the identification of one Ala, one Gly, one Val, two Leu residues, and HMDA. HMBC correlations allowed us to establish the amino acid sequence as Leu^1^-Ala-Leu^2^-Val-Gly-HMDA, which was further confirmed by the MS/MS fragment ion series. Given the degree of unsaturation, it was suggested that the Leu^1^ residue was connected to the HMDA moiety by an ester linkage. The absolute configuration of **333** was establish by a single-crystal X-ray diffraction analysis using Cu Kα radiation, allowing the assignments of the amino acid residues as L-Leu^1^, L-Ala, D-Leu^2^, and L-Val, as well as the configuration the stereogenic centers in the HMDA moiety as 3*S*,4*S*. Thus, the complete structure of **333** was elucidated as *cyclo*-[(L-Leu^1^-L-Ala-D-Leu^2^-L-Val-Gly-(3*S*,4*S*)-HMDA)] [153].

The ^1^H and ^13^C NMR spectra of **334** were similar to those of **333** with some differences in the aromatic region. An analysis of the 2D NMR spectra of **334** led to the conclusion that the Leu^1^ residue in **333** was replaced by the Tyr residue in **334**. HMBC correlations allowed us to establish the connectivity as *cyclo*-(Tyr-Ala-Leu-Val-Gly-HMDA). By using Marfey’s analysis, it was found that all the amino acid residues have the L configuration except Leu whose configuration is D. The absolute configurations of C-3 and C-4 of HMDA were proposed to be the same as those in **333** on the basis of biogenetic origin. Thus, the complete structure of **334** was elucidated as *cyclo*-[(L-Tyr-L-Ala-D-Leu-L-Val-Gly-(3*S*,4*S*)-HMDA)] [153].

An analysis of the HRESI-MS, 1D and 2D NMR spectra of **335** revealed that it contains Met instead of Leu^1^ residue in **333**. Thus, the planar structure of **335** was identified as *cyclo*-(Met-Ala-Leu-Val-Gly-HMDA), which was supported by the analysis of the ESIMS/MS fragments. The advanced Marfey’s method established the configurations of the amino acids as L-Met, L-Ala, D-Leu, and L-Val. Thus, the complete structure of **335** was elucidated as *cyclo*-[(L-Met-L-Ala-D-Leu-L-Val-Gly-(3*S*,4*S*)-HMDA] [153].

The two minor metabolites, **336** and **337**, are isomers as they showed the same molecular formula. Their molecular formula have one oxygen atom more than that of **335**. The ^1^H and ^13^C NMR spectra of **336** and **337** were almost identical to each other and very similar to those of **335**. A careful analysis of the 1D and 2D NMR data of **336** and **337** revealed that they are two diastereomers at a methionine sulfoxide residue resulting from oxidation of **335**. The absolute configurations of **336** and **337** were partially determined as L-Ala, L-Val, and D-Leu by the advanced Marfey’s method. In the case of the previously reported **331** whose stereocenter C-4 of the HMDA moiety had not been assigned for its absolute configuration [144], the X-ray analysis of **331** was able to establish the absolute configuration at C-4 of HMDA as 4*S* [153].

**Figure 26:**
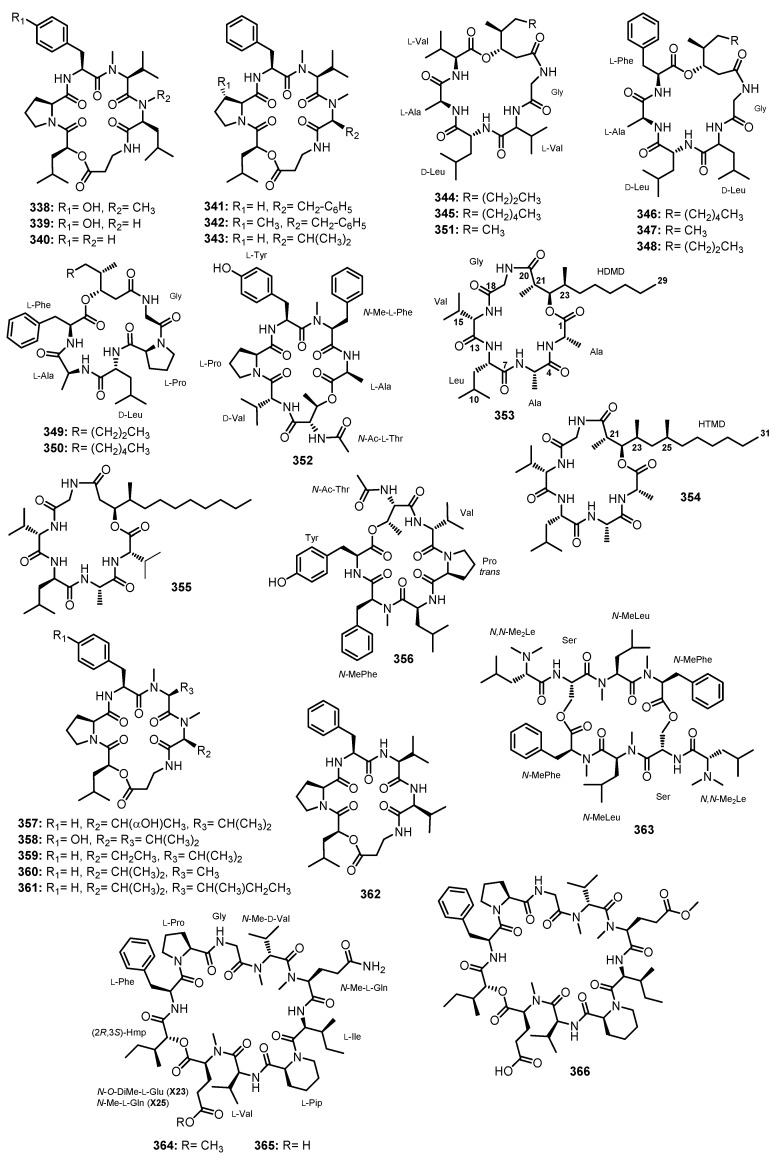
Structures of **338**–**366**.

The extract of a solid rice culture of *Beauveria feline* EN-135, isolated from an unidentified marine bryozoan, which was collected from Huiquan Gulf of the Yellow Sea, furnished three undescribed cyclohexadepsipeptides of the isaridin family, isaridin G (**338**), desmethylisaridin G (**339**), desmethylisaridin C1 (**340**) (Figure 26), together with three previously reported congeners, isaridins A (**341**), B (**342**), and E (**343**) (Figure 26) [154,155,156], all containing 2-hydroxy-4-methylpantanoic acid (HMPA) unit. An analysis of the HRESI-MS, ^1^H, and ^13^C NMR spectra revealed that **338** is a cyclic hexadepsipeptide with a Tyr residue. An analysis of COSY and HMBC correlations revealed the presence of HMPA, and five amino acid residues, *viz.* Pro, Tyr, *N*-Me-Val, *N*-Me-Leu, and β-Ala. The sequence of the amino acids was preliminarily established by observing HMBC correlations from the H-α and amide NH signals to the carbonyl of the connecting amino acid, as *cyclo*-(HMPA^1^-Pro^2^-Tyr^3^*-N*-Me-Val^4^-*N*-Me-Leu^5^-β-Ala^6^). Single-crystal X-ray diffraction using Cu Kα radiation established the *S* configuration for the HMPA^1^ and L configuration for all amino acid residues. Thus, the structure of **338** was established as *cyclo*-(*S*-HMPA^1^-L-Pro^2^-L-Tyr^3^*-N*-Me-L-Val^4^-*N*-Me-L-Leu^5^-L-β-Ala^6^) [157].

The molecular formula of **339**, determined by HRESI-MS, suggested that **399** might be a demethylated congener of **338**. Further interpretation of COSY, HSQC, and HMBC spectra confirmed the replacement of *N*-Me-Leu^5^ in **338** by a Leu^5^ residue in **339**. X-ray analysis of **339** confirmed the presence of *S*-HMPA and the L configuration of all the amino acid residues. Thus, the structure of **339** was elucidated as *cyclo*-(*S*-HMPA^1^-L-Pro^2^-L-Tyr^3^*-N*-Me-L-Val^4^-L-Leu^5^-L-β-Ala^6^) [157].

Detailed analysis of HRESI-MS and 1D and 2D NMR spectra of **340** identified its planar structure as *cyclo*-(HMPA^1^-Pro^2^-Phe^3^-*N*-Me-Val^4^-Leu^5^-β-Ala^6^). X-ray analysis of **340** established the *S* configuration for HMPA and the L-configuration for all amino acids. Therefore, the complete structure of **340** was established as *cyclo*-(*S*-HMPA^1^-L-Pro^2^-L-Phe^3^-*N*-Me-L-Val^4^-L-Leu^5^-β-L-Ala^6^). A conformation study of **338**–**340** using X-ray diffraction revealed that the HMPA^1^-Pro^2^ and *N*-Me-Val^4^-N-Me-Leu^5^ amide bonds in **338** showed *cis* conformations, while those in **339** and **340** showed *trans* conformations [157].

Hou et al., by using an integration of the LC-MS/MS-dependent molecular network and ^1^H NMR techniques, were able to target seven unreported cyclohexadepsipeptides, chrysogeamides A–G (**344**–**350**), in addition to the previously reported **331** and **332** [151], and nodupetide (**351**) [158] (Figure 26), from a solid rice-potato culture extract of an endophytic fungus, *P. chrysogenum* (CHSCLM-0003), isolated from a gorgonian coral, *Carijoa* sp. (GX-WZ-2010001), collected from Weihou coral reefs, South China Sea. An analysis of HRESI-MS, 1D and 2D NMR spectra of **344** led to the identification of one Gly, one Ala, one Leu, and two Val residues, in addition to a HMOA unit. HMBC correlations from the amide NH protons to their adjacent carbonyls established the connectivity of the amino acids in **344** as *cyclo*-(Val^2^-Ala-Leu-Val^1^-Gly-HMOA) and oxygen on C-24 should be part of an ester group between HMOA and Val^2^. Marfey’s analysis established the configuration of the amino acids as L-Ala, L-Val, and D-Leu. Thus, the structure of **344** was fully elucidated as *cyclo*-(L-Val^2^-L-Ala-D-Leu-L-Val^1^-Gly-HMOA) [159].

The NMR spectra of **345** were similar to those of **344**. However, a careful analysis of 1D and 2D NMR spectra of **345** revealed the presence of an HMDA moiety instead of HMOA. HMBC correlations, ESI-MS/MS, and Marfey’s method elucidated the full structure of **345** as *cyclo*-(L-Val-L-Ala-D-Leu-L-Val-Gly-HMDA) [159].

Detailed analysis of 1D and 2D spectra of **346** revealed the presence one Gly, two Leu, one Ala, one Phe residues, and HMDA. HMBC correlations, ESI-MS/MS, and Marfey’s method elucidated the full structure of **346** as *cyclo*-(L-Phe-L-Ala-D-Leu-D-Leu-Gly-HMDA) [159].

Compounds **347** and **349** were isolated as a 1:0.5 mixture. A combination of careful analysis of the ^1^H and ^13^C NMR data, molecular formula, HMBC correlations, and Marfey’s analysis confirmed the structures of **348** and **350** as *cyclo*-(L-Phe-L-Ala-D-Leu-D-Leu-Gly-HMOA) and *cyclo*-(L-Phe-L-Ala-D-Leu-L-Pro-Gly-HMDA), respectively [159].

FJ120DPA (**352**) (Figure 26) was also isolated from a semi-solid rice culture extract of a marine sediment-derived fungus, *A. ochraceopetaliformis.* Detailed analysis of HRESI-MS, 1D and 2D NMR spectra to identify the amino acids and HMBC correlations to establish the amino acid connectivity, as well as Marfey’s analysis, led to the elucidation of the structure of **352** as *cyclo*-(*N*-Me-L-Phe-L-Ala-Ac-L-Thr-D-Val-L-Pro-L-Tyr) [51].

The extract of a co-culture of a marine-derived fungus, *Emericella* sp. (strain CNL-878), isolated from the surface of a green alga *Halimeda* sp., collected at Madang Bay, in Papua New Guinea, and a marine actinomycete, *Salinispora arenicola* (strain CNH-665), isolated from a sediment sample collected from the Bahamas, resulted in the isolation of the unreported depsipeptides, emericellamides A (**353**) and B (**354**) (Figure 26) [160].

Detailed analysis of 1D and 2D NMR spectra including the HSQC, DEPT, COSY, and ^1^H-^1^H TOCSY of **353** led to the identification of one Gly, two Ala, one Val, one Leu, and 3-hydroxy-2,4-dimethyldecanoic acid (HDMD). HMBC and ROESY correlations allowed the determination of the connectivity of the amino acid sequence and the location of HDMD as *cyclo*-(Ala^1^-Ala^2^-Leu-Val-Gly-HDMD). Marfey’s analysis determined the L-configuration of the amino acids. The relative configurations of the stereogenic carbons of HDMD were determined by a *J*-based configurational analysis using ^3^*J*_HH_, ^3^*J*_CH_, and NOE correlations as (21R*,22R*,23S*). Mosher’s method determined the absolute configuration at C-22 as 22*R*. Therefore, the absolute configurations of the stereogenic carbons of HDMD were established as 21*R*,22*R*,23*S*. Thus, the complete structure of **353** was elucidated as *cyclo*-[L-Ala^1^-L-Ala^2^-L-Leu-L-Val-Gly-(21*R*,22*R*,23*S*)-HDMD] [160].

By using the same approach, **354** was found to contain the same amino acids as **353**, i.e., two L-Ala, one L-Val, and one L-Leu; however, the side chain was identified as 3-hydroxy-2,4,6-trimethyldodecanoic acid (HTMD). The relative configurations of C-21, C-22, and C-23 were proposed to be the same as those in **353**, i.e., 21R*,22R*,23S*, based on the high degree of similarity of their analogous ^1^H coupling constants and 1D NOE correlations. Since ROESY correlations between H-2 and H-22, and between 19-NH and H-22 in **354,** were similar to those of **353**, the authors suggested that it was highly indicative of the same absolute configuration at C-22 for both compounds. On the basis of consistent ROESY correlations, the absolute stereochemistry of HTMD was established as 21*R*,22*R*,23*S*,25*S*. It is worth mentioning that the marine actinomycete *S. arenicola* induced the culture of *Ermericella* sp. to produce ca. 100 times more than the single culture [160].

The solid rice culture extract of a soft coral-derived fungus, *A. sclerotiorum* SCSIO 41031, also furnished a lipodepsipeptide, scopularide I (**355**) (Figure 26). The HRESI-MS and 1D and 2D NMR analysis revealed the presence of one Val, two Ala, and one Leu residues, in addition to the 24-hydroxy-25-methyllauric acid (HMLA) moiety. The complete structure of **355** was confirmed by single-crystal X-ray analysis using Cu Kα radiation as *cyclo*-[L-Val-L-Ala^1^-D-Leu-L-Ala^2^-(24*S*,25*S*)-HMLA] [120].

An undescribed *N*-methylated depsipeptide, asperflosamide (**356**) (Figure 26), was also isolated from the fermentation extract of a marine sponge-associated fungus, *A. flocculosus* 16D-1. An analysis of the ^1^H, ^13^C NMR, COSY, HSQC, and TOCSY spectra of **356** revealed the presence of Tyr, *N*-Ac-Thr, Val, Pro, Leu, and *N*-Me-Phe residues. HMBC and ROESY correlations were used to determine the sequence of six amino acids as Tyr-*N*-Ac-Thr-Val-Pro-Leu-*N*-Me-Phe. Considering the degree of unsaturation, the cyclic nature of **356** was proposed, suggesting that the *N*-Ac-Thr residue was attached to the moiety by an ester bond. Thus, the planar structure of **356** was established as *cyclo*-(Tyr-*N*-Ac-Thr-Val-Pro-Leu-*N*-Me-Phe). The stereochemistry of the amino acid residues was determined as an L configuration by Marfey’s method. Therefore, the complete structure of **356** was assigned as *cyclo*-(L-Tyr-*N*-Ac-L-Thr-L-Val-L-Pro-L-Leu-*N*-Me-L-Phe). Moreover, the values of Δ*δ*_Cβ-Cγ_ of the L-Pro residue indicated a *trans* geometry for the Pro amide bond [113].

By integrating genomics and metabolomics approaches to discover new cyclopeptides from the EtOAc extract of a solid rice culture of a marine-derived fungus, *B. feline* SYSU-MS7908, isolated from a marine ascidian sample (*Styela plicata*) collected from the North Atoll of the Xisha Islands in the South China, Jiang et al. described the isolation of six undescribed cyclohexadepsipeptides of the isaridin family, isaridins I–N (**357**–**362**) (Figure 26), together with the previously reported isaridin E (**343**). The 1D and 2D NMR spectra, including COSY, NOESY HSQC, HMBC, and TOCSY, revealed the presence of HMPA in all the isolated compounds, in addition to common amino acids. The amino acid sequences were established by HMBC correlations from H-α or amide NH signals to the carbonyl of the connecting amino acid. The absolute configurations of the amino acid residues were determined by Marfey’s analysis while the absolute configuration of the stereogenic carbon (C-1α) of HMPA was established as *S* by an inspection of NOESY correlation. The absolute configuration of C-5α in *N*-Me-Thr in **357** was established as *S* by NOESY correlations and confirmed by a modified Mosher’s method [161].

In the case of **358**, the structure and absolute configurations were confirmed by X-ray analysis. Thus, the structures of **357**–**362** were elucidated as *cyclo*-[(*O*)-HMPA^1^-L-Pro^2^-L-Phe^3^-*N*Me-L-Val^4^-*N*Me-L-Thr^5^-*β*-Ala^6^ (CO)], *cyclo*-[(*O*)-HMPA^1^-L-Pro^2^-L-Tyr^3^-*N*Me-Val^4^-*N*Me-Val^5^-*β*-Ala^6^ (CO)], *cyclo*-[(*O*)-(1α*S*)-HMPA^1^-L-Pro^2^-L-Phe^3^-*N*Me-L-Val^4^-(5α*S*)-*N*Me-Abu^5^-*β*-Ala^6^ (CO)], *cyclo*-[(*O*)-(1α*S*)-HMPA^1^-L-Pro^2^-L-Phe^3^-*N*Me-L-Ala^4^-(5α*S*)-*N*Me-Val^5^-*β*-Ala^6^ (CO)], *cyclo*-[(*O*)-(1α*S*)-HMPA^1^-L-Pro^2^-L-Phe^3^-*N*Me-L-Ile^4^-*N*Me-Val^5^-*β*-Ala^6^ (CO)], and *cyclo*-[(*O*)-(1α*S*)-HMPA^1^-L-Pro^2^-L-Phe^3^-L-Val^4^-L-Val^5^-*β*-Ala^6^ (CO)], respectively [161].

The mycelium extract of *Clonostachys* sp. ESNA-A009, isolated from an unidentified marine sponge collected in Japan, afforded IB-01212 (**363**), a cyclodepsipeptide featuring *C*_2_ symmetry (Figure 26). The symmetrical dimeric nature of **363** was based on the fact that its molecular formula has twice the number of the carbons and protons observed in the NMR spectra. An analysis of the COSY, gHSQC, and gHMBC spectra led to the identification of the natural amino acid Ser, and the unnatural *N*-Me-Leu, *N*,*N*-Me_2_-Leu, and *N*-Me-Phe, while gHMBC experiments were used to unequivocally establish the amino acid sequence and the site of dimerization. The stereochemistry of Ser, *N*-Me-Leu, and *N*-Me-Phe was determined by Marfey’s method, which confirmed the L configuration for all of them. The configuration of *N*,*N*-Me_2_-Leu was determined by derivatization of the hydrolyzate of **363** with 1-mental, and we compared its HPLC retention times with those of the derivatized standards. In this way, the configuration of *N*,*N*-Me_2_-Leu was established as L. Moreover, the structure of **363** was also confirmed by a comparison of its NMR data and HPLC retention times with those of the synthetic counterpart [162].

The undescribed cyclodecadepsipeptides, phaeosphamides A (**364**) and B (**365**), were isolated together with a previously reported congener Sch 217048 (**366**) (Figure 26) [163], from the extract of the solid rice culture of the fungus, *Phaeosphaeriopsis* sp. Esf-30, isolated from a rhizosphere sediment of a mangrove plant, *Bruguiera gymnorhiza*, which was collected from Techeng Isle, Zhanjiang, Guangdong Province, China [164].

An analysis of the 1D and 2D NMR spectra of **364** revealed the presence of nine proteinogenic amino acids, viz. Phe, Pro, Gly, *N*-Me Val, *N*-Me Gln, Ile, Pip, Val, and *N*-*O*-dimethylglutamic acid (*N*-*O*-DiMe Glu), in addition to 2-hydroxy-3-methylpentanoic acid (HMP). The amino acid sequence in **364** was established by HMBC and NOESY correlations as *cyclo*-(*N-O*-DiMe-Glu-Val-Pip-Ile-*N*-Me-Gln-*N*-Me-Val-Gly-Pro-Phe-HMP). Therefore, the structure of **364** differs from **366** only the presence of *N-O*-DiMe Glu in **364** instead of *N-*Me Glu in **366**. The absolute stereochemistry of **364** was determined by a comparison of its ^1^H NMR spectra and optical rotation with those of the methylated **366**. Since, the ^1^H NMR spectra and optical rotations of **364** and the methylated **366** are nearly identical, it was concluded that they are the same compound. Therefore, the complete structure of **364** was elucidated as *cyclo*-(*N*-*O*-DiMe L-Glu-L-Val-L-Pip-L-Ile-*N*-Me L-Gln-*N*-Me D-Val-Gly-L-Pro-L-Phe-(2*R*,3*S*)-HMP) [164].

An analysis of the 1D and 2D NMR spectra of **365** revealed the presence of *N*-Me-Glu instead of *N*-Me-Gln in **366**. The amino acid sequence of **365** was identical to that of **366** on the basis of HMBC and NOESY correlations. The absolute configurations of the amino acids were determined by the advanced Marfey’s method. Therefore, the complete structure of **365** was established as *cyclo*-(*N*-Me L-Glu-L-Val-L-Pip-L-Ile-*N-O*-DiMe L-Glu-NMe D-Val-Gly-L-Pro-L-Phe-(2*R*,3*S*)-Hmp) [164].

## 4. Biological and Pharmacological Activities

Marine-derived peptides are another group of marine natural products that exhibit a vast array of biological and pharmacological activities. For practical purposes and to facilitate the readers, the activities are classified as follows:

### 4.1. Cytotoxic Activity

Compounds **1**, **2** (Figure 3)**, 8** (Figure 4), and **29** (Figure 5), isolated from the culture broth extract of a deep-sea-derived fungus, *S. obclavatum* EIODSF 020, were assayed against human leukemia cell lines, HL-60 ((human acute promyelocytic leukemia) and K562 (chronic myelogenous leukemia cells). Compounds **1** and **29** exhibited weak cytotoxicity against HL-60 cells, with IC_50_ values of 100 and 64.7 μM, respectively, while **2** and **8** showed cytotoxicity toward K562 cells with IC_50_ values of 73.5 and 39.4 μM, respectively [38].

Compounds **17**–**19** (Figure 4), isolated from a mangrove endophytic fungus, *Aspergillus terreus* LM.5.2, showed weak cytotoxicity against human cancer cell lines, such as DLD-1 (colorectal adenocarcinoma), MCF-7 (breast adenocarcinoma), and PC-3 (prostatic adenocarcinoma), with IC_50_ values ranging from 58.3 to 96.8 μM, in the MTT [3-(4,5-dimethylthiazol-2-yl)-2,5-diphenyl tetrazolium bromide] assay. Moreover, **17**–**19** exhibited cytotoxic activity toward rat non-malignant cardiomyocyte H9c2 cells, with IC_50_ higher than 76.7 μM [43].

Compounds **27** and **28** (Figure 4), isolated from a marine sediment-derived fungus, *A. terreus* FA009, were examined for their potential as anticancer agents or gene expression modulators by exploring their DNA-binding properties by fluorescence spectroscopy. The results showed that the fluorescence of **27** decreased upon adding double-stranded DNA oligomers. Based on the observed stoichiometry, **27** appeared to have a combined binding mode, i.e., intercalation by **27** along with groove binding by the peptide backbone [48].

Compounds **39**, **40** (Figure 6) and **57** (Figure 10), isolated from the marine sponge- associated *Acremonium* sp., were tested for the in vitro anticancer activity. Compounds **39** and **40** mildly inhibited the growth of the murine L1210 (lymphocytic leukemia) cancer cell line in a disk diffusion soft agar colony-forming assay, with relative potencies of 4 and 1. Compound **57** showed potent cytotoxic activity against L1210 cell line with relative potency of 3300 and against HCT-116 (human colorectal carcinoma) cell line, with IC_50_ value of 3.5 ng/mL [52].

Compounds **41**, **42** (Figure 6), and **57**–**61** (Figure 10), isolated from a marine sponge- associated fungus, *Acremonium* sp. 021172, were subject to the in vitro soft agar disk diffusion screening assay on various cancer cell lines, such as NCl-H125 (human non-small-cell lung carcinoma), HCT-116, CEM (human lymphocytic leukemia), C38 (murine colon adenocarcinoma), and CFU-GM (murine bone marrow). However, only **57**, **58**, and **61** exhibited similar cytotoxicity against the NCl-H125 cancer cell line, with similar IC_50_ values of 1.3 nM, suggesting that the methyl groups on R_1_, R_3_, and R_4_ have little impact on their cytotoxicity. A colonogenic study indicated that a therapeutic effect of **57** against NCl-H125 cells would require a chronic exposure (168 h) at a concentration of 2.0 ng/mL or higher, or a bolus exposure (24 h) at a concentration of 300 ng/mL or higher [53].

Compounds **62**–**66** (Figure 10), isolated from a marine sediment-derived fungus, *Trichoderma* sp. MMS1255, were evaluated for cytotoxic activity against KB (*human epithelial carcinoma)* cell line, using the MTT assay. All the tested compounds showed strong cytotoxicity, with IC_50_ values of 2.4, 4.3, 0.8, 0.8, and 0.7 μM, respectively. The positive control, alamethicin F50/5, showed an IC_50_ of 9 μM [58].

Compounds **67**–**73** (Figure 11), isolated from a marine sponge-associated fungus, *Trichoderma* sp. GXIMD 01001, displayed cytotoxicity against four human cancer cell lines, including A549 (lung adenocarcinoma), H1299 (non-small cell lung cancer), SW480 (colorectal cancer), and SW1990 (pancreatic adenocarcinoma), with IC_50_ values ranging from 0.46 to 4.7 μM. The positive control, cisplatin, showed IC_50_ values between 6.2 and 34 μM. Furthermore, the most potent compound, **72**, showed antiproliferative activity against SW480 cells and deformation of the colony. Compound **72** was found to induce apoptosis in SW480 cancer cells [59].

Compounds **82** and **83** (Figure 13), isolated from the culture broth extract of a marine-derived fungus, *Aspergillus* sp., showed significant antiproliferative activity against a panel of cancer cell lines, viz. RKO (colon carcinoma), SNU638 (gastric carcinoma), SK-HEP-1 (hepatic adenocarcinoma), and MDA-MB (breast cancer), with IC_50 (72h)_ values of 0.8, 4.8, 2.9, and 7.0 μM for **82**, and 1.1, 8.0, 3.5, and 9.7 μM for **83**, respectively. The positive control, etoposide, showed the IC_50_ values of 3.3, 0.3, 0.4, and 10.1 μM, respectively [62].

Compounds **84** and **85** (Figure 13), isolated from a marine fish-possessing-derived fungus, *P. fellutanum* Biourge, showed potent cytotoxicity against three cancer cell lines, i.e., murine leukemia P388 (IC_50_ values of 0.2 and 0.1 μg/mL, respectively), L1210 (IC_50_ values of 0.8 and 0.7 μg/mL, respectively), and KB (IC_50_ values of 0.5 and 0.7 μg/mL, respectively) [63].

Compound **93** (Figure 14), isolated from a neomycin-resistant mutant of a marine sediment-associated fungus, *P. purpurogenum* G59, selectively inhibited the growth of HeLa (human cervical carcinoma) cells with an inhibition rate of 39.4% at a concentration of 100 μg/mL, which was comparable with the positive control, 5-fluorouracil (41.4% inhibition at a concentration of 100 μg/mL) [68].

Compound **109** (Figure 14), isolated from a marine-derived fungus, *Microsporum* sp., displayed cytotoxic activity against HeLa cells. Furthermore, **109** induced apoptosis in HeLa cells through the downregulation of Bcl-2 expression, upregulation of Bax expression, and activation of the caspase-3 and p53 pathways [74].

Compound **117** (Figure 14), isolated from a marine-derived fungus, *Aspergillus* sp. SF-5044, exhibited weak cytotoxic activity against HL-60, MDA-MB-231 (triple-negative human breast cancer adenocarcinoma), Hep3B (hepatocellular carcinoma), 3Y1 (rat fibroblast), and K562, with IC_50_ values of 75, 130, 150, 180, and 250 μM, respectively [80].

Compounds **134**–**137** (Figure 14), isolated from a marine cyanobacterium-associated fungus, *E. rostratum* (Drechsler) CNK-630, exhibited in vitro cytotoxicity against HCT-116 cell line, with IC_50_ values of 8.5, 1.9, 0.76, and 16.5 μg/mL (9, 4.4, 1.6, and 35 μM), respectively. The authors suggested that the increased potency of **135** when compared to **136** might be due to the ketone functionality at C-5 (C-5′); however, the reduced potency in **137** was not in agreement with this simplistic analysis [85].

Compound **139** (Figure 14), isolated from a marine sediment-derived *Penicillium* sp. WF-06, was shown to inhibit the growth of HepG2 (hepatoblastoma) cell line, with an IC_50_ value of 19.6 μmol/L [86].

Compound (+)-**148** (Figure 15), isolated from the marine-derived fungus, *A. versicolor* HBU-7, showed cytotoxicity against HGC-27 (*human gastric carcinoma*) cell line, with an IC_50_ value of 4.54 μM. The positive control, cisplatin, showed IC_50_ = 8.63 μM [90].

Compounds **156**, **159**, and **160** (Figure 15), isolated from a semi-mangrove endophytic fungus, *E. rubrum* G2, were assayed against seven human cancer cell lines, including MCF-7, SW1990, HepG2, NCl-H460 (non-small-cell lung carcinoma), SMMC-7721 (hepatocarcinoma), HeLa, and Du145 (prostatic carcinoma). Compound **156** selectively inhibited the growth of the SMMC-7721 cell line with IC_50_ = 30 μg/mL. Compound **159** showed cytotoxicity against HepG2, NCI-H460, and HeLa cell lines with IC_50_ values of 20, 22, and 20 μg/mL, respectively, while **160** was active against MCF-7, SW1990, SMMC-7721, and HeLa cells with IC_50_ = 20, 20, 20, and 30 μg/mL, respectively [91].

Compounds **169** and **170** (Figure 15), isolated from the tunicate-derived fungus, *Aspergillus* sp. DY001, were evaluated for cytotoxicity, by MTT assay, against MDA-MB-231, HCT-116, and HeLa. Compounds **169** and **170** showed cytotoxicity against MDA-MB-231 and HCT-116 cell lines, with IC_50_ values of 24.3 and 15.1 μM (for **169**) and 26.3 and 16.2 μM (for **170**), but were inactive against HeLa cells. The IC_50_ values of the positive control, 5-FU, were 13.0 and 4.6 μM, against MB-231 and HCT-116 cells, respectively [93].

Compounds **190** and **191** (Figure 16), isolated from a marine sediment-derived fungus, *Eurotium* sp. SCSIO F452, exhibited moderate cytotoxicity against the SF-268 (human glioblastoma) cell line, with IC_50_ values of 12.5 and 15.0 μM, respectively, and weak cytotoxicity against the HepG2 cancer cell line, with IC_50_ values of 30.1 and 37.3 μM, respectively. The positive control, taxol, showed IC_50_ values of 6.0 and 11.1 μM toward SF-268 and HepG2, respectively [100].

Compound **192** (Figure 17), isolated from a brown alga-derived fungus, *Aspergillus* sp. (BM-05 and BM-05ML), was assessed, by a microplate MTT assay, for the anti-proliferative activity against four human tumor cell lines, including K562, HCT-116, A2780 (ovarian cancer), and A2780CisR (cisplatin-resistant mutant in ovarian cancer). Compound **192** showed cytotoxicity against all the tested cell lines, with IC_50_ values of 67.8, 28.5, 27.3, and 49.4 μM, respectively. The positive control, cisplatin, showed IC_50_ values of 7.8, 33.4, 0.8, and 8.4 μM, respectively [81].

Compounds **92**, **115** (Figure 14), **197**, **201,** and **210** (Figure 17), isolated from a sea water-derived fungus, *A. ochraceopetaliformis* DSW-2, showed weak cytotoxicity against HPAC (pancreatic ductal adenocarcinoma) and BxPC3 (pancreatic carcinoma) cell lines, with IC_50_ values higher than 20 μM [78].

Compound **221** (Figure 18), isolated from a marine gorgonian-associated *A. terreus* SCSGAF0162, was tested for its cytotoxicity against U937 (*pro-monocytic, human myeloid leukaemia*), K562, BGC823 (gastric carcinoma), MOLT-4 (acute lymphoblastic leukemia), MCF-7, and A549 cell lines by the MTT method. However, **221** only showed cytotoxicity against the U937 and MOLT-4 cell lines, with IC_50_ = 6.4 and 6.2 μM, respectively. Taxol was used as a positive control against the U937, K562, BGC-823, MOLT-4, Mcf7, and A549 cell lines, with IC_50_ values of 1.9, 4.9, 3.5, 1.8, 5.0, and 3.6 nM, respectively [108].

Compounds **223** and **224** (Figure 18), isolated from a marine bryozoan-associated fungus, *Microsporum* cf. *gypseum*, were assayed for in vitro cytotoxicity against human HCT-116 cancer cell line. Compound **223** (IC_50_ = of 0.6 μg/mL) exhibited better cytotoxicity than **224** (IC_50_ = of 8.5 μg/mL) toward HCT-116, suggesting the importance of the ketone carbonyl group in the side chain for biological activity. Compound **223** also showed cytotoxicity against a 60-cell line panel of National Cancer Institute (NCI), with the mean IC_50_ = 2.7 μg/mL. Etoposide and DMSO (solvent) were used as positive and negative controls, respectively [110].

Compound **227** (Figure 19), isolated from a marine-derived fungus, *A. versicolor* (MST-MF495), exhibited weak cytotoxicity toward some human tumor cell lines, including MM418c5 (malignant melanoma), DU145, and T47D (breast cancer cell line resistant to tamoxifen), with the median effective concentration (EC_50_) values of 66, 90, and 94 μg/mL, respectively [112].

Compound **245** (Figure 20), isolated from a marine gorgonian coral-derived fungus, *A. versicolor* (TA01-14), at a concentration of 10 μM, showed weak cytotoxicity toward human NCI-H292 (pulmonary carcinoma) and A-431 (skin squamous carcinoma) cell lines, by the MTT assay, with an inhibition ratio of 53.84% and 63.6%, respectively. The reference drug, adriamycin, at a concentration of 1 μM, showed inhibition of 93.4% and 91.0%, respectively [117].

Compound **256** (Figure 20), isolated from a marine-derived halotolerant fungus, *A. sclerotiorum* PT06-1, displayed weak growth inhibition of the HL-60 cell line, with an IC_50_ value of 56.1 μg/mL [119].

Compound **260** (Figure 20), isolated from a marine sponge-associated fungus, *A. similanensis* KUFA0013, showed weak cytotoxicity against MCF-7, NCl-H460, and A373 (melanoma), in the sulforhodamine B (SRB) assay, with GI_50_ values of 125, 117, and 115, respectively. Doxorubicin, a positive control, showed GI_50_ for MCF-7 = 42.8 nM; MDA-MB-231 = 10.86 nM; NCI-H460 = 94.0 nM; SF-268 = 94.0; and UACC-62 = 94.0 nM [121].

Compounds **276**–**278** (Figure 21), isolated from a marine sediment-derived *A. persicinum* SCSIO115, displayed cytotoxicity, in a SRB assay, against SF-268 (anaplastic gliblastoma), MCF-7, and NCI-H460, with IC_50_ values of 3.7, 3.0, and 11.6 μM (for **276**), 45.6, 82.7, and >100 μM (for **277**), and 3.2, 2.7, and 4.5 μM (for **278**), respectively. The IC_50_ values of cisplatin, a positive control, were 4.6, 10.2, and 1.6 μM [126].

Compounds **287** and **288** (Figure 21), isolated from a marine alga-derived fungus, *Scytalidium* sp. CNC-310, exhibited moderate cytotoxic activity against HCT-116 cells, with IC_50_ = 2.7 and 11.0 μM, respectively. In the NCI 60 cell-line panel, both compounds showed the mean GI_50_ values of 7.9 and 4.1 μM, respectively. Interestingly, the most sensitive cell lines were MOLT-4 (IC_50_ = 3.0 μM for **287**) and UACC-257 (melanoma) (IC_50_ = 1.2 μM for **288**) [132].

Compound **311** (Figure 25), isolated from the culture extract of a mangrove endophytic fungus, *A. terrus* (no. GX7-3B), exhibited cytotoxicity against MCF-7, A549, HeLa, and KB, with IC_50_ values of 2.02, 0.82, 1.14, and 1.10 μM, respectively. The IC_50_ values of epirubicin, the positive control, were 1.07, 0.79, 0.42, and 0.05 μM, respectively [140]. Compound **311** also inhibited the growth of KB and KBv200 (multidrug-resistant KB) cell lines, with IC_50_ values of 5.76 and 5.34 μM, respectively. Further cell cycle studies revealed that the mitochondrial pathway, such as the reduction of reactive oxygen species (ROS) production, a decrease of mitochondrial membrane functionality, the release of cytochrome c, caspases-9 and -3 activation, and poly (ADP-ribose) polymerase (PARP) cleavage, are involved in the apoptosis induction. On the other hand, the regulation of either Bcl-2 or Bax was not involved in the apoptosis induction by **311** in the KB and KBv200 cell lines [141].

Compound **315** (Figure 25), isolated from a mycelia extract of a marine-derived *Fusarium* sp., showed the in vitro selective cytotoxicity against COLO 205 (colon cancer) and SK-MEL-2 (melanoma) cell lines, with IC_50_ values of 3.5 and 5.9 μg/mL, respectively. The positive control, mitomycin C, showed IC_50_ = 5.3 μg/mL against both cancer cell lines. Interestingly, **315** exhibited IC_50_ = 9.8 μg/mL against HCT-116 cell line, while the corresponding linear peptide was inactive, suggesting that the cyclization is vital for cytotoxic activity [145]. Compounds **315** and **316** (Figure 25) also exhibited moderate cytotoxicity, with mean GI_50_ values of 8.3 and 3.6 μM, respectively, in the NCI’s human tumor cell line screen [146].

Compound **318** (Figure 25), isolated from a marine cyanobacterium-associated fungus, *Z. masonii* (CNK458), showed the median GI_50_ value of 9.1 μM in the cytotoxicity assay against 60 cancer cell lines in the NCI. Interestingly, **318** exhibited a selective cytotoxic activity toward SF-268 and RXF 393 (renal cancer) cell lines, with GI_50_ values of 6.5 and ≤5.0 nM, respectively [148].

Compounds **320**–**325** (Figure 25), isolated from a marine sponge-associated fungus, *Metarrhizium* sp., were tested against H125, HCT-116, and CEM, as well as C38 (mouse colon carcinoma) and mouse CFU-GM (colony-forming unit for granulocytes and macrophages), using a soft agar disk diffusion method. Compounds **324** and **325** selectively inhibited the murine C38 cells at the lowest concentration of 0.136 and 0.150 nM, respectively, but no inhibition of CFU-GM cells was observed. Compounds **320**, **322**–**325** inhibited all the solid tumors, with **324** having the best inhibitory activity at a concentration of 34 μM. Compound **324** showed an IC_50_ value of 160 nM against HCT-116 cells. The colonogenic study of **324** indicated that only a chronic exposure (168 h) of 190 ng/mL or higher would display the therapeutic effect toward HCT-116 cells. The maximum tolerated dose in mice (MTD) for **324** was established as 0.125 mg/mouse (6.25 mg/kg) [53].

Compounds **331** and **332** (Figure 25), isolated from a marine sponge-associated fungus, *S. brevicaulis* NCPF 2177, showed significant inhibition on growth of Colo357 (metastatic pancreatic adenocarcinoma), Panc89 (pancreatic ductal adenocarcinoma), and HT29 (colorectal adenocarcinoma) cell lines at a final concentration of 10 μg/mL. The cell viability of Colo357, Panc89, and HT29 was decreased by 36%, 42%, and 37% (for **331**) and 26%, 49%, and 24% (for **332**), respectively [151].

Compounds **331** and **332** (Figure 25), isolated from a gorgonian coral-derived fungus, *P. chrysogenum* (CHSCLM-0003), were also examined for their cytotoxicity against MCF-7 by the MTT method, and BEL-7402 (*human hepatocellular carcinoma)*, HepG2, and SMMC7721 (*human hepatocellular carcinoma*) by the SRB method. Compounds **331** and **332** exhibited strong and selective cytotoxic activity against HepG2 and SMMC7721, with IC_50_ values of 26.81 and 33.75 μM, respectively. The IC_50_ values of the positive controls, 5-FU, were >100 and 15.79 μM, and of cisplatin were 28.27 and 21.81 μM [159].

Compounds **353** and **354** (Figure 26), isolated from a co-culture of the algicolous fungus, *Emericella* sp. (strain CNL-878), and a marine actinomycete, *S. arenicola* (strain CNH-665), displayed weak cytotoxicity against HCT-116, with IC_50_ values of 23 and 40 μM, respectively [160].

Compound **355** (Figure 26), isolated from a soft coral-associated fungus, *A. sclerotiorum* SCSIO 41031, showed cytotoxicity against human nasopharyngeal carcinoma cell lines, HONE1 and HONE1-EBV, with IC_50_ values of 13.0 and 10.1 μM, respectively [120].

Compound **356** (Figure 26), isolated from a marine sponge-associated fungus, *Clonostachys* sp. ESNA-A009, was assayed against a panel of 14 human tumor cell lines. However, **356** showed the most potent cytotoxicity toward LN-caP (prostate cancer), SK-BR3 (breast cancer), HT29, and HeLa cell lines, with the same growth inhibition (GI_50_) of 10^−8^ M [162].

Compound **364** (Figure 26), isolated from the sediment-derived fungus, *Phaeosphaeriopsis* sp. Esf-30, was evaluated, by the MTT method, against five cancer cell lines, viz. AGS (human gastric adenocarcinoma), BEL-7402 (human hepatocarcinoma), HepG2, BIU87 (human bladder cancer), and B16 (mouse melanoma). Compound **364** exhibited selective cytotoxicity against AGS cells at 20 μM, which was equivalent to the positive control, docetaxel, at 10 μM. Furthermore, the IC_50_ value of **364** against the proliferation of AGS cells was 5.14 μM. Flow cytometry analysis showed that **364** treatment caused AGS cell arrest in the G2 phase in a dose-dependent manner, leading to the increase in apoptotic induction at the concentration ranging from 2.5 to 10 μM [164].

### 4.2. Antibacterial and Antibiofilm Activities

Most of marine organisms biosynthesize a unique and distinct class of peptides with antibacterial and antimicrobial properties against a broad spectrum of Gram-positive and Gram-negative bacteria, which are known as antimicrobial peptides (AMPs). However, these peptides are also able to inhibit the detrimental fungi, viruses, and parasites with the lowest possibility of developing resistance [165]. The majority of AMPs are produced ribosomally and then undergo post-translational modification, whereas some of the AMPs are biosynthesized by a multiple-enzyme system [9].

The antibacterial activity of **39**, **40** (Figure 6), and **57** (Figure 10), isolated from the marine sponge-associated *Acremonium* sp., were assayed against *Staphylococcus epidermis*. Compounds **39** and **57** were active and exhibited minimum inhibition concentration (MIC) values ranging from 25–80 μg/mL, while **40** showed no antibacterial activity. The positive control, vancomycin, showed MIC = 0.63 μg/mL [52].

Compounds **49** (Figure 8) and **55** (Figure 9), isolated from a culture extract of the marine tunicate-associated *Talaromyces* sp. (CMB-TU011), showed strong antibacterial activity against a Gram-positive bacterium, *Bacillus subtilis* ATCC 6633, with IC_50_ = 1.5 and 3.7 μM, respectively. Rifampicin was used as a positive control (40 mg/mL in 10% DMSO) [55].

Compounds **53** and **54** (Figure 8), isolated from the marine microalgal-derived fungus, *Tolypocladium* sp., were evaluated against ten Gram-negative, seven Gram-positive, and two acid-fast bacterial strains, by a broth microdilution antibacterial assay. Both compounds selectively showed weak to moderate antibacterial activity against six Gram-positive bacteria, including *B. subtilis*, methicillin-susceptible *S. aureus* (MSSA), methicillin-resistant *S. aureus* (MRSA), *Listeria ivanovii*, *Enterococcus faecium*, and *E. faecalis* (MIC values ranging from 4 to 32 μM), and against two acid-fast bacteria, i.e., *Mycobacterium tuberculosis* and *M. smegmatis* (MIC values ranging from 20 to 80 μM) [57].

Compounds **62**, **64**, and **66** (Figure 10), isolated from a marine sediment-derived fungus, *Trichoderma* sp. MMS1255, exhibited the same antibacterial activity against *S. aureus* (CIP53.156), with MIC value of 25 μg/mL. Gentamycin (100% inhibition of *S. aureus* at 40 μg/mL in 1% MeOH) was used as a positive control [58].

Compounds **67**–**73** (Figure 11), obtained from a sponge-associated fungus, *Trichoderma* sp. GXIMD 01001, exhibited weak and moderate antibacterial activity against *S. aureus*, with MIC values between 23 and 45 μg/mL. The positive control, chloromycetin, showed MIC = 3.1 μg/mL [59].

Compound **86** (Figure 14), isolated from a marine sponge-associated fungus, *Penicillium* sp. F37, inhibited a biofilm formation of the pathogenic *S. epidermidis* ATCC 35984 in a range of 0.25, 0.5, and 1.0 mg/mL (60%, 65%, and 85%, respectively) without interference on bacterial growth, whereas, at 2.0 mg/mL, it was capable of inhibiting bacterial growth, and, at 0.125 mg/mL, no significant inhibition of a biofilm formation was observed [64].

Compound **92** (Figure 14), isolated from a marine sediment-derived fungus, *P. chrysogenum* DXY-1, at a sub-minimum inhibitory concentration, decreased the production of quorum sensing (QS)-regulated violacein of *Chromobacterium violaceum* by 79%, and the production of QS-mediated pyocyanin, proteases, and elastase activities of *P. aeruginosa* PA01 by 41%, 20%, and 32%, respectively. Moreover, **92** also destroyed the biofilm formation and decreased the QS gene expression of *P. aeruginosa* PA01. A molecular docking study showed that **92** blocked the effects of QS autoinducers via competitive binding to the same pocket of the receptor proteins [67].

Compounds **108** and **110** (Figure 14), isolated from a marine sponge-associated *E. chevalieri* MUT2316, were assayed for antibacterial activity against marine bacteria. Compound **108** showed inhibitory activity against *Halomonas aquamarina* ATCC 14400 and *Vibrio aesturianus* ATCC 35048, with the same low observable effect concentration (LOEC) value of 0.001 μg/mL, while **110** inhibited the growth of *Pseudoalteromonas citera* ATCC 29720 and *Polaribacter irgensii* ATCC 700398, with LOEC values of 0.01 and 1 μg/mL, respectively. Compound **110** also inhibited the adhesion of only two bacterial strains, *P. citera* and *Roseobacter littoralis* ATCC 49566, with LOEC values of 0.001 and 0.1 μg/mL, respectively [73].

Compound **145** (Figure 15), isolated from a marine sediment-derived fungus, *A. versicolor* (MF030), displayed significant antibacterial activity against Bacille Calmette-Guerin (BCG) with an MIC value of 6.25 μg/mL, which is modest when compared to the positive control, isoniazid (MIC value of 0.05 μg/mL), but no significant antibacterial activity against the Gram-positive bacteria *S. aureus* ATCC 6538 and *B. subtilis* ATCC 6633, the Gram-negative bacteria *P. aeruginosa* (PAO1) and *Escherichia coli* (ATCC 25922), or yeast, *Candida albicans* (SC 5314) [89].

Compound **226** (Figure 19), isolated from a marine gorgonian-associated fungus, *Aspergillus* sp. XS-20090B15, showed antibacterial activity toward *B. cereus* and *S. epidermis* with the same MIC value of 12.5 μM. Ciprofloxacin was used as a positive control [46].

Compounds **129** (Figure 14) and **229** (Figure 19), isolated from an unidentified decaying green alga-derived fungus, *Asteromyces cruciatus* 763, were assayed for their antibacterial activity. Compound **229** showed weak antibacterial activity against *B. subtilis* and *S. epidermidis* with growth inhibition of 61% and 30%, at a concentration of 100 μM. Compound **129**, at a concentration of 50 μg level, exhibited total inhibition of 2.5 and 7 mm against *E. coli* and *B. megaterium* in the agar diffusion assay [83].

Compounds **169** and **170** (Figure 15), isolated from the tunicate-associated fungus, *Aspergillus* sp. DY001, was evaluated for antibacterial activity by a disc diffusion assay, at a concentration of 50 μg/disc, against two pathogens, including *E. coli* ATCC 25922 and *S. aureus* ATCC 25923. Both compounds moderately inhibited the bacterial strains with inhibition zones ranging from 16 to 22 mm, and MIC values ranging from 4 to 8 μM. The positive control, ciprofloxacin, showed inhibition zones of 30 and 22 mm, respectively [93].

Compounds **175** and **178**–**182** (Figure 16), isolated from a deep-sea cold seep-derived fungus, *A. chevalieri* CS-122, were evaluated against two human pathogenic bacteria, *E. coli* and *Micrococcus luteus*, and three aquatic pathogenic bacteria, *Vibrio harveyi*, *Edwardsiella tarda*, and *Aeromonas hydrophilia*, by a serial dilution assay using 96-well microtiter plates. Compounds **179** and **182** moderately inhibited all pathogens, with MIC values of 16–32 μg/mL, while **178**, **180**, and **181** selectively showed antibacterial activity against *E. coli* and *V. harveyi*, with MIC values ranging from 4 to 32 μg/mL. Compound **175** inhibited the growth of *A. hydrophilia* and *E. coli* with MIC values of 4 and 8 μg/mL, respectively. Chloramphenicol was used as a positive control, showing MIC values ranging from 2 to 8 μg/mL. Given the significant antibacterial activity of **175** against *A. hydrophilia* (MIC = 4 μg/mL), a further antibacterial mechanism was assessed using scanning electron microscope (SEM) before and after treatment with **175.** In addition to obvious cell deformation and serious damage to the cell membrane, **175** also caused deep grooves and clear pores on the bacterial surface leading to sufficient bacteriolysis and death of *A. hydrophilia* [96].

Compound **256** (Figure 20), isolated from a marine-derived halotolerant fungus, *A. sclerotiorum* PT06-1, selectively inhibited the growth of *P. aeruginosa* with an MIC value of 35.3 μM [119].

Compounds **269**–**271** (Figure 21), isolated from the coral-associated *A. versicolor* (CHNSCLM-0063), displayed antibacterial activity against *Mycobacterium marinum*, with MIC values of 23.4, 81.2, and 87.5 μM, respectively. The positive controls, rifampin, streptomycin, and isoniazid, showed MIC values of 19.0, 20.1, and 88.5 μM, respectively. In a further assay against *M. tuberculosis* H37Rv, only **270** showed a weak activity, with MIC = 100 μM. Compared to **270**, it was suggested that the hydroxyl group at the R_1_ position of **269** considerably reduces the anti-*M. tuberculosis* activity [124].

Compounds **272** and **273** (Figure 21), also isolated from the marine gorgonian coral-derived *A. versicolor* (CHNSCLM-0063), showed anti-*M. tuberculosis* H37Rv activity, with MIC values of 50 and 100 μM, respectively. The MIC values of the positive controls, rifampin and isoniazid, were 0.00625 and 0.0125 μM, respectively [125].

Compounds **284** and **285** (Figure 21), isolated from a marine-derived fungus, *E. unguis*, showed moderate antibacterial activity against, *S. aureus* [129]. Compound **284**, also inhibited the aquatic pathogen, *Vibrio parahemolyticus*, with an inhibition zone of 9.0 mm, which was the same as the positive control, ampicillin [130].

Compounds **312** and **313** (Figure 25), isolated from a sponge-associated fungal strain No. 001314c, exhibited weak antibacterial activity against *S. epidermis* ATTC 12228 and *Enterococcus durans* ATTC 11576, in a micro broth dilution assay, with the same MIC value of 100 μg/mL [144].

Compound **319** (Figure 25), isolated from a marine sediment-derived fungus, *Altenaria* sp. SF-5016, showed antibacterial activity against *B. subtilis* KCTC1021 and *S. aureus* KCTC1928, with inhibition zones of 8 and 13 mm, respectively. The positive control, gentamicin, showed inhibition zones of 16 and 13 mm, respectively [149].

Compounds **338**–**340**, and **343** (Figure 26), isolated form a marine bryozoan-associated fungus, *B. feline* EN-135, showed antibacterial activity, in the disk diffusion and double-dilution methods, against *E. coli* with MIC values of 64, 64, 8, and 16 μg/mL, respectively. The MIC value of chloramphenicol, the positive control, was 4 μg/mL [157].

Compounds **353** and **354** (Figure 26), isolated from a co-culture of an algicolous fungus, *Emericella* sp. (strain CNL-878), and a marine actinomycete, *S. arenicola* (strain CNH-665), showed moderate antibacterial activity against *S. aureus* MRSA, with MIC = 3.8 and 6.0 μM, respectively [160].

### 4.3. Antifungal Activity

Compounds **20** and **21** (Figure 4), isolated from a marine-derived fungus, *T. purpureogenus* CX11, were assayed for their antifungal activity against phytopathogenic fungus, *Fusarium oxysporum* ATCC 28856, and human pathogenic fungi *Candida albicans* SC5314 and *Cryptococcus neoformans* GOYJ11988. The MIC values against *F. oxysporum* ranged from 12.5 to 25 μg/mL, and against the two remaining fungi ranged from 40 to 80 μg/mL [44].

Compound **129** (Figure 14), isolated from an unidentified decaying green alga-derived fungus, *A. cruciatus* 763, showed antifungal activity at a concentration of 50 μg, against *Mycotypha microspora*, *Euritium rubrum*, and *Microbotryum violaceum*, with total inhibition zones of 13.5, 4, and 13 mm, in the agar diffusion assay. The positive control, miconazole exhibited total inhibition zones of 6–10 mm [83].

Compounds **169** and **170** (Figure 15), isolated from a tunicate-associated fungus, *Aspergillus* sp. DY001, was evaluated for antifungal activity against *C. albicans* ATCC 14053, using a disc diffusion assay at a concentration of 50 μg/disc. Both compounds weakly inhibited the growth of *C. albican*s, with inhibition zones of 11 and 12 mm, and exhibited the same MIC values of 16 μM. The inhibition zone and MIC value of ketoconazole, the reference drug, were 30 mm and 0.26 μM [93].

Compounds **196**, **197**, **201**, **204**, and **207** (Figure 17), isolated from a marine halotolerant fungus, *A. sclerotiorum* PT06-1, selectively inhibited the growth of *C. albicans*, in an agar dilution method, with MIC values of 7.5, 3.8, 30, 6.7, and 30 μM, respectively [102].

Compound **219** (Figure 18), isolated from a co-culture of mangrove endophytic fungi, *Phomopsis* sp. K38 and *Altenaria* sp. E55, displayed antifungal activity against four crop-threatening fungi, *viz. Gaeumannomyces graminis*, *Rhizoctonia cerealis*, *Helminthosporium sativum*, and *Fusarium graminearum*, with MIC values of 220, 160, 130, and 250 μg/mL, respectively. The positive control, tridimefon, showed MIC values of 150, 100, 120, and 150 μg/mL, respectively [105].

Compounds **246**, **250**, and **251** (Figure 20), isolated from a deep-sea-derived fungus, *S. obclavatum* EIODSF 020, exhibited significant antifungal activity against *A. versicolor* and *Curvularia australiensis*, with MIC values of 0.625, and 0.156 μM for **246**, 0.625, and 0.156 μM for **250**, and 1.562, and 0.156 μM for **251**, respectively. The MIC values of the positive controls, ketoconazole and amphotericin, were 0.625, 0.156 μM against *A. versicolor*, and 1.562 and 0.156 μM against *C. australiensis*, respectively [49].

Compounds **246**, **250**–**253** (Figure 20), isolated from a marine sediment-derived fungus, *S. obclavatum* EIODSF 020, showed antifungal activity against phytopahogenic fungi, *Altenaria solani* and *Colletotricum asianum,* with MIC values ranging from 0.195 to 6.250 μg/disc, and mild antifungal activity against *Pyricullaria oryzae*. The positive controls, amphotericin B and ketoconazole, showed MIC values of 6.250, 0.195, 25 μg/disc, and 12.5, 25 and 100 μg/disc, respectively, against *A. solani*, *C. asianum and P. oryzae* [118].

Compounds **255** and **256** (Figure 20), isolated from a marine-derived halotolerant fungus, *A. sclerotiorum* PT06-1, exhibited moderate antifungal activity, in an agar dilution method, toward *C. albicans* with MIC values of 7.0 and 3.5 μM, respectively [119].

Compounds **289**–**293** (Figure 21), isolated from a marine gorgonian-associated fungus, *Aspergillus* sp. SCSIO 41501, showed antifungal activiy against phytopathogenic fungal strains, *Fusarium oxysporum*, *C. australiensis*, *P. oryzae*, *A. solani*, and *Colletotrichum gloeosporioiles,* with MIC values ranging from 3.12 to 50 μg/disc. The MIC of the reference drug, actidione, ranged between 6.25 and 12.5 μg/disc. The difference in the β-amino fatty acid side chain can effectively influence the antifungal activity of these cyclic lipopeptides [50].

Compounds **358** and **360** (Figure 26), isolated from a marine-derived fungus, *B. feline* SYSU-MS7908, showed significant inhibition of the mycelial growth of *Geotrichum citri-aurantii,* with EC_50_ values of 56.8, and ca. 90.0 μg/mL, in the agar dilution method. The positive control, triadimefon, showed EC_50_ values of 146.4 μg/mL. In order to understand the effect of **358**, the morphology and membrane permeability of *G. citri-aurantii* caused by **358**, at a concentration of 60 μg/mL, were examined by SEM which showed a morphology change through the spare growth, collapse, deformation, peeling, and shrinkage of the mycelia of *G. citri-aurantii.* Additionally, treatment with a 60 μg/mL concentration disturbed its cell membrane integrity [161].

### 4.4. Antiviral Activity

Compounds **74**–**78** (Figure 12), isolated from a marine-derived fungus, *Scytalidium* sp., were assayed against herpes simplex virus type 1 and type 2 (HSV-1 and HSV-2). Compounds **74**–**78** were added to Vero cells infected with HSV-1 for one hour and the virus-induced cytopathic effects were measured for 5 days. Compounds **74**–**78** showed ED_50_ values of 1.1, 3.5, 2.2, 2.0, and 3.1 μM, respectively. The experiments showed that the antiviral efficacy of **74** is greater when it is incubated with HSV-1 prior to the exposure to Vero cells. In a standard plaque reduction assay, **74** also inhibited the replication of both HSV-1 and HSV-2 with the same ED_50_ values of 280 nM [60].

Compound **221** (Figure 18), isolated from a marine gorgonian-derived *A. terreus* SCSGAF0162, showed antiviral activity against the influenza virus strains A/WSN/33 (H1N1) and A/Hong Kong/8/68 (H3N2), with IC_50_ values of 15 and 8.1 μM, respectively. RIBA was used as a positive control against H1N1 and H3N2, showing IC_50_ values of 20.2 and 0.41 μM, respectively [108].

Compounds **10** (Figure 4) and **235** (Figure 19), isolated from a gorgonian-derived fungus, *Aspergillus* sp. SCSIO 41501, were assayed against HSV-1 using a plaque reduction assay. Compounds **10** and **235** exhibited antiviral activity, under their non-cytotoxic concentration (TC_0_), toward a Vero cells, with IC_50_ = 19.8 and 9.5 μM, respectively. The TC_0_ and TC_50_ values against Vero cells were 153.2 and 346.0 μM (for **10**), and 81.9 and 204.4 μM (for **235**). The positive control, acyclovir, showed the IC_50_ and TC_0_ values of 3.0 and >1000 μM, respectively. Interestingly, **235** displayed, at a concentration of 12.5 μM, antiviral activity against acyclovir-resistant clinical isolates of HSV-1-106 and HSV-1-153, with about a 50% inhibition rate [41].

Compounds **246**, **250**, and **251** (Figure 20), isolated from a deep-sea-derived fungus, *S. obclavatum* EIODSF 020, showed, under their non-cytotoxic concentration (TC_0_) toward a Vero cell, antiviral activity against HSV-1 in a plaque reduction assay, with IC_50_ values of 14.0, 16.7, and 15.6 μM, respectively. The TC_0_ values were 25.1, 57.2, and 49.4 μM, respectively. The IC_50_ and TC_0_ values of the positive control, acyclovir, were 3.0 and >1000 μM, respectively [49].

Compounds **261**–**265** (Figure 20), isolated from the marine sediment-derived fungus, *A. persicinum* SCSIO115, showed antiviral activity against HSV-1, with EC_50_ values of 16, 8.7, 27, 24, and 14 μM, respectively. The EC_50_ value of the positive control, ganciclovir, was 0.025 μM. Compounds **261**, **262**, and **265** act through an inhibition of UL42 gene transcription in HSV, which is an essential polypeptide for viral DNA replication [122].

### 4.5. Antiparasite Activity

Compound **356** (Figure 26), isolated from a marine sponge-associated *Clonostachys* sp., showed leishmanicidal activity at a low micromolar range of concentrations on promastigote and amastigote forms of the parasite. The mechanism of action of **356** to kill *Leishmania* is through an apoptosis-like process which involves intracellular targets [166].

### 4.6. Anti-Dinoflagellate Activity

Dinoflagellates are the most important contributors to the harmful algal bloom (red tide). Compounds **295** and **296** (Figure 20), isolated from a sponge-associated fungus, *Clonostachys rogersoniana* (HJK9), caused an inhibition of the motility of a dinoplagellate, *Prorocentrum micans*, at a concentration of 30 μM [133].

### 4.7. Algicidal Activity

The algicidal activity of **108**–**110** (Figure 14), isolated from a marine sponge-associated fungus, *E. chevalieri* MUT2316, was investigated against marine microalgae including *Cylindrotheca closterium* (AC170), *Exanthemachrysis gayraliae* (AC15), *Halamphora coffeaeformis* (AC713), *Phaeodactylum tricornutum* (AC171), and *Prophyridium purpureum* (AC122). Interestingly, **108** inhibited the growth and adhesion of all microalgae with LOEC values ranging from 0.001 to 100 μg/mL. Compound **109** inhibited the growth of *C. closterium*, *H. coffeaeformis*, and *P. tricornutum*, with LOEC values of 0.001, 0.01, and 1 μg/mL, respectively, and inhibited the adhesion of *C. closterium*, *E. gayraliae*, *H. coffeaeformis*, and *P. tricornutum*, with LOEC values of 1, 0.01, 1, and 0.001 μg/mL, respectively. Compound **110** only showed inhibitory activity against the growth of *H. coffeaeformis* and *P. purpureum*, with LOEC values of 0.1 and 0.001 μg/mL, respectively. The LOEC values of **110** against the adhesion of *E. gayraliae* and *P. tricornutum* were the same, i.e., 0.01 μg/mL [73].

Compound **234** (Figure 19), isolated from a marine sediment-derived fungus, *Aspergillus* sp. SCSIOW2, exhibited algicidal activity against two strains of noxious red tide algae, *Akashiwo sanguinea* and *Chattonella marina*. Compound **234** showed high 2 h inhibition rates of 58% and 36%, at a concentration of 50 μM, respectively. The authors found that **234** inhibited the growth of examined algae cells through a significant increase in ROS level, which was caused by the damage of superoxide dismutase (SOD) activity, and, consequently, generated high malondialdehyde (MDA) levels [114].

### 4.8. Enzyme Inhibitory Activity

#### 4.8.1. Inhibition of Indoleamine 2,3-Dioxygenase (IDO) Activity

Indoleamine 2,3-dioxygenase (IDO) catalyzes the cleavage of the 2,3-indole bond of L-Trp, converting it to *N*-formylkynurenine in the first and rate-limiting step of the kynurenine catabolic pathway. IDO-expressing cancer cells use this transformation to reduce Trp concentrations in their microenvironments to levels that prevent T-lymphocyte activation and proliferation, thus rendering the ability of tumors to escape the T-lymphocyte-based immune response. A growing body of clinical data shows that many primary tumor cell lines obtained from patients overexpress IDO. Therefore, IDO has emerged as a promising molecular target for the development of a new class of therapeutic agents for treating cancer that work by modulating an extrinsic property of tumor cells [85].

Compounds **184**–**186** (Figure 16), isolated from a marine sediment-derived fungus, *P. cucumerina*, inhibited the purified, recombinant human IDO activity in vitro with IC_50_ values of ca. 2 μM. It was suggested that some portion of the phenoxazinone fragment C represents a new IDO inhibitory pharmacophore [98].

#### 4.8.2. Inhibition of Acethylcholinesterase (AChE) Activity

Compound **311** (Figure 25), isolated from a mangrove endophytic fungus, *A. terreus* (no. GX7-3B), showed anti-AChE activity with an IC_50_ value of 3.09 μM. The positive control, huperazine A, had IC_50_ = 0.003 μM [140].

Compound **355** (Figure 26), isolated from a soft coral-associated fungus, *A. sclerotiorum* SCSIO41031, inhibited the activity of AChE with IC_50_ = 15.6 μM. A molecular docking study in the 2D model showed that the alkyl chain of **355** formed a hydrophobic interaction with the active site residues such as Trp84, Asp72, Try70, and Trp279. Moreover, the NH proton of Gly formed a hydrogen bond with the active site residue Tyr334 [120].

#### 4.8.3. Inhibition of Histone Deacethylase Activity

Histone deacetylases (HDACs) comprise a family of enzymes that catalyze the removal of acetyl groups from lysine residues of histones and mediate chromatin remodeling and gene expression. Due to the capacity of HDAC inhibitors to suppress cell proliferation in a variety of transformed cells in culture and in tumor-bearing animals, they have great promise as new cancer drugs [167].

Compound **223** (Figure 18), isolated from a marine bryozoan-associated fungus, *Microsporum* cf. *gypseum*, showed stronger inhibitory activity against a mixture of both histone deacethylases, (HDACs) and HDAC8, than suberoylaniline hydroxamic acid (SAHA), a known HDAC inhibitor antitumor agent, with IC_50_ values of 0.14 and 0.55 μM (for **223**) and 0.3 and 0.78 μM (for SAHA), respectively [110].

#### 4.8.4. Inhibition of Sortase A (SrtA) and Isocitrate Lyase (ICL) Activities

Sortase A (SrtA) is an important enzyme found in the membrane of *S. aureus*, which is directly related to the virulence of bacteria. By inhibiting SrtA activity, promising antibacterial agents can be expected. Compounds **18** and **19** (Figure 4), isolated from a mangrove endophytic fungus, *Aspergillus terreus* LM.5.2, at a concentration of 80 μM, inhibited Srt A activity by more than 20%, while **17** (Figure 4) was void of activity [43].

Compounds **38** (Figure 5), **230**, **231** (Figure 19), and **252** (Figure 26), isolated from marine-derived fungi, *A. allahabadii* and *A. ochraceopetaliformis*, were assayed against the key anti-virulence targets in pathogenic micro-organisms such as SrtA and isocitrate lyase (ICL). Compounds **38, 230**, **231**, and **252** exhibited moderate inhibition of *S. aureus* SrtA with IC_50_ values of 77.0, 70.0, 53.1, and 131.9 μM, respectively. The positive controls, berberine chloride and curcumin, showed the IC_50_ values 104.3 and 47.8 μM, respectively. Compound **231** was further assayed for *C. albicans* ICL and showed an inhibition of ICL with an IC_50_ = 116.8 μM. The 3-Nitropropanoic acid, the positive control for ICL, showed IC_50-_ = 18.5 μM [51].

#### 4.8.5. Inhibition of Pancreatic Lipase (PL) Activity

The enzyme pancreatic lipase (PL) hydrolyzes triglyceride so that it could be absorbed by human intestines. Therefore, PL inhibitors can prevent hyperlipidemia and obesity by decreasing the absorption of triglycerides [168].

Compounds **79**–**81** (Figure 13), isolated from a marine sponge-associated fungus, *A. flavipes* 164013, were found to potently inhibit PL activity, with IC_50_ values of 0.23, 0.07, and 0.14 μM, respectively. The positive control, kaempferol, showed IC_50_ = 1.5 μM. The position of chlorine was important in the bioactivity since **80** (2-chlorinated) was more active than **81** (4-chlorinated) and **79** (2,4-dichlorinated) [61].

Compounds **266**–**268** (Figure 20), isolated from a marine sponge-associated fungus, *Aspergillus* sp. 151304, showed a significant and dose-dependent inhibition of PL activity, with IC_50_ values of 7.6, 1.8, and 0.5 μM, respectively. Although kinetic studies revealed noncompetitive inhibition of PL by **268**, the molecular dynamics simulation showed that **268** could bind PL at the entrance of the catalytic pocket. The corresponding positive controls, isoginkhetin and orlistat, had the IC_50_ values of 2.90 μM and 0.75 nM, respectively [123].

#### 4.8.6. Inhibition of Tankyrase1/2 Activity

Tankyrases are proteins with poly (ADP-ribose) polymerase activity. Human tankyrases post-translationally modify multiple proteins involved in processes including maintenance of telomere length, sister telomere association, and trafficking of glut4-containing vesicles. The tankyrase protein family consists of tankyrases 1 and 2, which share 85% amino acid sequence identity [169].

Compounds **232** (Figure 19) and **363** (Figure 26), isolated from a marine sponge-associated fungus, *A. flocculosus* 16D-1, showed weak tankyrase1/2 inhibitory activity at a concentration of 40 μM. XAV939 was used as a positive control [113].

#### 4.8.7. Inhibition of Protein Tyrosine Phosphatase 1B (PTP1B) Activity

Protein tyrosine phosphatase 1B (PTP1B) has been shown to be involved in the negative regulation of both insulin and leptin action and was suggested to play an important role in insulin signalling and possibly in obesity in humans [170].

Compound **319** (Figure 25), isolated from a marine sediment-derived fungus, *Altenaria* sp. SF-5016, weakly inhibited the activity of PTP1B, representing 49% inhibition at a concentration of 150 μg/mL [149].

#### 4.8.8. Inhibition of Cyclin-Dependent Kinase 4 (CDK-4) Activity

Cyclin-dependent kinase 4 (CDK-4) is responsible for the phosphorylation of the retinoblastoma gene product (Rb), thus facilitating cell cycle progression. Compound **51** (Figure 8), isolated from a marine red alga-derived fungus strain K063, inhibited CDK-4 activity with IC_50_ = 16.5 μg/mL [56].

### 4.9. Anti-Fouling Activity

Compounds **1**, **2** (Figure 3), **6**–**9** (Figure 4), **29,** and **30** (Figure 5), isolated from the deep-sea-derived fungus, *S. obclavatum* EIODSF 020, were assayed against a settlement of *Bugula neritina* larvae. Compound **7** was found to show strongest activity, inhibiting the *B. neritina* larvae settlement with 77.8%, 69.9%, 68.1%, 56.5%, and 25.4% at concentrations of 25.0, 12.5, 6.25, 3.125, and 1.562 μg/mL, respectively (EC_50_ = 7.8 μg/mL and LC_50_/EC_50_ > 100), indicating that **7** is a potential antifouling candidate. Compounds **1**, **2**, **6**, **8**, **9**, **29**, and **30** displayed, at concentration of 25 μg/mL, 48.7%, 42.3% 40.2%, 12.5%, 23.6%, 19.9%, and 20.1% inhibition against *B. neritina* larvae settlement. Wells containing only filtered sea water with DMSO served as the controls [38].

### 4.10. Lipid-Lowering Activity

Compound **194** (Figure 17), isolated from a marine mud-derived fungus, *A. versicolor* ZLN-60, exhibited a strong lipid-lowering effects in HepG2 cells at a dose of 10 μM in the oil-red O staining assay [101].

Compound **302** (Figure 25), isolated from a mangrove endophytic fungus, *S. kiliense* HDN11-112, significantly reduced oleic acid (OA)-elicited lipid accumulation, as determined from the oil-red O staining, as well as intracellular cholesterol and triglyceride. Compound **302** did not show any cytotoxicity on the tested HepG2 cells at 20 μM, indicating that the lipid-lowering activity was not due to cytotoxicity [136].

### 4.11. Anti-Angiogenesis Activity

Compounds **344**, **345**, and **351** (Figure 26), isolated from a gorgonian coral-associated fungus, *P. chrysogenum* (CHSCLM-0003), were assayed for their pro-angiogenic activity using *Tg(kdrl:EGFP)* transgenic zebrafish line. All the tested compounds induced angiogenesis at concentration of 1.0 μg/mL against zebrafish embryo by promoting the cavity of blood vessels. These compounds were also non-toxic in embryonic zebrafish at a concentration of 100 μg/mL [159].

### 4.12. Anti-Inflammatory Activity

Compound **116** (Figure 14), isolated from a deep-sea sediment-derived fungus, *Aspergillus* sp. SCSIOW2, exhibited an inhibitory activity against a nitric oxide (NO) production, with IC_50_ = 40.3 μg/mL (68.0 μM), in a lipopolysaccharide (LPS) and recombinant mouse interferon-γ (INF-γ)-activated macrophage-like cell line, RAW 264.7. Compound **116** did not show any cytotoxic effect at the tested dose range (30–100 mg/mL). Quercetin was used as a positive control which showed stronger NO inhibitory effect (inhibitory rate 97.4% at 100 mg/mL) [79].

Compound **140** (Figure 14), isolated from a marine sediment-derived fungus, *A. insulicola*, showed anti-inflammatory activity through inhibition of NF-κB luciferase and nitrite production, with IC_50_ values of 8.37 and 13.70 μM, respectively. The corresponding positive control, celastrol, showed an IC_50_ value of 0.3 μM [87].

Compounds **164**–**166** (Figure 15), isolated from a marine sponge-associated fungus, *A. sclerotiorum* ST0501, were assayed for their capacity to inhibit NO production induced by LPS. Compounds **164** and **165** inhibited the NO production better than dexamethasone, the reference drug, with inhibition percentages of 28.92%, 26.31%, and 25.87%, respectively. However, **166** showed a weak inhibition rate at 21.08% [92].

Compounds **109**, **110** (Figure 14), **160**–**162** (Figure 15), and **171**–**176**, **179** (Figure 16), obtained from a deep-sea sediment-derived fungus, *Aspergillus* sp. FS445, were assayed for the inhibition of NO production in LPS-induced mouse macrophage RAW264.7 cells. Compound **175** showed the most significant inhibition of NO production, followed by **173**, **162**, **161**, **176**, and **172**, with IC_50_ values of 16, 20, 25, 37, 57, and 89 μM, respectively. The positive control, aminoguanidine, showed the IC_50_ value of 23 μM [95].

Compounds **188** (Figure 16), **209** (Figure 17), and **216** (Figure 18), isolated from a marine sponge-associated fungus, *A. violaceofuscus*, were investigated for their inhibitory activities against the production of four cytokines (IL-6, IL-10, MCP-1, and TNF-α) in the serum of human acute monocytic leukemia cell line, THP-1, by using the human inflammation cytometric bead array (CBA) assay. Pretreatment of THP-1 cells with **188** and **216** exhibited a significant reduction in the LPS-induced expression of IL-10 with an inhibitory rate of 84.3% and 78.1% (*p* < 0.01), respectively, while **209** showed only a 23.6% decrease. The inhibition of the levels of IL-6, MCP-1, and TNF-α, ranged from 28.0% to 64.2% [99].

Compounds **238**–**244** (Figure 19), isolated from the hydrothermal vent sediment-derived fungus, *A. pseudoviridinutans* TW58-5, were tested for their capacity to inhibit NO production in LPS-induced murine macrophage RAW264.7 cells. Compounds **238**–**244**, particularly **243**, inhibited the NO production at a concentration of 20 μM. Compound **243** also showed the dose-dependent inhibition of NO production. Moreover, **243** effectively reduced the expression of iNOS (the enzyme responsible for NO production) and NLRP3 (an inflammatory mediator), suggesting that **243** could be a promising compound for the development of an anti-inflammatory agent [116].

### 4.13. Inhibition of G-Protein-Coupled Receptors

Compounds **211** and **212** (Figure 18), isolated from a marine sponge-associated fungus, *Stachylidium* sp., were examined for their inhibition of G-protein-coupled receptors (GPCRs) using a radioligand binding assays. Compound **211** showed an affinity to the vasopressin receptor 1A with *Ki* of 7.04 μM, while **212** was found to have a selective affinity to the serotonin receptor 5H_2b_, with *Ki* of 0.77 μM. It is important to note that 5HT_2b_ receptors have been reported to play an important role in cardiac, intestinal, and central levels, and in bone marrow formation and growth [103].

### 4.14. Inhibition of Transporter Proteins

To tackle the resistance of multiple chemotherapeutic agents, targeting the transporter proteins such as P-glycoprotein (P-gp) is vital since it is one of the various factors that contribute to the multidrug resistant (MDR) factors. Thus, P-gp inhibitors that offer the prospect of reversing the MDR phenotype deserve to be investigated. Compound **122** (Figure 14), isolated from a marine sediment-derived fungus, *Aspergillus* sp. (CMB-M081F), was evaluated for P-gp inhibitory activity using a Calcein AM assay. This assay is based on the fact that, in the presence of a P-gp inhibitor, calcein AM efflux is blocked and calcein AM (nonfluorescent) undergoes hydrolysis to calcein (fluorescent), leading to an increase in intracellular fluorescence. Intracellular calcein fluorescence is quantified by cell flow cytometry to arrive at a fluorescence arbitrary ratio (FAR), which measures intracellular calcein fluorescence in cells exposed to a putative P-gp inhibitor, with that from cells not exposed to an inhibitor. Thus, the larger the FAR value, the more effective the P-gp inhibitor. Compound **122** inhibited the P-gp with FAR = 35.5. The positive control, verapamil, exhibited FAR = 72.5 [82].

### 4.15. Anti-Diabetic Activity

In order to examine the insulin sensitivity, the levels of adiponectin production in the adipogenesis model of human bone marrow mesenchymal stem cells (hBM-MSCs) are used. In a cell-based assay, **27** and **28** (Figure 4), isolated from a marine sediment-derived fungus, *A. terreus* FA009, were found to increase the adiponectin production during adipogenesis in hBM-MSCs, with EC_50_ values of 37.1 and 91.9 μM, respectively. The positive controls, glibenclamide and aspirin, showed the EC_50_ values of 3.47 and 145.6 μM, respectively. It is important to note that pharmacological mechanisms for improving the insulin sensitivity of glibenclamide and aspirin are different. The antidiabetic activity of glibenclamide is through binding with sulfonylurea receptor 1 (SUR1), which inhibits the conductance of the adenosine triphosphate (ATP)-dependent potassium (KATP) channel, whereas aspirin inhibits the serine kinase IKKβ. Although aspirin significantly increased the production of adiponectin, the maximum effect achieved by aspirin in the hBM-MSCs was less than that of glibenclamide. Interestingly, **27** induced the maximum increase in adiponectin levels by 56.9% relative to that produced by glibenclamide [48].

### 4.16. Antioxidant Activity

Compound (±)-**189** (Figure 16), isolated from a marine sediment-derived fungus, *Eurotium* sp. SCSIO F452, scavenged the DPPH radicals with an IC_50_ = 58.4 μM, while the pure (−)-**189** showed DPPH radicals scavenging activity with IC_50_ = 159.2 μM. The positive control, ascorbic acid, showed IC_50_ value of 45.8 μM [100].

### 4.17. Wound-Healing Activity

Compound **317** (Figure 25), isolated from a marine sediment-derived fungus, *Acremonium* sp. CNQ-049, did not show cytotoxicity against cancer (Caco2) and non-cancer [HaCaT (a spontaneously immortalized keratinocyte) and NIH3T3 (fibroblast)] cell lines at concentrations below 50 μM. Since **317** enhanced cell viability and increased effectively the mortality of the HaCaT and NIH3T3 cell lines, Kim et al. proposed that **317** might be beneficial for wound healing in the skin of mice by testing three genes related to human wound healing, including actin alpha (ACTC1), collagen type I alpha 2 (COL1A2), and matrix metallopeptidase 1 (MMP1). Quantitative real-time polymerase chain reaction (qRT-PCR) experiments showed that **317** significantly induced the expression of ACTC1 and COL1A2 in both the HaCaT and NIH3T3 cell lines. The increase in COL1A2 expression in both HaCaT and NIH3T3 cells could promote tissue growth and the remodeling phase, thereby potentiating wound closure. On the other hand, ACTC1, an isoform of actin alpha, can play an important role in wound repair by mediating contraction, the recruitment of repair machinery, and cell migration [147].

### 4.18. Larvicidal Activity

Compounds **331** and **332** (Figure 25), isolated from the fermentation extract of *A. flavus*, were tested for larvicidal activity against third-instar larvae of *Culex pipiens* mosquitoes, at concentrations of 10, 50, 100, 200, and 300 ppm. The mortality rates were recorded at various time intervals, i.e., 24, 48, 72, and 96 h. After 96 h of treatment, the highest mortality rates were detected for **331** (ranging from 26.66% to 82.66%), followed by **332** (ranging from 25.33% to 80.0%). Interestingly, **331** exhibited the most potent larvicidal activity, with LC_50 (72h)_ and LC_50 (96h)_ = 69.96 and 58.96 ppm, respectively. The corresponding positive control, toosenedanin, showed LC_50 (72h)_ and LC_90 (72h)_ = 2.66 and 10.0 ppm, respectively. In silico molecular docking studies of **331** and **332** for larvicidal chitinase enzyme activity were performed. Upon docking, the ligand showed the −(C-docker interaction energy) of −49.23 kcal/mol, and six essential hydrogen bonds with Glu177, Tyr245, Asp246, Arg301, and Glu322. On the other hand, **331** and **332** showed relatively better–(C-docker interaction energy) than the ligand, with 65.85 and −59.25 kcal/mol, respectively, The most active conformer of **331** displayed interaction with chitinase through three hydrogen bonds with Arg301, Trp137, and Ser250, while **332** showed further three hydrogen bonds with Asp246, Arg301, and Glu322 [152].

To facilitate the readers, the names and numbers of marine-derived peptides (according to their classes), the producing organisms, the sources from which the fungi were isolated, biological/pharmacological activities of the bioactive peptides, and the corresponding references are listed in Table 1.

## 5. Concluding Remarks and Future Perspectives

With a growing body of research on bioactive peptides, coupled with an accelerating pace of drug development with peptides, nowadays, more than 170 peptides and their derivatives are are in active clinical development [4]. Therefore, efforts to concentrate on marine fungal peptides, which are less considered, deserve further investigations.

A systematic investigation revealed a great potential for marine peptides to modulate critical biological events and pathways. Thus, peptides can serve as valuable models for designing novel lead compounds. Intriguingly, most of the reported biological/pharmacological activities of marine peptides are performed in vitro. Therefore, further investigations in vivo are needed to shed light on the potential of this class of compounds for further structure modifications or semi-synthesis to obtain analogs with better efficacy and stability in vivo. However, when performing in vivo studies, the dynamics shift significantly due to the emergence of peptide bioavailability concerns. For instance, when peptides are administered orally, they face challenges such as gastric acid exposure and enzymatic degradation in the stomach. This necessitates protective strategies to ensure the preservation of peptide integrity. Consequently, ensuring the presence of bioactive peptides at the intended target site demands a reassurance that traditional administration methods may not provide. This is where innovative approaches of pharmaceutical technology like nanoencapsulations, nanoemulsions, and nanotubes for drug delivery come into play. Moreover, the technology of linking bioactive peptides to chimeric antibodies to produce peptide-based drug-conjugates has shown to be an effective way to direct bioactive peptides to the targets. Despite the relevant activities of peptides, safety, tolerability, and efficacy profiles in the human body are fundamental.

Therefore, marine peptides provide salient opportunities not only for expanding the pharmacological profiles but also for designing the synthesis of unique peptides. In recent years, we have witnessed increasing numbers of FDA-approved marine peptides, which reflect the potential of these metabolites for drug discovery and development.

Hence, the importance of marine peptides is being highlighted since more than 36,000 marine natural products possessing various biological activities have been reported so far [171]. Finally, but yet importantly, in order to explore the potential of bioactive peptides, evaluations of less-considered biological/pharmacological targets are also strongly recommended.

**Figure 27:**
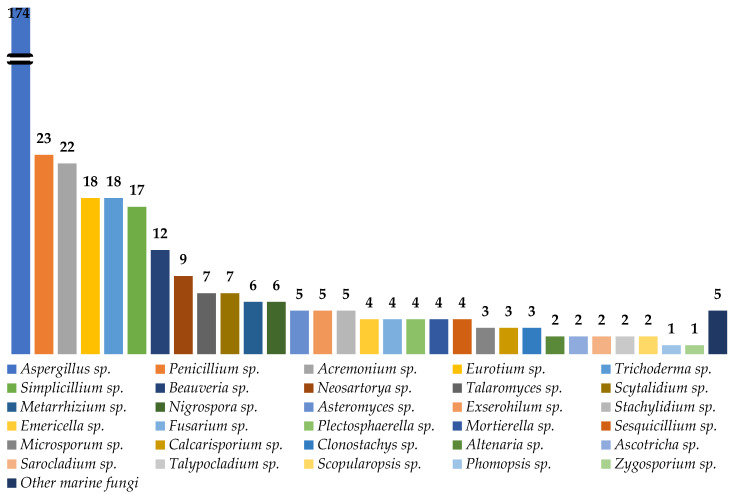
The number of isolated peptides from marine-derived fungal resources.

**Figure 28:**
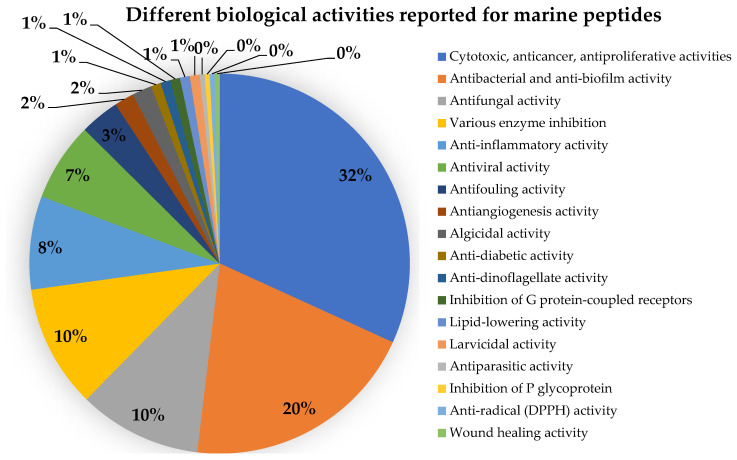
Mode of biological activities of peptides isolated from marine-derived fungal resources and their percentages.

To the best of our knowledge, this review is the first comprehensive review of marine-derived peptides obtained from 30 marine-derived fungal genera, which discusses their chemistry and biological activities. Overall, 366 peptides belonging to various classes are presented (Figure 27). The variation in the amino acid sequences, the chemical features, and a myriad of biological/pharmacological activities, such as cytotoxic activity against various human cancer cell lines, antibacterial and antibiofilm, antifungal, antiviral, antiparasitic, enzyme inhibitory activity, anti-angiogenesis, and anti-inflammatory activity, the inhibition of G-protein-coupled receptors, the inhibition of transporter proteins, anti-diabetic properties, and wound healing make marine peptides an interesting group of compounds to be explored for their potential in drug discovery and other health applications. It is worth mentioning also that a majority of marine peptides were isolated from marine-derived *Aspergillus* species (174 peptides from 48 species), but, to lesser extent, from other fungal genera such as *Acremonium*, *Penicillium*, *Eurotium*, *Talaromyces, Trichoderma,* and others. Moreover, the cytotoxic activity against various human cancer cell lines is the most reported bioactivity for these metabolites. Some of the reported cyclic heptapeptides and cyclic depsipeptides also showed antibacterial and antifungal activities (Figure 28).

Unlike several peptides isolated from marine invertebrates, none of the peptides isolated from marine-derived fungi have made their way to the advanced phase of clinical trial, except for halimide (a naturally occurring diketopiperazine derivative derived from the marine fungus *Aspergillus* sp. CNC139) whose synthetic analog, plinabulin (NPI-2358), was in a complete Phase III clinical trial by BeyondSpring Inc. in development for use in combination with granulocyte colony-stimulating factor for the prevention of chemotherapy-induced neutropenia [172]. Thus, new approaches for optimizing the potential of marine fungal peptides should be considered. The first approach is the optimization and modification of the culture media to stimulate the fungal silence genes to produce novel compounds. In this aspect, although the OSMAC (*O*ne *S*train *MA*ny *C*ompounds) approach has been used to produced new compounds, so far this technique has not yet provided any concrete example of compounds for clinical trials. Another approach that should be considered is the addition of synthetic pharmacophores that contain functional groups that can link peptides to the culture media to explore the possibility of the production of novel bioactive peptides.

## Figures and Tables

**Table 1:** Peptides reported from marine-derived fungi from January 1991 to June 2023.

Compound	Fungal Species/Strain No.	Source of Marine-Derived Fungi	Bioactivity	Ref.
**Linear dipeptides**				
Simplicilliumtide G (**1**)	*Simplicillium obclavatum* EIODSF 020	Marine deep-sea sediment.	Antifouling and cytotoxic activities.	[38]
Simplicilliumtide H (**2**)	*Simplicillium obclavatum* EIODSF 020	Marine deep-sea sediment.	Antifouling and cytotoxic activities.	[38]
Coniosulfide E (**3**)	*Aspergillus unguis* IV17-109	Deep-sea shrimp.	-	[39]
Penicamide A (**4**)	*Penicillium* sp. SCSIO 41512	Marine.	-	[40]
Penicamide B (**5**)	*Penicillium* sp. SCSIO 41512	Marine.	-	[40]
**Linear tripeptides**				
Simplicilliumtide C (**6**)	*Simplicillium obclavatum* EIODSF 020	Marine deep-sea sediment.	Antifouling activity.	[38]
Simplicilliumtide D (**7**)	*Simplicillium obclavatum* EIODSF 020	Marine deep-sea sediment.	Antifouling activity.	[38]
Simplicilliumtide E (**8**)	*Simplicillium obclavatum* EIODSF 020	Marine deep-sea sediment.	Antifouling and cytotoxic activities.	[38]
Simplicilliumtide F (**9**)	*Simplicillium obclavatum* EIODSF 020	Marine deep-sea sediment.	Antifouling activity.	[38]
Aspergillipeptide E (**10**)	*Aspergillus* sp. SCSIO 41501	Marine gorgonian *Melitodes squamata.*	Antiviral activity.	[41]
Aspergillamides C (**11**)	*Aspergillus terreus* SCSIO41008	Marine sponge *Callyspongia* sp.	-	[42]
Aspergillamide D (**12**)	*Aspergillus terreus* SCSIO41008	Marine sponge *Callyspongia* sp.	-	[42]
Aspergillamides A (**13**)	*Aspergillus terreus* SCSIO41008	Marine sponge *Callyspongia* sp.	-	[42]
Aspergillamides B (**14**)	*Aspergillus terreus* SCSIO41008	Marine sponge *Callyspongia* sp.	-	[42]
*cis*-L-phenylalaninamide (**15**)	*Aspergillus terreus* SCSIO41008	Marine sponge *Callyspongia* sp.	-	[42]
*trans*-L-phenylalaninamide (**16**)	*Aspergillus terreus* SCSIO41008	Marine sponge *Callyspongia* sp.	-	[42]
Asterripeptide A (**17**)	*Aspergillus terreus* LM.5.2	Marine mangrove *Kandelia candel.*	Cytotoxic activity.	[43]
Asterripeptide B (**18**)	*Aspergillus terreus* LM.5.2	Marine mangrove *Kandelia candel.*	Cytotoxic and inhibition of SrtA activities.	[43]
Asterripeptide C (**19**)	*Aspergillus terreus* LM.5.2	Marine mangrove *Kandelia candel.*	Cytotoxic and inhibition of SrtA activities.	[43]
Talaropeptin A (**20**)	*Talaromyces purpureogenus* CX11	Marine.	Antifungal activity.	[44]
Talaropeptin B (**21**)	*Talaromyces purpureogenus* CX11	Marine.	Antifungal activity.	[44]
Penilumamide (**22**)	*Penicillium* sp. (strain CNL-338)	Marine red alga *Laurencia* sp.	-	[45]
	*Aspergillus* sp. XS-20090B15	Marine gorgonian *Muricella abnormaliz*.	-	[46]
	*Aspergillus* sp. (33241)	Marine mangrove *Bruguiera sexangula* var. *rhynchopetala.*	-	[47]
Penilumamide B (**23**)	*Aspergillus* sp. XS-20090B15	Marine gorgonian *Muricella abnormaliz*.	-	[46]
Penilumamide C (**24**)	*Aspergillus* sp. XS-20090B15	Marine gorgonian *Muricella abnormaliz.*	-	[46]
Penilumamide D (**25**)	*Aspergillus* sp. XS-20090B15	Marine gorgonian *Muricella abnormaliz.*	-	[46]
Aspergilumamide A (**26**)	*Aspergillus* sp. (33241)	Marine mangrove *Bruguiera sexangula* var. *rhynchopetala.*	-	[47]
Terrelumamide A (**27**)	*Aspergillus terreus* FA009	Marine sediment.	Anti-diabetic and anticancer activities.	[48]
Terrelumamide B (**28**)	*Aspergillus terreus* FA009	Marine sediment.	Anti-diabetic and anticancer activities.	[48]
**Linear tetrapeptides**				
Simplicilliumtide A (**29**)	*Simplicillium obclavatum* EIODSF 020	Marine sediment.	Antifouling and cytotoxic activities.	[38]
Simplicilliumtide B (**30**)	*Simplicillium obclavatum* EIODSF 020	Marine deep-sea sediment.	Antifouling activity.	[38]
Simplicilliumtide I (**31**)	*Simplicillium obclavatum* EIODSF 020	Marine deep-sea sediment.	-	[49]
Aspergillipeptide F (**32**)	*Aspergillus* sp. SCSIO 41501	Marine gorgonian *Melitodes squamata.*	-	[41]
Aspergillipeptide G (**33**)	*Aspergillus* sp. SCSIO 41501	Marine gorgonian *Melitodes squamata.*	-	[41]
Aspergillipeptide H (**34**)	*Aspergillus* sp. SCSIO 41501	Marine gorgonian *Melitodes squamata* Nutting.	-	[50]
Aspergillipeptide I (**35**)	*Aspergillus* sp. SCSIO 41501	Marine gorgonian *Melitodes squamata* Nutting.	-	[50]
Aspergillipeptide J (**36**)	*Aspergillus* sp. SCSIO 41501	Marine gorgonian *Melitodes squamata* Nutting.	-	[50]
Aspergillipeptide K (**37**)	*Aspergillus* sp. SCSIO 41501	Marine gorgonian *Melitodes squamata* Nutting	-	[50]
**Linear hexapeptides**				
FJ120DPB (**38**)	*Aspergillus ochraceopetaliformis.*	Marine sediment.	**Inhibition of enzyme sortase A (SrtA) activity.**	[51]
**Linear octapeptides**				
RHM1 (**39**)	*Acremonium* sp. (UCSC coll. no. 021172 cKZ)	Marine sponge *Teichaxinella* sp. (coll. no. 02172).	Antibacterial and Cytotoxic activities.	[52]
RHM2 (**40**)	*Acremonium* sp. (UCSC coll. no. 021172 cKZ)	Marine sponge *Teichaxinella* sp. (coll. no. 02172).	Cytotoxic activity.	[52]
RHM3 (**41**)	*Acremonium* sp. (UCSC coll. no. 021172 cKZ)	Marine sponge *Teichaxinella* sp. (coll. no. 02172).	-	[53]
RHM4 (**42**)	*Acremonium* sp. (UCSC coll. no. 021172 cKZ)	Marine sponge *Teichaxinella* sp. (coll. no. 02172).	-	[53]
**Linear nonapeptides**				
Aspereline A (**43**)	*Trichoderma asperellum*	Marine sediment.	-	[54]
Aspereline B (**44**)	*Trichoderma asperellum*	Marine sediment.	-	[54]
Aspereline C (**45**)	*Trichoderma asperellum*	Marine sediment.	-	[54]
Aspereline D (**46**)	*Trichoderma asperellum*	Marine sediment.	-	[54]
Aspereline E (**47**)	*Trichoderma asperellum*	Marine sediment.	-	[54]
Aspereline F (**48**)	*Trichoderma asperellum*	Marine sediment.	-	[54]
**Linear undecapeptides**				
Talaropeptide A (**49**)	*Talaromyces* sp. (CMB-TU011)	An unidentified marine tunicate.	Antibacterial activity.	[55]
Talaropeptide C (**50**)	*Talaromyces* sp. (CMB-TU011)	An unidentified marine tunicate.	-	[55]
Dictyonamide A (**51**)	The fungus strain K063.	Marine red alga *Ceratodictyon spongiosum.*	Inhibition of cyclin-dependent kinase 4 activity.	[56]
Dictyonamide B (**52**)	The fungus strain K063.	Marine red alga *Ceratodictyon spongiosum.*	-	[57]
Tolypocaibol A (**53**)	*Tolypocladium* sp.	Marine microalga *Spongomorpha arcta.*	Antibacterial activity.	[57]
Tolypocaibol B (**54**)	*Tolypocladium* sp.	Marine microalga *Spongomorpha arcta.*	Antibacterial activity.	[57]
**Linear dodecapeptides**				
Talaropeptide B (**55**)	*Talaromyces* sp. (CMB-TU011)	An unidentified marine tunicate.	Antibacterial activity.	[55]
Talaropeptide D (**56**)	*Talaromyces* sp. (CMB-TU011)	An unidentified marine tunicate.	-	[55]
**Linear pentadecapeptides**				
Efrapeptin G (**57**)	*Acremonium* sp. (UCSC coll. no. 021172 cKZ)	Marine sponge *Teichaxinella* sp. (coll. no. 02172).	Antibacterial and Cytotoxic activities.	[52,53]
Efrapeptins Eα (**58**)	*Acremonium* sp. (UCSC coll. no. 021172 cKZ)	Marine sponge *Teichaxinella* sp. (coll. no. 02172).	Cytotoxic activity.	[53]
Efrapeptin H (**59**)	*Acremonium* sp. (UCSC coll. no. 021172 cKZ)	Marine sponge *Teichaxinella* sp. (coll. no. 02172).	-	[53]
Efrapeptin E (**60**)	*Acremonium* sp. (UCSC coll. no. 021172 cKZ)	Marine sponge *Teichaxinella* sp. (coll. no. 02172).	-	[53]
Efrapeptin F (**61**)	*Acremonium* sp. (UCSC coll. no. 021172 cKZ)	Marine sponge *Teichaxinella* sp. (coll. no. 02172).	Cytotoxic activity.	[53]
Pentadecaibin I (**62**)	*Trichoderma* sp. MMS1255	Marine sediment.	Antibacterial and cytotoxic activities.	[58]
Pentadecaibin II (**63**)	*Trichoderma* sp. MMS1255	Marine sediment.	Cytotoxic activity.	[58]
Pentadecaibin III (**64**)	*Trichoderma* sp. MMS1255	Marine sediment.	Antibacterial and cytotoxic activities.	[58]
Pentadecaibin IV (**65**)	*Trichoderma* sp. MMS1255	Marine sediment.	Cytotoxic activity.	[58]
Pentadecaibin V (**66**)	*Trichoderma* sp. MMS1255	Marine sediment.	Antibacterial and cytotoxic activities.	[58]
**Linear octadecapeptides**				
Trichorzins A (**67**)	*Trichoderma* sp. GXIMD 01001	Marine sponge *Haliclona* sp.	Antibacterial and cytotoxic activities.	[59]
Trichorzin B (**68**)	*Trichoderma* sp. GXIMD 01001	Marine sponge *Haliclona* sp.	Antibacterial and cytotoxic activities.	[59]
Trichorzin C (**69**)	*Trichoderma* sp. GXIMD 01001	Marine sponge *Haliclona* sp.	Antibacterial and cytotoxic activities.	[59]
Trichorzin D (**70**)	*Trichoderma* sp. GXIMD 01001	Marine sponge *Haliclona* sp.	Antibacterial and cytotoxic activities.	[59]
Trichorzin E (**71**)	*Trichoderma* sp. GXIMD 01001	Marine sponge *Haliclona* sp.	Antibacterial and cytotoxic activities.	[59]
Trichorzin F (**72**)	*Trichoderma* sp. GXIMD 01001	Marine sponge *Haliclona* sp.	Antibacterial and cytotoxic activities.	[59]
Trichorzin G (**73**)	*Trichoderma* sp. GXIMD 01001	Marine sponge *Haliclona* sp.	Antibacterial and cytotoxic activities.	[59]
**Lipophilic linear hexapeptides**				
Halovir A (**74**)	*Scytalidium* sp.	Marine.	Antiviral activity.	[60]
Halovir B (**75**)	*Scytalidium* sp.	Marine.	Antiviral activity.	[60]
Halovir C (**76**)	*Scytalidium* sp.	Marine.	Antiviral activity.	[60]
Halovir D (**77**)	*Scytalidium* sp.	Marine.	Antiviral activity.	[60]
Halovir E (**78**)	*Scytalidium* sp.	Marine.	Antiviral activity.	[60]
**Other linear peptides**				
Flavipeside A (**79**)	*Aspergillus flavipes* 164013	Marine sponge *Dysidea* sp.	Inhibition of pancreatic lipase.	[61]
Flavipeside B (**80**)	*Aspergillus flavipes* 164013	Marine sponge *Dysidea* sp.	Inhibition of pancreatic lipase.	[61]
Flavipeside C (**81**)	*Aspergillus flavipes* 164013	Marine sponge *Dysidea* sp.	Inhibition of pancreatic lipase.	[61]
Asperphenin A (**82**)	*Aspergillus* sp.	Marine-submerged decaying wood.	Antiproliferative activity.	[62]
Asperphenin B (**83**)	*Aspergillus* sp.	Marine-submerged decaying wood.	Antiproliferative activity.	[62]
Fellutamide A (**84**)	*Penicillium fellutanum* Biourge	Marine fish *Apogon endekataenta* Bleeker.	Cytotoxic activity.	[63]
Fellutamide B (**85**)	*Penicillium fellutanum* Biourge	Marine fish *Apogon endekataenta* Bleeker.	Cytotoxic activity.	[63]
**Cyclic dipeptides**				
*cis*-*cyclo*(Leucyl-Tyrosyl) (**86**)	*Penicillium* sp. F37	Marine sponge *Axinella corrugate*	Anti-biofilm formation.	[64]
*cyclo*-(*trans*-4-hydroxy-L-Pro-L-Leu) (**87**)	*Aspergillus niger* BRF-074	Marine sediment.	-	[65]
*cyclo*-(*trans*-4-hydroxy-L-Pro-L-Phe) (**88**)	*Aspergillus niger* BRF-074	Marine sediment.	-	[65]
*cyclo*-(L-Pro-L-Leu) (**89**)	*Aspergillus niger* BRF-074	Marine sediment.	-	[65]
	*Fusarium* sp. RWS56-10	Marine hydrothermal vent sediment.	-	[66]
*cyclo*-(L-Pro-L-Val) (**90**)	*Aspergillus niger* BRF-074	Marine sediment.	-	[65]
*cyclo*-(Phe-Pro) (**91**)	*Penicillium* sp. WF-06	Marine sediment.	-	[86]
	*Aspergillus terreus* SCSIO41008	Marine sponge *Callyspongia* sp.	-	[42]
	*Aspergillus niger* BRF-074	Marine sediment.	-	[65]
*cyclo*-(L-Pro-L-Tyr) (**92**)	*Aspergillus ochraceopetaliformis* DSW-2	Sea water.	Weak cytotoxic activity.	[78]
	*Aspergillus niger* BRF-074	Marine sediment.	-	[65]
	*Penicillium chrysogenum* DXY-1	Marine sediment.	Antibacterial activity and anti-biofilm formation	[67]
Penicimutide (**93**)	*Penicillium purpurogenum* G59	Marine sediment.	Cytotoxic activity.	[68]
*cyclo*-(L-Ile-L-Pro) (**94**)	*Penicillium purpurogenum* G59	Marine sediment.	-	[68]
*cyclo*-(Pro-Ala) (**95**)	*Ascotricha* sp. ZJ-M-5	Marine.	-	[69]
*cyclo*-(Ile-Leu) (**96**)	*Ascotricha* sp. ZJ-M-5	Marine.	-	[69]
*cyclo*-(Gly-Pro) (**97**)	*Penicillium* sp. WF-06	Marine sediment.	-	[86]
Fellutanine A analogue (**98**)	*Neosartorya glabra* KUFA 0702	Marine sponge *Mycale* sp.	-	[70]
Aszonalenin (**99**)	*Neosartorya glabra* KUFA 0702	Marine sponge *Mycale* sp.	-	[70]
	*Neosartorya takakii* KUFC 7898	Marine alga *Amphiroa* sp.	-	[72]
(3*R*)-3-(1*H*-Indol-3-ylmethyl)-3,4-dihydro-1*H*-1,4-benzodiazepine-2,5-dione (**100**)	*Neosartorya glabra* KUFA 0702	Marine sponge *Mycale* sp.	-	[70]
Takakiamide (**101**)	*Neosartorya glabra* KUFA 0702	Marine sponge *Mycale* sp.	-	[70]
	*Neosartorya takakii* KUFC 7898	Marine alga *Amphiroa* sp.	-	[72]
(11a*R*)-2,3-Dihydro-1*H*-pyrrolo{2,1-*c*}{1,4}benzodiazepine-5,11(10*H*,11a*H*)-dione (**102**)	*Neosartorya glabra* KUFA 0702	Marine sponge *Mycale* sp.	-	[70]
Fellutanine A (**103**)	*Neosartorya glabra* KUFA 0702	Marine sponge *Mycale* sp.	-	[70]
Acyl aszonalenin (**104**)	*Aspergillus carneus* (MST-MF156)	Estuarine sediment.	-	[71]
	*Neosartorya takakii* KUFC 7898	Marine alga *Amphiroa* sp.	-	[72]
Terretrione B (**105**)	*Aspergillus terreus* SCSIO41008	Marine sponge *Callyspongia* sp.	-	[42]
Terretrione C (**106**)	*Aspergillus terreus* SCSIO41008	Marine sponge *Callyspongia* sp.	-	[42]
Brevianamide F (**107**)	*Aspergillus terreus* SCSIO41008	Marine sponge *Callyspongia* sp.	-	[42]
*cyclo*-(L-Trp-L-Ala) (**108**)	*Eurotium chevalieri* MUT2316	Marine sponge *Grantia compressa.*	Antibacterial and algicidal activities.	[73]
	*Penicillium* sp. WF-06	Marine sediment.	-	[86]
Neoechinulin A (**109**)	*Eurotium chevalieri* MUT2316	Marine sponge *Grantia compressa.*	Algicidal activity.	[73]
	*Microsporum* sp.	Marine red algae.	Cytotoxic activity and induction of apoptosis.	[74]
Echinulin (**110**)	*Eurotium chevalieri* MUT2316	Marine sponge *Grantia compressa.*	Antibacterial and algicidal activities.	[73]
	*Eurotium repens*	Marine sponge *Suberites domuncula*	-	[75]
	*Eurotium chevalieri* KUFA 0006	Mangrove plant *Rhizophora mucronata* Poir.	-	[88]
*cyclo*-(L-Trp-L-Ile) (**111**)	*Aspergillus niger* EN-13	Marine brown alga *Colpomenia sinuosa.*	-	[76]
*cyclo*-(L-Trp-L-Phe) (**112**)	*Aspergillus niger* EN-13	Marine brown alga *Colpomenia sinuosa.*	-	[76]
*cyclo*-(L-Trp-L-Tyr) (**113**)	*Aspergillus niger* EN-13	Marine brown alga *Colpomenia sinuosa.*	-	[76]
(3*S*,6*S*)-3,6-dibenzylpiperazine-2,5-dione (**114**)	*Aspergillus candidus* KUFA0062	Marine sponge *Epipolasis* sp.	-	[77]
Mactanamide (**115**)	*Aspergillus ochraceopetaliformis* DSW-2	Sea water.	Weak cytotoxic activity.	[78]
14-Hydroxy-cyclopeptine (**116**)	*Aspergillus* sp. SCSIOW2	Marine deep-sea sediment.	Inhibition of nitric oxide production.	[79]
Protuboxepin A (**117**)	*Aspergillus* sp. SF-5044	Marine intertidal sediment.	Cytotoxic activity.	[80]
Protuboxepin B (**118**)	*Aspergillus* sp. SF-5044	Marine intertidal sediment.	-	[80]
Protubonine A (**119**)	*Aspergillus* sp. SF-5044	Marine intertidal sediment.	-	[80]
Protubonine B (**120**)	*Aspergillus* sp. SF-5044	Marine intertidal sediment.	-	[80]
Oxepinamide E (**121**)	*Aspergillus* sp. (BM-05 and BM-05ML)	Marine brown alga *Sargassum* sp.		[81]
Shornephine A (**122**)	*Aspergillus* sp. (CMB-M081F)	Marine sediment.	Inhibition of P-glycoprotein	[82]
15b-β-Hydroxy-5-*N*-acetyladreemin (**123**)	*Aspergillus* sp. (CMB-M081F)	Marine sediment.	-	[82]
5-*N*-Acetyladreemin (**124**)	*Aspergillus* sp. (CMB-M081F)	Marine sediment.	-	[82]
15b-β-Methoxy-5-*N*-acetyladreemin (**125**)	*Aspergillus* sp. (CMB-M081F)	Marine sediment.	-	[82]
Hyalodendrin (**126**)	*Asteromyces cruciatus* 763	An unidentified decaying green alga.	-	[83]
Gliovictin (**127**)	*Asteromyces cruciatus* 763	An unidentified decaying green alga.	-	[83]
^1^*N*-norgliovictin (**128**)	*Asteromyces cruciatus* 763	An unidentified decaying green alga.	-	[83]
Bis-*N*-norgliovictin (**129**)	*Asteromyces cruciatus* 763	An unidentified decaying green alga.	Antibacterial and antifungal activities.	[83]
(±)-7,8-epoxy-brevianamide Q ((±)-**130**)	*Aspergillus versicolor* MF180151	Marine sediment.	-	[84]
(±)-8-hydroxy-brevianamide R ((±)-**131**)	*Aspergillus versicolor* MF180151	Marine sediment.	-	[84]
(±)-8-epihydroxy-brevianamide R ((±)-**132**)	*Aspergillus versicolor* MF180151	Marine sediment.	-	[84]
(±)-Brevianamide R ((±)-**133**)	*Aspergillus versicolor* MF180151	Marine sediment.	-	[84]
	*Aspergillus versicolor* HBU-7	Sea mud sample.	-	[90]
Rostratin A (**134**)	*Exserohilum rostratum* (Drechsler) CNK-630	Marine cyanobacterial mat.	Cytotoxic activity.	[85]
Rostratin B (**135**)	*Exserohilum rostratum* (Drechsler) CNK-630	Marine cyanobacterial mat.	Cytotoxic activity.	[85]
Rostratin C (**136**)	*Exserohilum rostratum* (Drechsler) CNK-630	Marine cyanobacterial mat.	Cytotoxic activity.	[85]
Rostratin D (**137**)	*Exserohilum rostratum* (Drechsler) CNK-630	Marine cyanobacterial mat.	Cytotoxic activity.	[85]
Exserohilone (**138**)	*Exserohilum rostratum* (Drechsler) CNK-630	Marine cyanobacterial mat.	-	[85]
Gliocladine C (**139**)	*Penicillium* sp. WF-06	Marine Sediment.	Cytotoxic activity.	[86]
Azonazine (**140**)	*Aspergillus insulicola*	Marine sediment.	Anti-angiogenesis activity.	[87]
(11*R*,14*S*)-3-(1*H*-Indol-3ylmethyl)-6-isopropyl-2,5-piperazinedione (**141**)	*Eurotium chevalieri* KUFA 0006	Mangrove plant *Rhizophora mucronata* Poir.	-	[88]
Preechinulin (**142**)	*Eurotium chevalieri* KUFA 0006	Mangrove plant *Rhizophora mucronata* Poir.	-	[88]
Neoechinulin E (**143**)	*Eurotium chevalieri* KUFA 0006	Mangrove plant *Rhizophora mucronata* Poir.	-	[88]
Eurocristatine (**144**)	*Eurotium chevalieri* KUFA 0006	Mangrove plant *Rhizophora mucronata* Poir.	-	[88]
Brevianamide S (**145**)	*Aspergillus versicolor* (MF030)	Marine sediment.	Antibacterial activity.	[89]
Brevianamide T (**146**)	*Aspergillus versicolor* (MF030)	Marine sediment.	-	[89]
Brevianamide U (**147**)	*Aspergillus versicolor* (MF030)	Marine sediment.	-	[89]
Brevianamide V (**148**)	*Aspergillus versicolor* (MF030)	Marine sediment.	-	[89]
	*Aspergillus versicolor* HBU-7	Sea mud sample.	Cytotoxic activity.	[90]
Brevianamide N (**149**)	*Aspergillus versicolor* (MF030)	Marine sediment.	-	[89]
Brevianamide K (**150**)	*Aspergillus versicolor* (MF030)	Marine sediment.	-	[89]
	*Aspergillus versicolor* HBU-7	Sea mud sample.	-	[90]
Deoxy brevianamide E (**151**)	*Aspergillus versicolor* (MF030)	Marine sediment.	-	[89]
	*Aspergillus versicolor* HBU-7	Sea mud sample.	-	[90]
(±)-Brevianamide Z ((±)-**152**)	*Aspergillus versicolor* HBU-7	Sea mud sample.	-	[90]
(±)-Brevianamide Z1 ((±)-**153**)	*Aspergillus versicolor* HBU-7	Sea mud sample.	-	[90]
(±)-Brevianamide X ((±)-**154**)	*Aspergillus versicolor* HBU-7	Sea mud sample.	-	[90]
(±)-Brevianamide Q ((±)-**155**)	*Aspergillus versicolor* HBU-7	Sea mud sample.	-	[90]
12-Demethyl-12-oxo-eurotechinulin B (**156**)	*Eurotium rubrum* G2	Marine semi-mangrove plant *Hibiscus tiliaceus* LINN.	Cytotoxic activity.	[91]
Variecolorin J (**157**)	*Eurotium rubrum* G2	Marine semi-mangrove plant *Hibiscus tiliaceus* LINN	-	[91]
Eurotechinulin B (**158**)	*Eurotium rubrum* G2	Marine semi-mangrove plant *Hibiscus tiliaceus* LINN	-	[91]
Variecolorin G (**159**)	*Eurotium rubrum* G2	Marine semi-mangrove plant *Hibiscus tiliaceus* LINN	Cytotoxic activity.	[91]
Alkaloid E-7 (**160**)	*Eurotium rubrum* G2	Marine semi-mangrove plant *Hibiscus tiliaceus* LINN	Cytotoxic activity.	[91]
	*Aspergillus* sp. FS445	Marine deep sediment.	Anti-inflammatory activity.	[95]
Cryptoechinuline G (**161**)	*Eurotium rubrum* G2	Marine semi-mangrove plant *Hibiscus tiliaceus* LINN	-	[91]
	*Aspergillus* sp. FS445	Marine deep sediment.	Anti-inflammatory activity.	[95]
Isoechinulin B (**162**)	*Eurotium rubrum* G2	Marine semi-mangrove plant *Hibiscus tiliaceus* LINN	-	[91]
	*Aspergillus* sp. FS445	Marine deep sediment.	Anti-inflammatory activity.	[95]
7-Isopentenylcryptoechinuline D (**163**)	*Eurotium rubrum* G2	Marine semi-mangrove plant *Hibiscus tiliaceus* LINN	-	[91]
Sclerotioloid A (**164**)	*Aspergillus sclerotiorum* ST0501	Inner tissue of marine sponge.	Anti-inflammatory activity.	[92]
Sclerotioloid B (**165**)	*Aspergillus sclerotiorum* ST0501	Inner tissue of marine sponge.	Anti-inflammatory activity.	[92]
Sclerotioloid C (**166**)	*Aspergillus sclerotiorum* ST0501	Inner tissue of marine sponge.	Weak anti-inflammatory activity.	[92]
Gartryprostatin C (**167**)	*Aspergillus sclerotiorum* ST0501	Inner tissue of marine sponge.	-	[92]
Speramide C (**168**)	*Aspergillus sclerotiorum* ST0501	Inner tissue of marine sponge.	-	[92]
Asperopiperazine A (**169**)	*Aspergillus* sp. DY001	Red Sea tunicate *Didemnum* sp.	Antibacterial, antifungal, and cytotoxic activities.	[93]
Asperopiperazine B (**170**)	*Aspergillus* sp. DY001	Red Sea tunicate *Didemnum* sp.	Antibacterial, antifungal, and cytotoxic activities.	[93]
Asperchinulin A (**171**)	*Aspergillus* sp. FS445	Marine deep sediment.	-	[95]
Asperchinulin B (**172**)	*Aspergillus* sp. FS445	Marine deep sediment.	Anti-inflammatory activity.	[95]
Asperchinulin C (**173**)	*Aspergillus* sp. FS445	Marine deep sediment.	Anti-inflammatory activity.	[95]
Asperchinulin D (**174**)	*Aspergillus* sp. FS445	Marine deep sediment.	-	[95]
Neoechinulin B (**175**)	*Aspergillus* sp. FS445	Marine deep sediment.	Anti-inflammatory activity.	[95]
			Antibacterial activity.	[96]
Isoechinulin A (**176**)	*Aspergillus* sp. FS445	Marine deep sediment.	-	[95]
Tardioxopiperazine A (**177**)	*Aspergillus* sp. FS445	Marine deep sediment.	-	[95]
24,25-dihydroxyvariecolorin G (**178**)	*Aspergillus chevalieri* CS-122	Marine deep-sea cold seep.	Antibacterial activity.	[96]
25-hydroxyrubrumazine B (**179**)	*Aspergillus chevalieri* CS-122	Marine deep-sea cold seep.	Antibacterial activity.	[96]
22-chloro-25-hydroxyrubrumazine B (**180**)	*Aspergillus chevalieri* CS-122	Marine deep-sea cold seep.	Antibacterial activity.	[96]
25-hydroxyvariecolorin F (**181**)	*Aspergillus chevalieri* CS-122	Marine deep-sea cold seep.	Antibacterial activity.	[96]
27-*epi*-aspechinulin D (**182**)	*Aspergillus chevalieri* CS-122	Marine deep-sea cold seep.	Antibacterial activity.	[96]
Asperazine (**183**)	*Aspergillus niger*	Caribbean sponge *Hyrtiosproteus* sp.	-	[97]
Plectosphaeroic acid A (**184**)	*Plectosphaerella cucumerina*	Marine sediment.	Inhibition of indoleamine 2,3-dioxygenase.	[98]
Plectosphaeroic acid B (**185**)	*Plectosphaerella cucumerina*	Marine sediment.	Inhibition of indoleamine 2,3-dioxygenase.	[98]
Plectosphaeroic acid C (**186**)	*Plectosphaerella cucumerina*	Marine sediment.	Inhibition of indoleamine 2,3-dioxygenase.	[98]
T988 A (**187**)	*Plectosphaerella cucumerina*	Marine sediment.	-	[98]
Diketopiperazine dimer (**188**)	*Aspergillus violaceofuscus*	Marine Sponge *Reniochalina* sp.	Anti-inflammatory activity.	[99]
Variecolortin A (**189**)	*Eurotium* sp. SCSIO F452	Marine sediment.	DPPH^•^ radical scavenging activity.	[100]
Variecolortin B (**190**)	*Eurotium* sp. SCSIO F452	Marine sediment.	Cytotoxic activity.	[100]
Variecolortin C (**191**)	*Eurotium* sp. SCSIO F452	Marine sediment.	Cytotoxic activity.	[100]
**Cyclic tripeptides**				
Psychrophilin E (**192**)	*Aspergillus* sp. (BN-05 & BM-05ML)	Marine brown alga *Sargassum* sp.	Cytotoxic activity.	[81]
	*Aspergillus versicolor* ZLN-60	Marine mud.	-	[101]
Psychrophilin F (**193**)	*Aspergillus versicolor* ZLN-60	Marine mud.	-	[101]
Psychrophilin G (**194**)	*Aspergillus versicolor* ZLN-60	Marine mud.	Lipid-lowering activity.	[101]
Psychrophilin H (**195**)	*Aspergillus versicolor* ZLN-60	Marine mud.	-	[101]
Sclerotiotide A (**196**)	*Aspergillus sclerotiorum* PT06-1	Marine salt sediment.	Antifungal activity.	[102]
Sclerotiotide B (**197**)	*Aspergillus sclerotiorum* PT06-1	Marine salt sediment.	Antifungal activity.	[102]
	*Aspergillus ochraceopetaliformis* DSW-2	Sea water.	Weak cytotoxic activity.	[78]
Sclerotiotide C (**198**)	*Aspergillus sclerotiorum* PT06-1	Marine salt sediment.	-	[102]
Sclerotiotide D (**199**)	*Aspergillus sclerotiorum* PT06-1	Marine salt sediment.	-	[102]
Sclerotiotide E (**200**)	*Aspergillus sclerotiorum* PT06-1	Marine salt sediment.	-	[102]
Sclerotiotide F (**201**)	*Aspergillus sclerotiorum* PT06-1	Marine salt sediment.	Antifungal activity.	[102]
	*Aspergillus ochraceopetaliformis* DSW-2	Sea water.	Weak cytotoxic activity.	[78]
Sclerotiotide G (**202**)	*Aspergillus sclerotiorum* PT06-1	Marine salt sediment.	-	[102]
Sclerotiotide H (**203**)	*Aspergillus sclerotiorum* PT06-1	Marine salt sediment.	-	[102]
Sclerotiotide I (**204**)	*Aspergillus sclerotiorum* PT06-1	Marine salt sediment.	Antifungal activity.	[102]
Sclerotiotide J (**205**)	*Aspergillus sclerotiorum* PT06-1	Marine salt sediment.	-	[102]
Sclerotiotide K (**206**)	*Aspergillus sclerotiorum* PT06-1	Marine salt sediment.	-	[102]
JBIR-15 (**207**)	*Aspergillus sclerotiorum* PT06-1	Marine salt sediment.	Antifungal activity.	[102]
Aspochracin (**208**)	*Aspergillus sclerotiorum* PT06-1	Marine salt sediment.	-	[102]
Sclerotiotide L (**209**)	*Aspergillus violaceofuscus*	Marine sponge *Reniochalina* sp.	Weak anti-inflammatory activity.	[99]
Sclerotiotide M (**210**)	*Aspergillus ochraceopetaliformis* DSW-2	Sea water.	Weak cytotoxic activity.	[78]
**Cyclic tetrapeptides**				
Endolide A (**211**)	*Stachylidium* sp.	Marine sponge *Callyspongia* sp. sf. C. *flammea.*	Inhibition of G-protein-coupled receptors.	[103]
Endolide B (**212**)	*Stachylidium* sp.	Marine sponge *Callyspongia* sp. sf. C. *flammea.*	Inhibition of G-protein-coupled receptors.	[103]
Endolide C (**213**)	*Stachylidium* sp. 293 K04	Marine sponge *Callyspongia* sp. sf. C. *flammea.*	-	[104]
Endolide D (**214**)	*Stachylidium* sp. 293 K04	Marine sponge *Callyspongia* sp. sf. C. *flammea.*	-	[104]
Hirsutide (**215**)	*Stachylidium* sp. 293 K04	Marine sponge *Callyspongia* sp. sf. C. *flammea.*	-	[104]
Violaceomide A (**216**)	*Aspergillus violaceofuscus*	Marine Sponge *Reniochalina* sp.	Anti-inflammatory activity.	[99]
Sartoryblabramide A (**217**)	*Neosartorya glabra* KUFA 0702	Marine sponge *Mycale* sp.	-	[70]
Sartoryblabramide B (**218**)	*Neosartorya glabra* KUFA 0702	Marine sponge *Mycale* sp.	-	[70]
*cyclo*-(L-leucyl-*trans*-4-hydroxy-L-prolyl-D-leucyl-*trans*-4-hydroxy-L-proline) (**219**)	Co-culture of *Phomopsis* sp. K38 & *Altenaria* sp. E33	Mangrove.	Antifungal activity.	[105]
5,5ʹ-epoxy-MKN-349A (**220**)	*Penicilluim* sp. GD6	Marine mangrove *Bruguiera gymnorrhiza.*	-	[107]
Asperterrestide A (**221**)	*Aspergillus terreus* SCSGAF0162	Marine gorgonian *Echinogorgia aurantiaca.*	Antiviral and cytotoxic activities.	[108]
Compound (**222**)	*Aspergillus flavipes*	Marine isopod *Ligia oceanica.*	-	[109]
Microsporin A (**223**)	*Microsporum* cf. *gypseum*	Marine bryozoan *Bugula* sp.	Cytotoxic and histone deacethylase inhibitory activities.	[110]
Microsporin B (**224**)	*Microsporum* cf. *gypseum*	Marine bryozoan *Bugula* sp.	Cytotoxic activity.	[110]
**Cyclic penapeptides**				
Caletasin (**225**)	*Aspergillus* sp. MEXU27854	Marine intertidal sand.	-	[111]
Asperpeptide A (**226**)	*Aspergillus* sp. XS-20090B15	Marine gorgonian *Muricella abnormaliz.*	Antibacterial activity.	[46]
Cotteslosin A (**227**)	*Aspergillus versicolor* (MST-MF495)	Low-tide region.	Cytotoxic activity.	[112]
Cotteslosin B (**228**)	*Aspergillus versicolor* (MST-MF495)	Low-tide region.	-	[112]
Lajollamide A (**229**)	*Asteromyces cruciatus* 763	An unidentified decaying green alga.	Antibacterial activity.	[83]
JG002CPA (**230**)	*Aspergillus allahabadii*	Marine sediment.	**Inhibition of enzyme sortase A (SrtA) activity.**	[51]
JG002CPA (**231**)	*Aspergillus allahabadii*	Marine sediment.	**Inhibition of sortase A (SrtA) and isocitrate lyase (ICL) activities.**	[51]
Asperflomide (**232**)	*Aspergillus flocculosus* 16D-1	Marine sponge *Phakellia fusca.*	Inhibition of tankyrase1/2 activity.	[113]
Malformin C (**233**)	*Aspergillus* sp. SCSIOW2	Marine sediment.	Algicidal activity.	[114]
	*Aspergillus niger* BRF-074	Marine sediment.	-	[65]
	*Aspergillus niger*	Marine sponge *Hyrtiosproteus* sp.	-	[97]
Malformin A1 (**234**)	*Aspergillus niger* BRF-074	Marine sediment.	-	[65]
Aspergillipeptide D (**235**)	*Aspergillus* sp. SCSIO 41501	Marine gorgonian *Melitodes squamata.*	Antiviral activity.	[41]
Versicotide A (**236**)	*Aspergillus versicolor* ZLN-60	Marine mud.	-	[115]
Versicotide B (**237**)	*Aspergillus versicolor* ZLN-60	Marine mud.	-	[115]
Pseudoviridinutan A (**238**)	*Aspergillus pseudoviridinutans* TW58-5	Marine hydrothermal vent sediment.	Anti-inflammatory activity.	[116]
Pseudoviridinutan B (**239**)	*Aspergillus pseudoviridinutans* TW58-5	Marine hydrothermal vent sediment.	Anti-inflammatory activity.	[116]
Pseudoviridinutan C (**240**)	*Aspergillus pseudoviridinutans* TW58-5	Marine hydrothermal vent sediment.	Anti-inflammatory activity.	[116]
Pseudoviridinutan D (**241**)	*Aspergillus pseudoviridinutans* TW58-5	Marine hydrothermal vent sediment.	Anti-inflammatory activity.	[116]
Pseudoviridinutan E (**242**)	*Aspergillus pseudoviridinutans* TW58-5	Marine hydrothermal vent sediment.	Anti-inflammatory activity.	[116]
Pseudoviridinutan F (**243**)	*Aspergillus pseudoviridinutans* TW58-5	Marine hydrothermal vent sediment.	Anti-inflammatory activity.	[116]
Pseudoviridinutan G (**244**)	*Aspergillus pseudoviridinutans* TW58-5	Marine hydrothermal vent sediment.	Anti-inflammatory activity.	[116]
**Cyclic hexapeptides**				
Aspersymmetide A (**245**)	*Aspergillus versicolor* (TA01-14)	Marine gorgonian coral *Carijoa* sp. (GX-WZ-2010001).	Cytotoxic activity.	[117]
Simplicilliumtide J (**246**)	*Simplicillium obclavatum* EIODSF 020	Marine deep-sea sediment.	Antifungal and antiviral activities.	[49]
	*Simplicillium obclavatum* EIODSF 020	Marine deep-sea sediment.	Antifungal activity.	[118]
Simplicilliumtide K (**247**)	*Simplicillium obclavatum* EIODSF 020	Marine deep-sea sediment.	-	[49]
Simplicilliumtide L (**248**)	*Simplicillium obclavatum* EIODSF 020	Marine deep-sea sediment.	-	[49]
Simplicilliumtide M (**249**)	*Simplicillium obclavatum* EIODSF 020	Marine deep-sea sediment.	-	[49]
Verlamelin A (**250**)	*Simplicillium obclavatum* EIODSF 020	Marine deep-sea sediment.	Antifungal and antiviral activities.	[49]
	*Simplicillium obclavatum* EIODSF 020	Marine deep-sea sediment.	Antifungal activity.	[118]
Verlamelin B (**251**)	*Simplicillium obclavatum* EIODSF 020	Marine deep-sea sediment.	Antifungal and antiviral activities.	[49]
	*Simplicillium obclavatum* EIODSF 020	Marine deep-sea sediment.	Antifungal activity.	[118]
Simplicilliumtide N (**252**)	*Simplicillium obclavatum* EIODSF 020	Marine deep-sea sediment.	Antifungal activity.	[118]
Simplicilliumtide O (**253**)	*Simplicillium obclavatum* EIODSF 020	Marine deep-sea sediment.	Antifungal activity.	[118]
Versicotide C (**254**)	*Aspergillus versicolor* ZLN-60	Marine mud.	-	[101]
Sclerotide A (**255**)	*Aspergillus sclerotiorum* PT06-1	Putain sea salt filed.	Antifungal activity.	[119]
	*Aspergillus sclerotiorum* SCSIO41031	Marine soft coral.	-	[120]
Sclerotide B (**256**)	*Aspergillus sclerotiorum* PT06-1	Putain sea salt filed.	Antifungal, antibacterial, and cytotoxic activities.	[119]
Sclerotide C (**257**)	*Aspergillus sclerotiorum* SCSIO41031	Marine soft coral.	-	[120]
Sclerotide D (**258**)	*Aspergillus sclerotiorum* SCSIO41031	Marine soft coral.	-	[120]
Sclerotide E (**259**)	*Aspergillus sclerotiorum* SCSIO41031	Marine soft coral.	-	[120]
Similanamide (**260**)	*Aspergillus similanensis* KUFA0013	Marine sponge *Rhabdermia* sp.	Cytotoxic activity.	[121]
Acremonpeptide A (**261**)	*Acremonium persicinum* SCSIO115.	Marine sediment.	Antiviral activity.	[122]
Acremonpeptide B (**262**)	*Acremonium persicinum* SCSIO115.	Marine sediment.	Antiviral activity.	[122]
Acremonpeptide C (**263**)	*Acremonium persicinum* SCSIO115.	Marine sediment.	Antiviral activity.	[122]
Acremonpeptide D **264**)	*Acremonium persicinum* SCSIO115.	Marine sediment.	Antiviral activity.	[122]
Al(III)-acremonpeptide D (**265**)	*Acremonium persicinum* SCSIO115.	Marine sediment.	Antiviral activity.	[122]
Petrosamide A (**266**)	*Aspergillus* sp. 151304	Marine sponge *Petrosia* sp.	**Inhibition of pancreatic lipase activity.**	[123]
Petrosamide A (**267**)	*Aspergillus* sp. 151304	Marine sponge *Petrosia* sp.	**Inhibition of pancreatic lipase activity.**	[123]
Petrosamide A (**268**)	*Aspergillus* sp. 151304	Marine sponge *Petrosia* sp.	**Inhibition of pancreatic lipase activity.**	[123]
**Cyclic heptapeptides**				
Asperversiamide A (**269**)	*Aspergillus versicolor* (CHNSCLM-0063)	Marine coral.	Antibacterial activity.	[124]
Asperversiamide B (**270**)	*Aspergillus versicolor* (CHNSCLM-0063)	Marine coral.	Antibacterial and anti-*Mycobacterium tuberculosis* activities.	[124]
Asperversiamide C (**271**)	*Aspergillus versicolor* (CHNSCLM-0063)	Marine coral.	Antibacterial activity.	[124]
Asperheptatide A (**272**)	*Aspergillus versicolor* (CHNSCLM-0063)	Marine gorgonian coral *Rumphella aggregata.*	Anti-*Mycobacterium tuberculosis* activity.	[125]
Asperheptatide B (**273**)	*Aspergillus versicolor* (CHNSCLM-0063)	Marine gorgonian coral *Rumphella aggregata.*	Anti-*Mycobacterium tuberculosis* activity.	[125]
Asperheptatide C (**274**)	*Aspergillus versicolor* (CHNSCLM-0063)	Marine gorgonian coral *Rumphella aggregata.*	-	[125]
Asperheptatide D (**275**)	*Aspergillus versicolor* (CHNSCLM-0063)	Marine gorgonian coral *Rumphella aggregata.*	-	[125]
Cordyheptapeptide C (**276**)	*Acremonium persicinum* SCSIO115	Marine sediment.	Cytotoxic activity.	[126]
Cordyheptapeptide D (**277**)	*Acremonium persicinum* SCSIO115	Marine sediment.	Cytotoxic activity.	[126]
Cordyheptapeptide E (**278**)	*Acremonium persicinum* SCSIO115	Marine sediment.	Cytotoxic activity.	[126]
Talarolide A (**279**)	*Talaromyces* sp. (CMB-TU011)	Marine tunicate.	-	[127]
Mortiamide A (**280**)	*Mortierella* sp. RKAG 110	Marine sediment.	-	[128]
Mortiamide B (**281**)	*Mortierella* sp. RKAG 110	Marine sediment.	-	[128]
Mortiamide C (**282**)	*Mortierella* sp. RKAG 110	Marine sediment.	-	[128]
Mortiamide D (**283**)	*Mortierella* sp. RKAG 110	Marine sediment.	-	[128]
Unguisin A (**284**)	*Emericella unguis*	*Stomolopus meliagris* (medusa)	Antibacterial activity.	[129]
	*Aspergillus unguis* 6-20-6 GDMCC 60337	Seaweed.	Antibacterial activity.	[130]
	*Aspergillus unguis* IV17-109	Deep-sea shrimp.	-	[39]
Unguisin B (**285**)	*Emericella unguis*	*Stomolopus meliagris* (medusa).	Antibacterial activity.	[129]
Unguisin E (**286**)	*Aspergillus* sp. AF119	Soil of Xiamen beach.	-	[131]
Scytalidamide A (**287**)	*Scytalidium* sp. CNC-310	Marine green alga *Halimedia* sp.	Cytotoxic activity.	[132]
Scytalidamide B (**288**)	*Scytalidium* sp. CNC-310	Marine green alga *Halimedia* sp.	Cytotoxic activity.	[132]
Maribasin C (**289**)	*Aspergillus* sp. SCSIO 41501	Marine gorgonian *Melitodes squamata* Nutting.	Antifungal activity.	[50]
Maribasin D (**290**)	*Aspergillus* sp. SCSIO 41501	Marine gorgonian *Melitodes squamata* Nutting.	Antifungal activity.	[50]
Maribasin E (**291**)	*Aspergillus* sp. SCSIO 41501	Marine gorgonian *Melitodes squamata* Nutting.	Antifungal activity.	[50]
Maribasin A (**292**)	*Aspergillus* sp. SCSIO 41501	Marine gorgonian *Melitodes squamata* Nutting.	Antifungal activity.	[50]
Maribasin B (**293**)	*Aspergillus* sp. SCSIO 41501	Marine gorgonian *Melitodes squamata* Nutting.	Antifungal activity.	[50]
Marihysin A (**294**)	*Aspergillus* sp. SCSIO 41501	Marine gorgonian *Melitodes squamata* Nutting	-	[50]
**Cyclic nonapeptides**				
Clonostachysin A (**295**)	*Clonostachys rogersoniana* (HJK9)	Marine sponge *Halicondria japonica*.	Anti-dinoflagellate activity.	[133]
Clonostachysin B (**296**)	*Clonostachys rogersoniana* (HJK9)	Marine sponge *Halicondria japonica*.	Anti-dinoflagellate activity.	[133]
**Cyclic decapeptides**				
Auyuittuqamide A (**297**)	*Sesquicillium microsporum* RKAG 186	Marine sediment (intertidal zone).	-	[134]
Auyuittuqamide B (**298**)	*Sesquicillium microsporum* RKAG 186	Marine sediment (intertidal zone).	-	[134]
Auyuittuqamide C (**299**)	*Sesquicillium microsporum* RKAG 186	Marine sediment (intertidal zone).	-	[134]
Auyuittuqamide D (**300**)	*Sesquicillium microsporum* RKAG 186	Marine sediment (intertidal zone).	-	[134]
**Linear depsipeptides**				
Waspergillamide A (**301**)	*Aspergillus* sp. CMB-W031	Marine mud dauber wasp *Sceliphron* sp.	-	[135]
**Cyclic depsipeptides**				
Saroclide A (**302**)	*Sarocladium kiliense* HDN11-112	Mangrove plant *Aricennia marina*	Lipid-lowering activity.	[136]
Saroclide B (**303**)	*Sarocladium kiliense* HDN11-112	Mangrove plant *Aricennia marina*	-	[136]
Acremolide A (**304**)	*Acremonium* sp. (MST-MF588a)	Estuarine sediment.	-	[137]
Acremolide B (**305**)	*Acremonium* sp. (MST-MF588a)	Estuarine sediment.	-	[137]
Acremolide C (**306**)	*Acremonium* sp. (MST-MF588a)	Estuarine sediment.	-	[137]
Acremolide D (**307**)	*Acremonium* sp. (MST-MF588a)	Estuarine sediment.	-	[137]
Calcaripeptide A (**308**)	*Calcarisporium* sp. strain KF525	Sea.	-	[138]
Calcaripeptide B (**309**)	*Calcarisporium* sp. strain KF525	Sea.	-	[138]
Calcaripeptide C (**310**)	*Calcarisporium* sp. strain KF525	Sea.	-	[138]
Beauvericin (**311**)	*Aspergillus terreus* (no. GX7-3B)	Marine mangrove *Bruguiera gymnoihiza* (Linn.) Savigny.	Anti-acetylcholinesterase and cytotoxic activity.	[140]
	*Fusarium* sp. (No.DZ27)	Marine mangrove.	Cytotoxic activity and induction of apoptosis.	[141]
Guangomide A (**312**)	Strain No. 001314c	A yellow fan-shaped marine sponge (Coll. No. 00314).	Antibacterial activity.	[142]
Guangomide B (**313**)	Strain No. 001314c	A yellow fan-shaped marine sponge (Coll. No. 00314).	Antibacterial activity.	[142]
Homodestcardin (**314**)	Strain No. 001314c	A yellow fan-shaped marine sponge (Coll. No. 00314).	-	[142]
Sansalvamide A (**315**)	*Fusarium* sp.	Marine seagrass *Halodule wrightii*	Cytotoxic activity.	[145,146]
*N*-methylsansalvamide (**316**)	*Fusarium* sp. CNL-619	Marine mangrove green alga *Avrainvillea* sp.	Cytotoxic activity.	[146]
Acremonamide (**317**)	*Acremonium* sp. CNQ-049	Marine sediment.	Wound healing activity.	[147]
Zygosporamide (**318**)	*Zygosporium masonii* (CNK458)	Marine cyanobacterium.	Cytotoxic activity.	[148]
Alternaramide (**319**)	*Altenaria* sp. SF-5016	Marine shoreline sediment.	Antibacterial and inhibition of protein tyrosine phosphatase 1B activity.	[149]
Destruxin A (**320**)	*Metarrhizium* sp. (strain number 001103)	Marine sponge *Pseudoceratina purpurea* (coll. no. 00103).	Cytotoxic activity.	[53]
Destruxin B (**321**)	*Metarrhizium* sp. (strain number 001103)	Marine sponge *Pseudoceratina purpurea* (coll. no. 00103).	-	[53]
Destruxin B2 (**322**)	*Metarrhizium* sp. (strain number 001103)	Marine sponge *Pseudoceratina purpurea* (coll. no. 00103).	Cytotoxic activity.	[53]
Desmethyldestruxin B (**323**)	*Metarrhizium* sp. (strain number 001103)	Marine sponge *Pseudoceratina purpurea* (coll. no. 00103).	Cytotoxic activity.	[53]
Destruxin E chlorohydrin (**324**)	*Metarrhizium* sp. (strain number 001103)	Marine sponge *Pseudoceratina purpurea* (coll. no. 00103).	Cytotoxic activity.	[53]
Destruxin E2 chlorohydrin (**325**)	*Metarrhizium* sp. (strain number 001103)	Marine sponge *Pseudoceratina purpurea* (coll. no. 00103).	Cytotoxic activity.	[53]
Aspergillicin A (**326**)	*Aspergillus carneus* (MST-MF156)	Marine estuarine sediment.	-	[71]
Aspergillicin B (**327**)	*Aspergillus carneus* (MST-MF156)	Marine estuarine sediment.	-	[71]
Aspergillicin C (**328**)	*Aspergillus carneus* (MST-MF156)	Marine estuarine sediment.	-	[71]
Aspergillicin D (**329**)	*Aspergillus carneus* (MST-MF156)	Marine estuarine sediment.	-	[71]
Aspergillicin E (**330**)	*Aspergillus carneus* (MST-MF156)	Marine estuarine sediment.	-	[71]
Scopularide A (**331**)	*Scopulariopsis brevicaulis* NCPF 2177	Marine sponge *Tethya aurantium.*	Cytotoxic activity.	[151]
	*Aspergillus flavus*	Marine soft coral *Sarcophyton ehrenbergi.*	Larvicidal and inhibition of **pancreatic** lipase activity.	[152]
	*Nigrospora oryzae* PF18	Marine sponge *Phakellia fusca*	-	[153]
	*Penicillium chrysogenum* (CHSCLM-0003)	Marine gorgonian coral *Carijoa* sp. (GX-WZ-2010001).	Cytotoxic activity.	[159]
Scopularide B (**332**)	*Scopulariopsis brevicaulis* NCPF 2177	Marine sponge *Tethya aurantium.*	Cytotoxic activity.	[151]
	*Aspergillus flavus*	Marine soft coral *Sarcophyton ehrenbergi.*	Larvicidal and inhibition of **pancreatic** lipase activity.	[152]
	*Penicillium chrysogenum* (CHSCLM-0003)	Marine gorgonian coral *Carijoa* sp. (GX-WZ-2010001).	Cytotoxic activity.	[159]
Oryzamide A (**333**)	*Nigrospora oryzae* PF18	Marine sponge *Phakellia fusca.*	-	[153]
Oryzamide B (**334**)	*Nigrospora oryzae* PF18	Marine sponge *Phakellia fusca.*	-	[153]
Oryzamide C (**335**)	*Nigrospora oryzae* PF18	Marine sponge *Phakellia fusca.*	-	[153]
Oryzamide D (**336**)	*Nigrospora oryzae* PF18	Marine sponge *Phakellia fusca.*	-	[153]
Oryzamide E (**337**)	*Nigrospora oryzae* PF18	Marine sponge *Phakellia fusca.*	-	[153]
Isaridin G (**338**)	*Beauveria feline* EN-135	An unidentified marine bryozoan.	Antibacterial activity.	[157]
Desmethylisaridin G (**339**)	*Beauveria feline* EN-135	An unidentified marine bryozoan.	Antibacterial activity.	[157]
Desmethylisaridin C1 (**340**)	*Beauveria feline* EN-135	An unidentified marine bryozoan.	Antibacterial activity.	[157]
Isaridin A (**341**)	*Beauveria feline* EN-135	An unidentified marine bryozoan.	-	[157]
Isaridin B (**342**)	*Beauveria feline* EN-135	An unidentified marine bryozoan.	-	[157]
Isaridin E (**343**)	*Beauveria feline* EN-135	An unidentified marine bryozoan.	Antibacterial activity.	[157]
	*Beauveria feline* SYSU-MS7908	Marine ascidian *Styela plicata.*	-	[161]
Chrysogeamide A (**344**)	*Penicillium chrysogenum* (CHSCLM-0003)	Marine gorgonian coral *Carijoa* sp. (GX-WZ-2010001).	Anti-angiogenesis activity.	[159]
Chrysogeamide B (**345**)	*Penicillium chrysogenum* (CHSCLM-0003)	Marine gorgonian coral *Carijoa* sp. (GX-WZ-2010001).	Anti-angiogenesis activity.	[159]
Chrysogeamide C (**346**)	*Penicillium chrysogenum* (CHSCLM-0003)	Marine gorgonian coral *Carijoa* sp. (GX-WZ-2010001).	-	[159]
Chrysogeamide D (**347**)	*Penicillium chrysogenum* (CHSCLM-0003)	Marine gorgonian coral *Carijoa* sp. (GX-WZ-2010001).	-	[159]
Chrysogeamide E (**348**)	*Penicillium chrysogenum* (CHSCLM-0003)	Marine gorgonian coral *Carijoa* sp. (GX-WZ-2010001).	-	[159]
Chrysogeamide F (**349**)	*Penicillium chrysogenum* (CHSCLM-0003)	Marine gorgonian coral *Carijoa* sp. (GX-WZ-2010001).	-	[159]
Chrysogeamide G (**350**)	*Penicillium chrysogenum* (CHSCLM-0003)	Marine gorgonian coral *Carijoa* sp. (GX-WZ-2010001).	-	[159]
Nodupetide (**351**)	*Penicillium chrysogenum* (CHSCLM-0003)	Marine gorgonian coral *Carijoa* sp. (GX-WZ-2010001).	Antibacterial and anti-angiogenesis activities.	[159]
FJ120DPA (**352**)	*Aspergillus ochraceopetaliformis.*	Marine sediment.	**Inhibition of sortase A (SrtA) activity.**	[51]
Emericellamide A (**353**)	*Emericella* sp. (strain CNL-878)	Marine green alga *Halimeda* sp.	Antibacterial and cytotoxic activities.	[160]
Emericellamide B (**354**)	*Emericella* sp. (strain CNL-878)	Marine green alga *Halimeda* sp.	Antibacterial and cytotoxic activities.	[160]
Scopularide I (**355**)	*Aspergillus sclerotiorum* SCSIO41031	Marine soft coral.	Anti-acetylcholinesterase and cytotoxic activities.	[120]
Asperflosamide (**356**)	*Aspergillus flocculosus* 16D-1	Marine sponge *Phakellia fusca*.	Inhibition of tankyrase1/2 activity.	[113]
Isaridin I (**357**)	*Beauveria feline* SYSU-MS7908	Marine ascidian *Styela plicata.*	-	[161]
Isaridin J (**358**)	*Beauveria feline* SYSU-MS7908	Marine ascidian *Styela plicata.*	Antifungal activity.	[161]
Isaridin K (**359**)	*Beauveria feline* SYSU-MS7908	Marine ascidian *Styela plicata.*	-	[161]
Isaridin L (**360**)	*Beauveria feline* SYSU-MS7908	Marine ascidian *Styela plicata.*	Antifungal activity.	[161]
Isaridin M (**361**)	*Beauveria feline* SYSU-MS7908	Marine ascidian *Styela plicata.*	-	[161]
Isaridin N (**362**)	*Beauveria feline* SYSU-MS7908	Marine ascidian *Styela plicata.*	-	[161]
IB-01212 (**363**)	*Clonostachys* sp. ESNA-A009	An unidentified marine sponge.	Cytotoxic activity.	[162]
	*-*	-	Anti-parasite activity.	[166]
Phaeosphamides A (**364**)	*Phaeosphaeriopsis* sp. Esf-30	Rhizosphere sediment of a mangrove plant *Bruguiera gymnorhiza.*	Cytotoxic activity.	[164]
Phaeosphamides B (**365**)	*Phaeosphaeriopsis* sp. Esf-30	Rhizosphere sediment of a mangrove plant *Bruguiera gymnorhiza.*	-	[164]
Sch 217048 (**366**)	*Phaeosphaeriopsis* sp. Esf-30	Rhizosphere sediment of a mangrove plant *Bruguiera gymnorhiza.*	-	[164]

## Data Availability

Data sharing is not applicable.
